# Modifiable risk factors of acute kidney injury after liver transplantation: a systematic review and meta-analysis

**DOI:** 10.1186/s12882-021-02360-8

**Published:** 2021-04-23

**Authors:** Jian Zhou, Xueying Zhang, Lin Lyu, Xiaojun Ma, Guishen Miao, Haichen Chu

**Affiliations:** 1 Department of Anesthesiology, The Affiliated Hospital of Qingdao University, School of Clinical Medicine, Qingdao University, No. 59, Haier Road, Qingdao, 266100 Shandong Province China; 2 Department of Anesthesiology, West China Hospital, Sichuan University, Chengdu, 610000 Sichuan Province China

**Keywords:** Acute kidney injury, Liver transplantation, Meta-analysis, Modifiable risk factors

## Abstract

**Background:**

Acute kidney injury (AKI) is a common and critical complication of liver transplantation (LT), which is associated with increased morbidity, mortality and health care cost. We aimed to identify modifiable risk factors of AKI after LT.

**Methods:**

A literature search of Pubmed, EMBASE and Cochrane Databases was performed to identify studies investigating risk factors of AKI after LT. The Newcastle-Ottawa Scale was used to rate study quality. Effect size and 95% confidence interval were pooled using a random-effect model with inverse-variance method.

**Results:**

Sixty-seven articles with 28,844 patients were included in the meta-analysis. Seventeen modifiable risk factors were found, including overweight, preoperative use of diuretic, preoperative anemia, donation after cardiac death organ, donor BMI ≥ 30 kg/m^2^, ABO-incompatible LT, low graft to recipient body weight ratio, intraoperative hypotension, major bleeding, intraoperative use of vasopressor, large RBC transfusion, postreperfusion syndrome, postoperative use of vasopressors, overexposure to calcineurin inhibitor, calcineurin inhibitor without mycophenolate mofetil, graft dysfunction and infection. A total of 38 articles were included in the systematic review, in which 8 modifiable risk factors and 1 protective factor were additionally associated in single studies with the incidence of AKI after LT.

**Conclusions:**

Effective interventions based on identified modifiable risk factors in the perioperative management and graft allocation and preservation may be promising to reduce the incidence of AKI after LT.

**Trial registration:**

The protocol for this systematic review is registered with PROSPERO (No. CRD42020166918).

**Supplementary Information:**

The online version contains supplementary material available at 10.1186/s12882-021-02360-8.

## Background

Acute kidney injury (AKI) is rapid functional or structural kidney abnormality characterized by increased serum creatinine (Scr) or decreased urine volume [1]. The definition of AKI has evolved rapidly from Risk, Injury, Failure, Loss of kidney function, End-stage renal failure (RIFLE) criteria, Acute Kidney Injury Network (AKIN) criteria into Kidney Disease: Improving Global Outcomes (KDIGO) criteria during the past two decades. The KDIGO criteria merges RIFLE criteria and AKIN criteria, encompasses both a relative and an absolute change of Scr and allows a short and an extended time frame for diagnosis [2]. Providing simple and practical definition of AKI, KIDGO criteria gradually became standard criteria, allowing for more consistent estimates of epidemiology. A meta-analysis demonstrated that the incidence and mortality rate of AKI was 21.6 and 23.9%, respectively [3]. These numbers are even higher for patients undergoing liver transplantation (LT), where the incidence of AKI and severe AKI requiring renal replacement therapy (RRT) after LT is up to 40.8 and 7.0%, respectively [4].

AKI is a common and critical complication of LT, which remains particularly prominent among different postoperative organ injuries [5, 6]. Evidence has indicated that even transient or subclinical AKI is known to be of substantial clinical significance [7]. Previous studies have reported that AKI after LT is not only associated with immediate complications including volume overload, metabolic acidosis, and electrolyte disturbances, but also an increased rate of inferior long-term outcomes such as mortality, graft loss, infection, chronic kidney disease (CKD), prolonged stay in the intensive care unit (ICU), and augmented hospital costs [5–7]. Although much effort has been taken to the treatment of AKI, it does not seem to reverse the natural course of AKI syndrome and effectively improve prognosis [8]. AKI is increasingly recognized as a disease process with continuum of kidney injury instead of a single-hit or freestanding condition [1]. With in-depth research, the past decades have witnessed the shift of attention from treatment to prediction and early detection to avoid repetitive hits and additional damage. Thus, investigating the modifiable risk factors of AKI after LT is of vital importance.

In the past few years, a number of risk factors and predictors of AKI after LT have been reported. However, some of the conclusions remain conflicting and the role of modifiable factors are understudied with insufficient supportive evidence. So far, no comprehensive meta-analysis regarding the modifiable risk factors of AKI after LT has been conducted. Therefore, we performed a systematic review and meta-analysis via an extensive search of observational studies to identify the modifiable risk factors of AKI after LT.

## Methods

This systematic review and meta-analysis was conducted according to the Preferred Reporting Items for Systematic Reviews and Meta-Analysis (PRISMA) statement (see Supplementary 1, Additional File:PRISMA 2009 Checklist) and Meta-analysis Of Observational Studies in Epidemiology (MOOSE) [9, 10]. The protocol for this systemic review is registered with the international prospective register of systemic reviews (PROSPERO) (No. CRD42020166918).

### Study identification and search strategy

A literature search of Pubmed, EMBASE and Cochrane was performed to identify articles reporting the risk factors of AKI in patients undergoing LT. The research strategy consisted of the key search terms ‘liver transplantation’ and ‘acute kidney injury’ and their synonyms, as well as related Mesh terms combined by Boolean operators. The full search strategies for all databases are available in Supplementary 2, Additional File. Only studies published in English were included. In addition, a manual search for conceivably related studies using references of the included articles was also performed.

### Inclusion and exclusion criteria

The inclusion criteria were as follows: (a) observational studies including cohort, case-control and cross-sectional studies; (b) studies investigating patients undergoing LT; (c) a minimum of 1 risk factor identified as being associated with AKI after LT, studies illustrating the risk factors of RRT due to AKI after LT were also included as RRT per se is one of the diagnostic criteria of AKI; (d) studies reporting odds ratio (OR) with corresponding 95% confidence interval (CI) data or enough data to calculate these figures; (e) online full-text available publication. Studies were excluded for the following reasons: (a) studies did not include human subjects; (b) nonoriginal studies (conference abstracts, editorials, letters, reviews, meta-analysis, commentaries or case reports) and duplicated studies; (c) sample size was less than 50; (d) studies exploring new biomarkers or predictors of AKI after LT that are not clinically used. If more than one article were found to have used the same data, we chose the one with higher quality score, and where the quality score was equal, we chose the study with the larger sample size. Retrieved citations were first screened for relevance at the title and/or abstract level, studies remaining after the initial screening were appraised in the full text with respect to the aforementioned inclusion and exclusion criteria. Two authors (XYZ and LL) independently evaluated the eligibility of all studies. If there was disagreement regarding whether to include some articles, these articles would be further evaluated by a third author (JZ) and discussed in detail until an agreement was reached.

### Data extraction and quality assessment

For each article the following data were extracted when available: name of the first author, year of publication, country, cohort source, types of study design, sample size, donor type, surgical technique, baseline patient characteristics, definition and diagnostic criteria of AKI, duration of evaluation, incidence of AKI after LT, risk factor(s) studied, adjustment variables, the statistical methods used for multivariate analysis, effect size and 95% CI. When both of the univariate OR and the multivariate OR were reported in one study, only multivariate OR were extracted. If the OR was not reported, it was calculated from the original data if possible. When more than one definition of AKI were adopted to stratify study population in one study, we only extracted data with the latest diagnostic criteria. Modifiable risk factors refer to risk factors that can be modified by medical interventions or by individual behavior. Clinical variables were also included if they can be modified by medical interventions during perioperative period. Extracted data of the included studies were registered on dedicated electronic forms. The forms were piloted over the first 5 included studies for consistency and discrimination.

The quality of the included studies was assessed by the Newcastle-Ottawa Scale (NOS) [11]. The studies were judged on three broad perspectives: the selection of study populations, the comparability of the populations, and ascertainment of exposure and the outcomes of interest for case-control or cohort studies, respectively. A maximum score of 9 reflects the highest quality. No study was excluded because of a low-quality score. Two authors (XYZ and XJM) performed the data extraction and quality assessment independently. Disagreements were settled by discussion involving a third author (HCC) and consensus was reached on all items finally.

### Statistical analysis

If a risk factor was reported by at least 2 studies in a consistent manner, we would conducted a meta-analysis. Effect size and 95% CI were pooled using a random-effect model with inverse-variance method [12, 13]. I^2^ statistic and Cochran’s Q test were applied to determine the between-study heterogeneity. A value of I^2^ of 0–25% represents insignificant heterogeneity, 26–50% low heterogeneity, 51–75% moderate heterogeneity and 76–100% high heterogeneity [14]. In addition to the value of I^2^, we will also consider strength of evidence for the heterogeneity (CI, chi-squared test and/or *P* value) and the size and direction of effect in the analysis [15]. *P*-values on the Egger’s test greater than 0.05 and symmetry of the funnel plot determined the absence of publication bias (*N* ≥ 10) [16]. If significant publication bias was noted, Duval and Tweedie’s trim and fill method was used to acquire adjusted values [17]. To minimize heterogeneity, subgroup analyses by diagnostic criteria of AKI, duration of evaluation and statistical method were conducted. Meta-regression analyses (*N* ≥ 10) were also used to assess the potentially important covariates that might exert a substantial impact on between-study heterogeneity. Sensitivity analyses were performed after excluding 1 study at a time to assess the stability of the results and explore the source of heterogeneity. *P* < 0.05 was considered statistically significant except where otherwise stated. If data were not available for the meta-analysis or only 1 single study was identified for a given risk factor, these studies were only listed in this systematic review. Statistical analyses were performed using Stata Version 14.0 (StataCorp, Texas, USA).

## Results

### Literature search and study selection

**Fig. 1:**
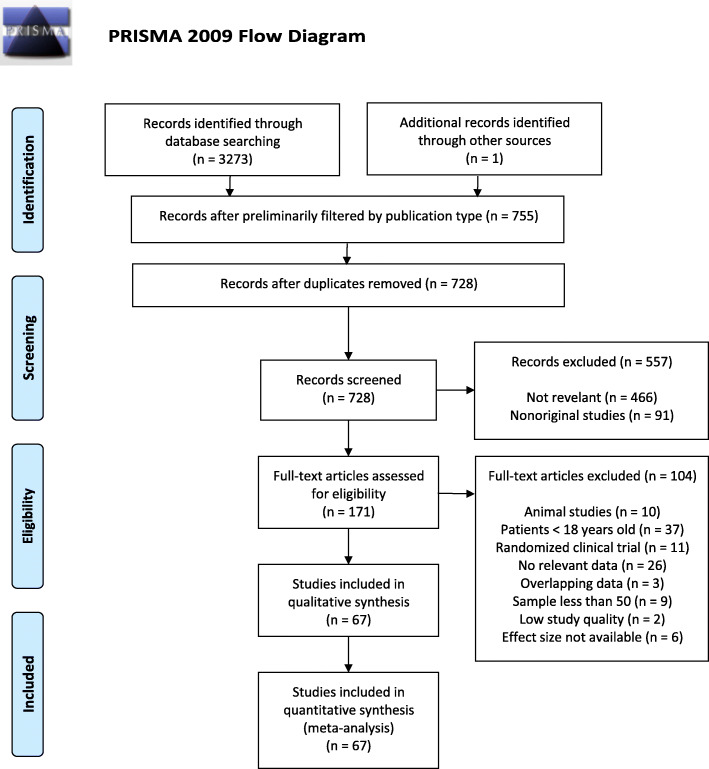
Flow diagram

A total of 3273 citations were retrieved after searching PubMed, EMBASE and CENTRAL database. There were 170 full-text articles assessed for eligibility after screening titles and abstracts. After hand-searching the references of included articles and existing reviews and meta-analyses, 1 reference was added. Three articles were excluded due to duplicate data (see Supplementary 3, Additional File). In total, 67 articles were eligible for inclusion in the systematic review and meta-analysis (see Supplementary 4, Additional File). Full details of the selection process were presented in Fig. 1.

### Characteristics of included studies and quality assessment

A total of 67 observational studies published between 2001 and 2019 with 28,844 patients were included. The incidence of AKI after LT ranged from 3.97% [18] to 71.9% [19]. Most of the included studies adopted multivariate logistic regression analysis to adjust confounding factors followed by propensity score matching method. The outcome indexes consisted of AKI, and RRT due to AKI. The RIFLE, AKIN, or KDIGO criteria were often used in combination with other scales to assess and classify AKI. The duration of evaluation varied from 12 h after reperfusion to 3 months after LT. Based on the NOS, the mean quality score of all included studies was 6.686 (standard deviation = 0.633) (see Supplementary 5, Additional File).

### Results of meta-analysis

#### Incidence of AKI after LT

**Fig. 2:**
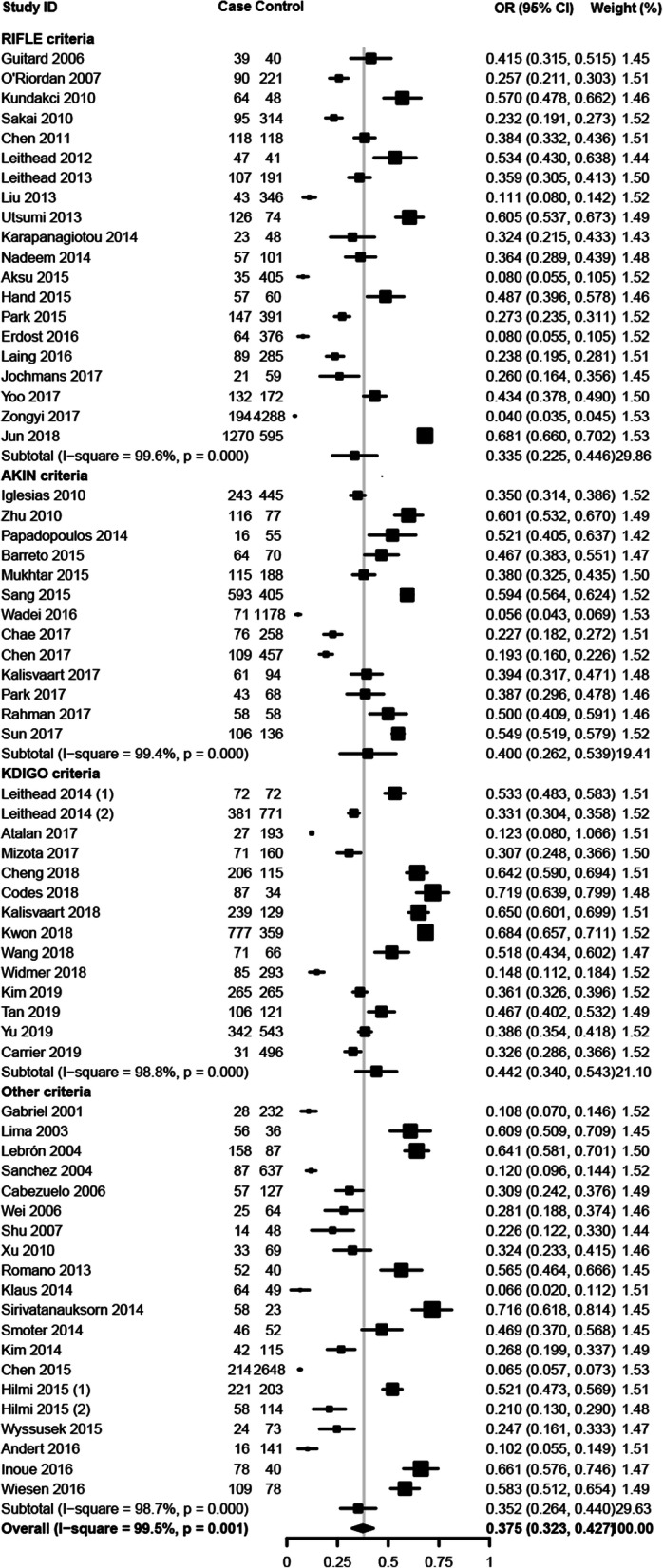
Forest plots of the included studies assessing incidence rates of AKI after LT. A diamond data marker represents the overall rate from each included study (square data marker) and 95% confidence interval

**Fig. 3:**
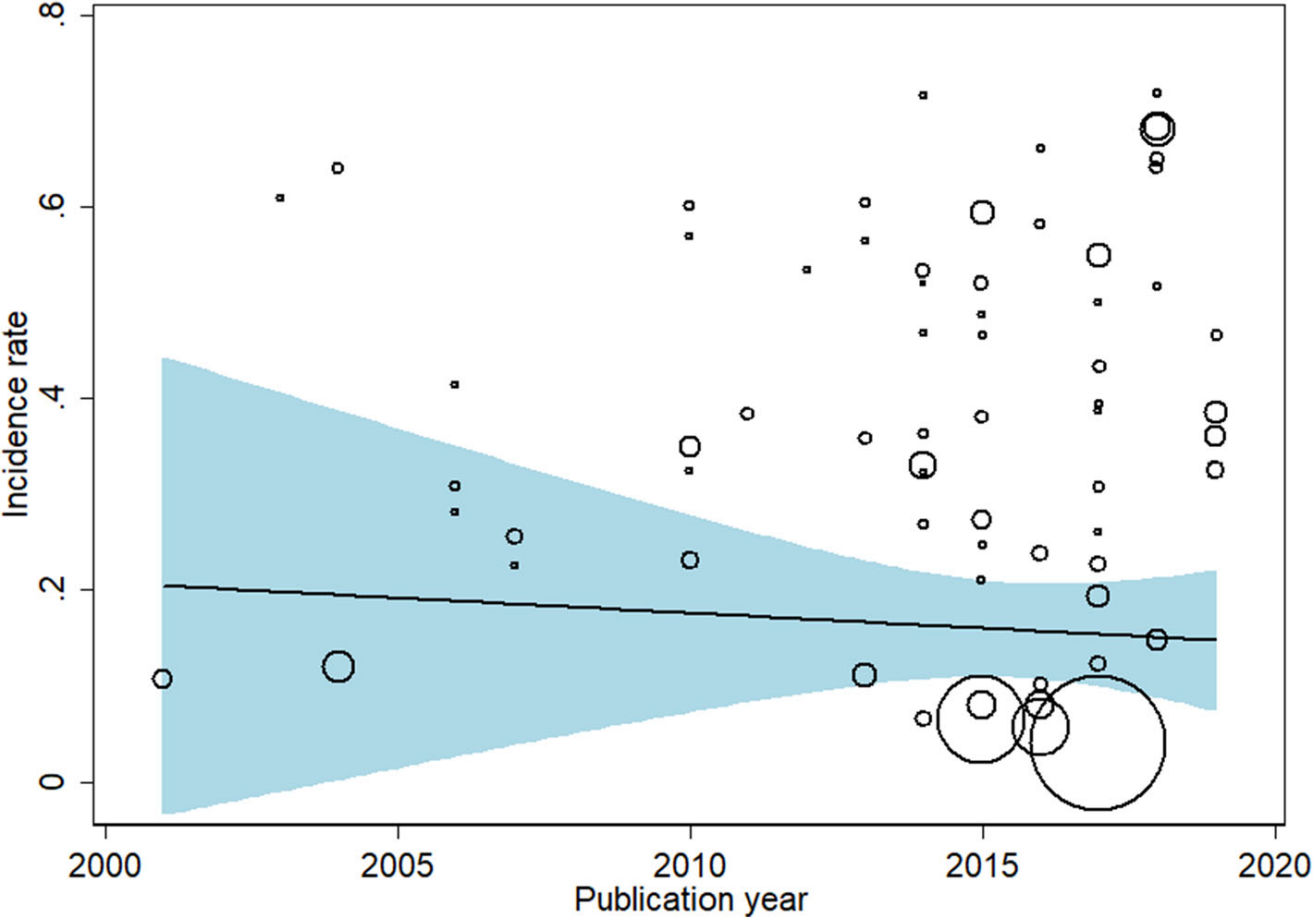
Meta-regression of incidence rate of AKI after LT on publication year

Overall, the pooled estimated incidence rate of AKI after LT was 37.5% (95% CI: 32.3–42.7%, I^2^ = 99.5%, Fig. 2). Besides, we further did a subgroup analysis based on different diagnostic criteria. The outcomes indicated that prevalence of AKI after LT was 33.5% (RIFLE), 40.0% (AKIN), 44.2% (KDIGO), and 35.2% (Others), respectively. Meta-regression showed that the publication year did not significantly affect the incidence rate of AKI after LT (*P* = 0.489, Fig. 3).

#### Modifiable risk factors of AKI after LT

**Fig. 4:**
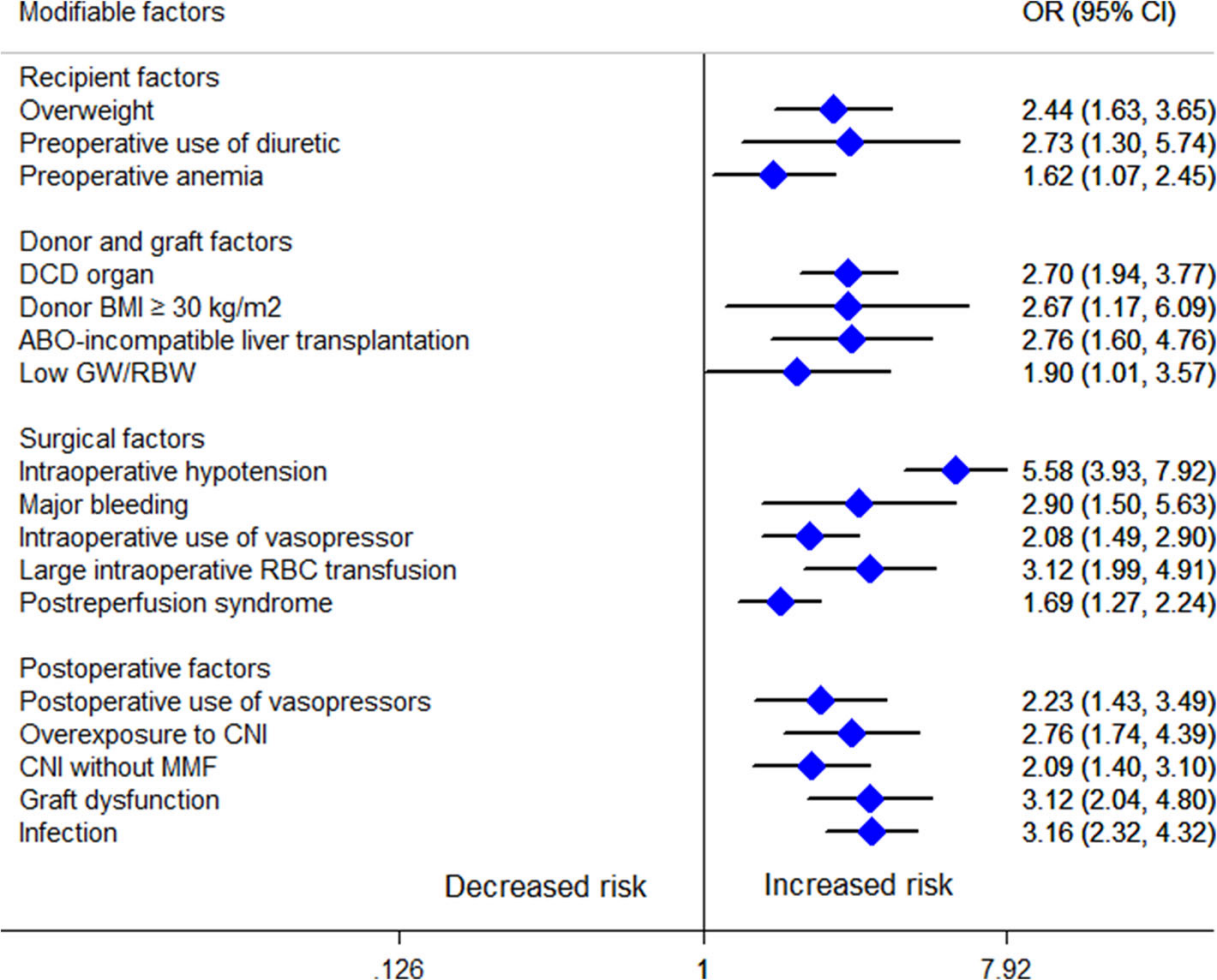
Modifiable factors that show significant association with AKI after LT in the meta-analysis. OR, odds ratio; CI, confidence interval; DCD, donation after cardiac death; BMI, body mass index; GW/RBW, graft weight to recipient body weight ratio; RBC, red blood cell; CNI: calcineurin inhibitor; MMF: mycophenolate mofetil

We pooled a forest plot for each factor that was described in at least 2 articles (see Table 1; Details are shown in Supplementary 6, Additional File). Considering the smaller pooled population (< 500) reported for some modifiable factors (cadaveric donor liver graft, intraoperative colloidal use, large postoperative red blood cell [RBC] transfusion, postoperative hypotension), here we only presented the modifiable factors with a relatively large population (> 500) to lower the error of estimates. Modifiable factors showing significant associations with AKI after LT are presented in Fig. 4. All of these factors were classified into the following 4 groups: recipient factors, donor and graft factors, surgical factors, postoperative factors.

**Table 1:** Meta-analysis of risk factors for acute kidney injury after liver transplantation

Factor	type	No. of study	Sample	Pooled OR (95% CI)	Heterogeneity		*P* value
					I^2^	Chi^2^	
Recipient factors							
Age (per year)	C	11	5476	1.006 (0.998–1.015)	78.3	0.000	0.155
Older age	B	3	380	0.956 (0.536–1.706)	0.0	0.386	0.879
Female gender	B	15	5399	1.479 (1.186–1.845)	42.5	0.042	0.001
Weight (per kg)	C	2	288	1.021 (0.996–1.046)	0.0	0.875	0.098
BMI (per kg/m^2^)	C	9	6558	1.080 (1.062–1.099)	0.0	0.904	0.000
Overweight	B	5	2420	2.437 (1.629–3.646)	56.4	0.057	0.000
White race	B	2	948	0.474 (0.303–0.740)	0.0	0.337	0.001
Hepatocellular carcinoma	B	4	1027	0.681 (0.316–1.469)	66.6	0.030	0.328
Fulminant hepatic failure	B	2	700	1.089 (0.114–10.444)	77.4	0.036	0.941
Alcoholic liver disease	B	6	2320	1.747 (1.326–2.302)	0.3	0.414	0.000
Primary biliary cirrhosis	B	2	466	0.657 (0.276–1.566)	32.3	0.224	0.343
Hepatitis B virus infection	B	3	1367	0.710 (0.486–1.039)	18.7	0.292	0.078
Hepatitis C virus infection	B	5	2930	1.113 (0.844–1.466)	11.1	0.342	0.449
Cirrhosis	B	2	887	2.171 (1.322–3.566)	0.0	0.518	0.002
Refractory ascites	B	4	685	2.293 (1.392–3.778)	0.0	0.399	0.001
Pre-existing diabetes mellitus	B	13	5763	1.390 (1.199–1.611)	0.0	0.480	0.000
Preoperative hypertension	B	10	5544	1.291 (0.814–2.045)	64.7	0.003	0.278
Preoperative use of diuretic	B	2	998	2.733 (1.302–5.739)	41.4	0.192	0.008
Child-Turcotte-Pugh grade C	B	4	2031	1.876 (1.205–2.922)	76.7	0.005	0.005
Child-Turcotte-Pugh score (per score)	C	5	2371	1.272 (1.115–1.452)	73.2	0.005	0.000
MELD (per score)	C	23	10,444	1.035 (1.024–1.045)	38.7	0.031	0.000
High MELD score	B	7	2174	1.986 (1.474–2.676)	8.7	0.363	0.000
Preoperative eGFR (per ml/min/1.73m^2^)	C	2	456	1.007 (1.000–1.015)	0.0	0.785	0.050
APACHE II (per score)	C	3	596	1.067 (1.041–1.093)	0.0	0.646	0.000
Preoperative serum creatinine (per μmol/L)	C	3	582	0.998 (0.952–1.046)	88.6	0.000	0.931
Preoperative serum creatinine (per mg/dL)	C	6	2422	2.337 (1.215–4.497)	81.0	0.000	0.011
High preoperative serum creatinine	B	5	5498	2.155 (1.219–3.811)	67.2	0.016	0.008
Preoperative serum albumin (per g/dL)	C	2	3001	0.539 (0.460–0.632)	0.0	0.740	0.000
Preoperative hypoalbuminemia	B	3	958	1.127 (0.259–4.905)	96.1	0.000	0.874
Preoperative hemoglobin (per g/dL)	C	4	4278	0.888 (0.856–0.922)	0.0	0.433	0.000
Preoperative anemia	B	3	1410	1.621 (1.073–2.449)	24.5	0.266	0.022
Donor and graft factors							
Cadaveric donor liver graft	B	2	329	3.360 (1.549–7.289)	0.0	0.927	0.002
DCD organ	B	3	1642	2.704 (1.938–3.772)	0.0	0.996	0.000
Donor age (per year)	C	5	2170	1.004 (0.991–1.017)	49.5	0.095	0.578
Older donor age	B	3	1470	1.213 (0.799–1.840)	23.6	0.270	0.364
Donor BMI ≥ 30 kg/m^2^	B	2	1309	2.672 (1.173–6.085)	57.8	0.124	0.019
Donor risk index (per point)	C	2	1404	0.820 (0.485–1.388)	0.0	0.448	0.460
ABO-incompatible liver transplantation	B	2	1274	2.761 (1.602–4.759)	0.0	0.751	0.000
Graft-recipient weight ratio	C	3	3999	0.497 (0.370–0.667)	0.0	0.830	0.000
Low graft to recipient body weight ratio	B	5	1565	1.902 (1.013–3.568)	52.3	0.078	0.045
Cold ischaemic time (per min)	C	2	358	1.000 (0.990–1.010)	0.0	1.000	1.000
Cold ischaemic time (per hour)	C	7	2887	1.064 (1.003–1.130)	48.6	0.070	0.041
Long cold ischaemic time	B	3	5220	1.408 (0.907–2.187)	73.1	0.024	0.128
Warm ischaemic time (per min)	C	7	3427	1.018 (1.007–1.029)	53.6	0.044	0.001
Long warm ischaemic time	B	3	5220	1.411 (0.711–2.799)	83.9	0.002	0.325
Surgical factors							
Piggyback surgical technique	B	3	903	0.556 (0.195–1.585)	81.8	0.004	0.272
Split liver transplantation	B	2	1296	1.074 (0.655–1.759)	34.4	0.217	0.777
Venovenous bypass	B	3	740	0.577 (0.086–3.865)	95.2	0.000	0.571
Intraoperative hypotension	B	5	566	5.582 (3.934–7.920)	0.0	0.898	0.000
Intraoperative blood loss (per liter)	C	5	1734	1.156 (1.022–1.308)	90.1	0.000	0.021
Large intraoperative blood loss	B	6	5639	2.900 (1.495–5.627)	83.1	0.000	0.002
Intraoperative use of vasopressor	B	13	4625	2.079 (1.492–2.899)	70.3	0.000	0.000
Intraoperative colloidal use	B	3	495	2.447 (1.508–3.973)	0.0	0.630	0.000
Intraoperative RBC transfusion (per unit)	C	15	8006	1.042 (1.025–1.059)	76.9	0.000	0.000
Intraoperative RBC transfusion (per liter)	C	4	2253	1.196 (1.143–1.253)	11.4	0.336	0.000
Large intraoperative RBC transfusion	B	11	3401	3.124 (1.986–4.914)	72.8	0.000	0.000
Intraoperative FFP transfusion (per unit)	C	9	5202	1.027 (1.021–1.032)	3.8	0.403	0.000
Intraoperative platelet transfusion (per unit)	C	3	1503	1.321 (0.863–2.024)	69.3	0.039	0.200
Intraoperative urine output (per mL)	C	2	777	0.995 (0.986–1.003)	96.8	0.000	0.232
Postreperfusion syndrome	B	9	4731	1.689 (1.275–2.236)	52.8	0.031	0.000
Duration of operation (per hour)	C	4	1563	1.158 (1.008–1.330)	65.9	0.032	0.038
Long operation time	B	2	738	1.485 (0.937–2.353)	0.0	0.470	0.093
Postoperative factors							
Postoperative hypotension	B	2	173	6.127 (1.871–20.067)	0.0	0.824	0.003
Large postoperative RBC transfusion	B	2	308	5.051 (2.387–10.691)	0.0	0.899	0.000
Postoperative use of vasopressor	B	4	4903	2.234 (1.431–3.488)	75.9	0.006	0.000
Postoperative peak AST (per u/L)	C	2	232	3.687 (1.081–12.575)	79.7	0.026	0.037
Postoperative peak AST (per IU/L)	C	2	196	1.451 (0.618–3.410)	84.2	0.012	0.393
Overexposure to CNI	B	2	4682	2.762 (1.737–4.391)	0.0	0.857	0.000
No combined use of mycophenolate mofetil	B	3	5220	2.087 (1.404–3.103)	0.0	0.899	0.000
Postoperative tacrolimus peak level (per ug/L)	C	3	1675	0.983 (0.951–1.017)	50.0	0.135	0.326
Postoperative tacrolimus use	B	4	2891	1.522 (0.942–2.459)	43.8	0.149	0.086
Postoperative hypoalbuminemia	B	2	1528	0.718 (0.261–1.976)	95.6	0.000	0.522
Graft dysfunction	B	3	1744	3.124 (2.036–4.795)	0.0	0.513	0.000
Infection	B	8	1651	3.162 (2.315–4.320)	0.0	0.983	0.000

*OR* odds ratio, *C* continuous data, *B* binary data, *MELD* model for end-stage liver disease, *eGFR* estimated glomerular filtration rate, *APACHE* acute physiology and chronic health evaluation, *DCD* donation after cardiac death, *BMI* body mass index, *RBC* red blood cell, *FFP* fresh frozen plasma, *AST* aspartate transaminase, *CNI* calcineurin inhibitor

#### Recipient factors

Overweight (OR = 2.437, 95% CI = 1.629–3.646, I^2^ = %, *P* = 0.000), preoperative use of diuretic (OR = 2.733, 95% CI = 1.302–5.739, I^2^ = 41.4%, *P* = 0.008), preoperative anemia (OR = 1.621, 95% CI = 1.073–2.449, I^2^ = 24.5%, *P* = 0.022) were identified as modifiable risk factors of AKI after LT. Preoperative hypertension and preoperative hypoalbuminemia were not correlated with AKI after LT.

#### Donor and graft factors

Donation after cardiac death (DCD) organ (OR = 2.704, 95% CI = 1.938–3.772, I^2^ = 0.0%, *P* = 0.000), donor body mass index (BMI) ≥ 30 kg/m^2^ (OR = 2.672, 95% CI = 1.173–6.085, I^2^ = 57.8%, *P* = 0.019), ABO-incompatible LT (OR = 2.761, 95% CI = 1.602–4.759, I^2^ = 0.0%, *P* = 0.000), low graft weight to recipient body weight ratio (GW/RBW) (OR = 1.902, 95% CI = 1.013–3.568, I^2^ = 52.3%, *P* = 0.045) increased the risk of AKI after LT. However, no significant associations were found for long cold ischemia time (CIT) and long warm ischemia time (WIT).

#### Surgical factors

Intraoperative hypotension (OR = 5.582, 95% CI = 3.934–7.920, I^2^ = 0.0%, *P* = 0.000), major bleeding (OR = 2.900, 95% CI = 1.495–5.627, I^2^ = 83.1%, *P* = 0.002), intraoperative use of vasopressor (OR = 2.079, 95% CI = 1.492–2.899, I^2^ = 70.3%, P = 0.000), large intraoperative RBC transfusion (OR = 3.124, 95% CI = 1.986–4.914, I^2^ = 72.8%, *P* = 0.000), postreperfusion syndrome (OR = 1.689, 95% CI = 1.275–2.236, I^2^ = 52.8%, *P* = 0.000) were associated with an increased risk for AKI after LT. Nevertheless, no obvious associations were detected with piggyback surgical technique, split LT, venovenous bypass and intraoperative platelet transfusion.

#### Postoperative factors

Postoperative use of vasopressors (OR = 2.234, 95% CI = 1.431–3.488, I^2^ = 75.9%, *P* = 0.000), overexposure to calcineurin inhibitor (CNI) (OR = 2.762, 95% CI = 1.737–4.391, I^2^ = 0.0%, P = 0.000), CNI without mycophenolate mofetil (MMF) (OR = 2.087, 95% CI = 1.404–3.103, I^2^ = 0.0%, P = 0.000), graft dysfunction (OR = 3.124, 95% CI = 2.036–4.795, I^2^ = 0.0%, P = 0.000), infection (OR = 3.162, 95% CI = 2.315–4.320, I^2^ = 0.0%, P = 0.000) were associated with a higher risk for AKI after LT. Neither postoperative tacrolimus use nor postoperative hypoalbuminemia showed significant association with AKI after LT.

### Sensitivity analysis, subgroup analysis, meta-regression analysis, and publication bias

Evident heterogeneity in the meta-analysis of 34 factors was found (I^2^ > 50% or *P* < 0.1), and 28 factors could be further analyzed (*N* ≥ 3). The results of the sensitivity analysis are shown in Supplementary 7, Additional File. When 1 single study was excluded each time, the heterogeneity was obviously reduced (I^2^ change > 30%) for the following 10 modifiable factors: overweight, preoperative hypoalbuminemia, low GW/RBW, long CIT, long WIT, piggyback surgical technique, venovenous bypass, large intraoperative blood loss, intraoperative platelet transfusion, postoperative use of vasopressor. The source of heterogeneity may have been due to several design differences among the studies, including sample size, diagnostic criteria, duration of evaluation, cutoff point of factors, or insufficient adjustment for confounding factors. The details are shown in Supplementary 8, Additional File.

After conducting subgroup analysis by diagnostic criteria of AKI, the heterogeneity was obviously reduced (I^2^ change > 30%) for low GW/RBW, indicating diagnostic criteria might be the source of heterogeneity. The statistical significances were changed for preoperative hypoalbuminemia after conducting subgroup analysis. In the subgroup of RIFLE, preoperative hypoalbuminemia showed statistically significant association with AKI after LT without heterogeneity (see Supplementary 9, Additional File).

After conducting subgroup analysis by duration of evaluation, no obvious reduction of heterogeneity (I^2^ change > 30%) was observed for the aforementioned factors. However, the statistical significances were changed for several modifiable factors (see Supplementary 10, Additional File). In the subgroup of ≤7 days, intraoperative platelet transfusion showed association with AKI after LT, whereas low GW/RBW showed no association with AKI after LT; in the subgroup of > 7 days, major bleeding, large intraoperative RBC transfusion, postoperative use of vasopressor showed no association with AKI after LT.

After conducting subgroup analysis by statistical method, no obvious reduction of heterogeneity (I^2^ change > 30%) was observed for the aforementioned factors, indicating statistical method was not the source of heterogeneity of aforementioned modifiable risk factors (see Supplementary 11, Additional File).

We also performed meta-regression analyses for factors with evident heterogeneity (see Supplementary 12, Additional File), and statistical method may partially explain the source of heterogeneity in intraoperative use of vasopressor. Based on the results from the funnel plot and Egger test (see Supplementary 13, Additional File), no evidence for publication bias was detected in factors described in ≥10 studies.

### Systematic review

**Fig. 5:**
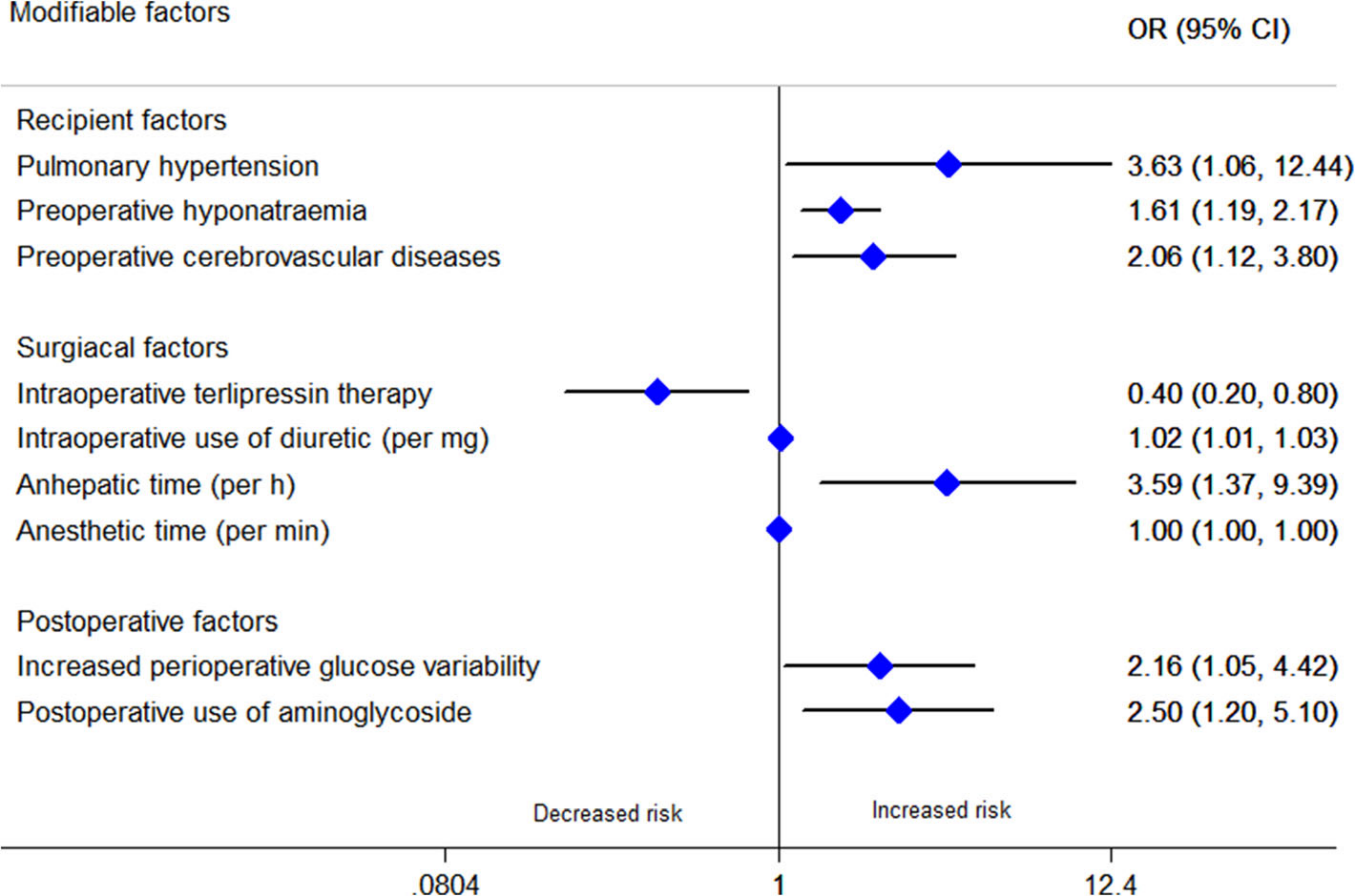
Modifiable factors that show significant association with AKI after LT in the systematic review. OR, odds ratio; CI, confidence interval

A total of 75 factors were included only in the systematic review rather than the meta-analysis (see Supplementary 14, Additional File) because the assessment was performed in only 1 study. A total of 15 risk factors and 1 protective factor were associated with AKI after LT (here we only presented the factors adjusted for confounding factors with a relatively large population [> 300] to lower the error of estimates) (Fig. 5). In this systematic review, additional modifiable risk factors of AKI after LT were preoperative hyponatraemia, preoperative cerebrovascular diseases, pulmonary hypertension, increased perioperative glucose variability, long anesthetic time, intraoperative use of diuretic, long anhepatic time and postoperative aminoglycoside use. Besides, patients with intraoperative terlipressin therapy were associated with a decreased risk for AKI after LT.

## Discussion

### Main findings

**Fig. 6:**
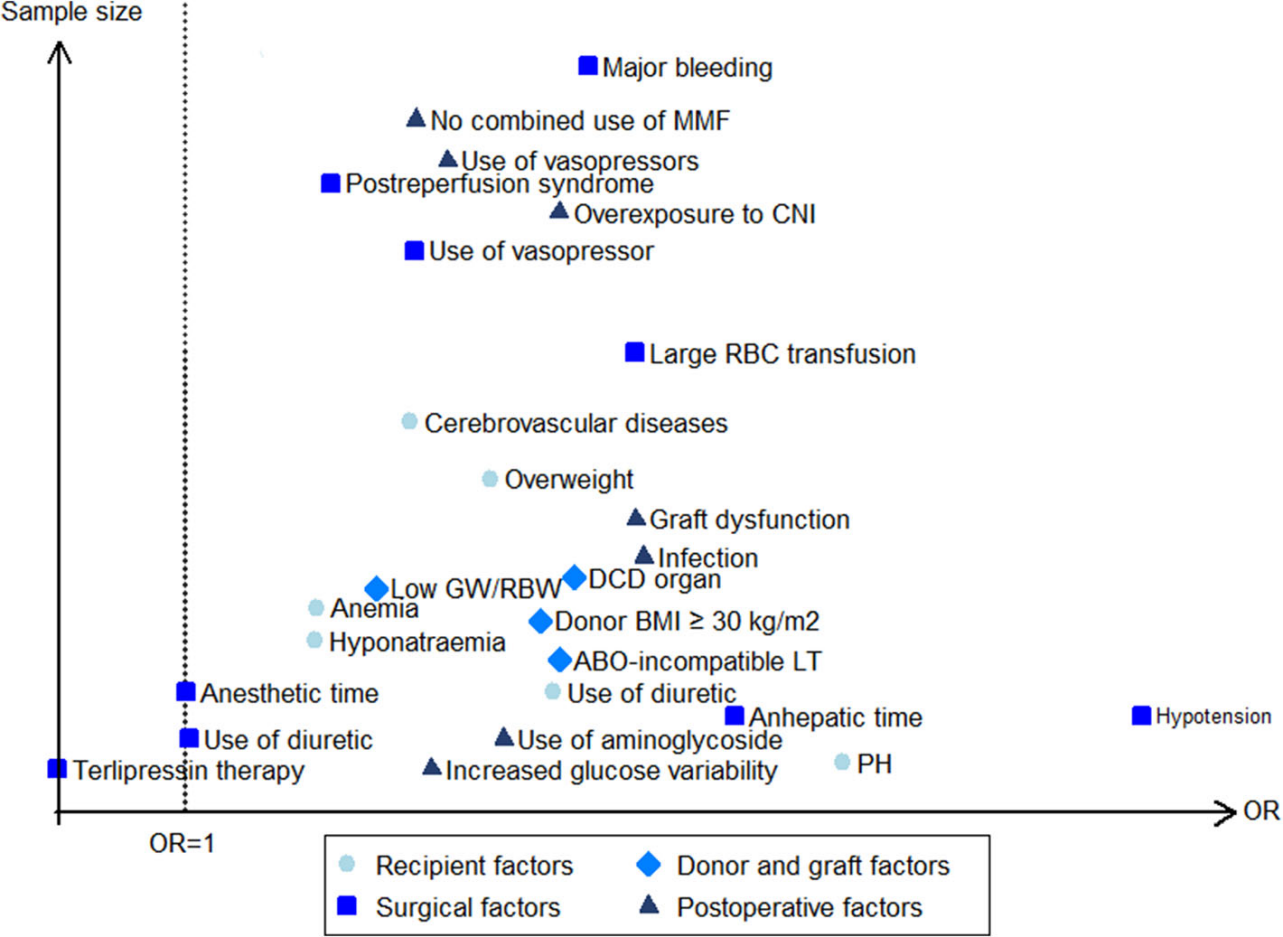
Identified modifiable factors of AKI after LT. OR, odds ratio; DCD, donation after cardiac death; BMI, body mass index; GW/RBW, graft weight to recipient body weight ratio; RBC, red blood cell; CNI: calcineurin inhibitor; MMF: mycophenolate mofetil; LT, liver transplantation; PH, pulmonary hypertension

In this systematic review and meta-analysis we have identified the modifiable risk factors of AKI after LT for the first time. Our work involved extensive analyses and shed new light on early identification and preventive strategies for AKI after LT. A total of 25 modifiable risk factors and 1 protective factor of AKI after LT were found (Fig. 6), of which 17 factors had data eligible for meta-analysis, including overweight, preoperative use of diuretics, preoperative anemia, DCD organ, donor BMI ≥ 30 kg/m^2^, ABO-incompatible LT, low GW/RBW, intraoperative hypotension, major bleeding, intraoperative use of vasopressor, large intraoperative RBC transfusion, postreperfusion syndrome, postoperative use of vasopressor, overexposure to CNI, CNI without MMF, graft dysfunction and infection (Fig. 4).

### Explanation of results

It is challenging to explore modifiable risk factors of AKI after LT because of numerous and heterogenous diagnostic criteria. Both criteria use Scr and urine output as markers of renal function [20]. Considering the retrospective nature of most observational studies, all included studies uniformly use Scr only to classify AKI after LT as detailed hourly urine output is often not available. The between-study heterogeneity may be the result of discrepancies in definition and classifications, various cutoff points of factors and different duration of evaluation. In addition, Scr can be influenced by dietary, volume overload, body muscle-mass and liver function [6]. In fact, Scr is a marker of renal function instead of kidney injury and can be delayed and insensitive under some circumstances [1]. As patients awaiting LT tend to have a reduced creatinine production compared with healthy subjects and fluid accumulation might mask the increase in Scr, it is likely that Scr overestimates the severity of preoperative renal function and delayed diagnosis and underestimates the severity of postoperative AKI after LT. Another issue is the relevance of baseline Scr to the perioperative period. On the one hand, over-diagnosis can occur when the immediate Scr or the Scr after fluid resuscitation are selected as baseline Scr; on the other hand, comparing Scr after massive fluid administration in the postoperative period to preoperative baseline Scr can lead to under-diagnosis of AKI [21]. Thus, the diagnosis of AKI after LT faces challenges, and new biomarkers and adoption of standard definition are warranted.

That AKI after LT are multifarious in etiology is undeniable. As for recipient factors, overweight patient are at increased risk of AKI after LT, which is consistent with the findings of other clinical settings [22, 23]. Patients with high BMI are more likely to suffer from metabolic syndromes, including hypertension, dyslipidemia, cardiovascular and cerebrovascular diseases. Metabolic syndromes and obesity-related glomerulopathy may provide a vulnerable physiological reserve to handle the stress of hypoperfusion of kidney during surgery [24]. Though loop diuretic is widely used in different stages of AKI, data from randomized controlled trials and observational studies concerning the theoretical advantage of diuretics in preventing and treating AKI remain controversial and unproven [25–27]. Thus use of diuretics can lead to renal damage and nephrotoxicity and is recommended only if volume overload exists [28, 29]. Anemia may contribute to AKI through reducing the oxygen capacity of blood, enhancing oxidative stress and impairing hemostasis [30]. Therefore detecting and optimizing recipient preoperative hemoglobin status as early as possible is highly recommended in established consensus [31]. It is worth mentioning that sensitivity analysis revealed that the Cabezuelo (2006) study was the source of statistical heterogeneity in the meta-analysis for the association of preoperative hypoalbuminemia with AKI after LT. When this outlying study is removed, there was no evidence of heterogeneity in the remaining studies and the result showed that preoperative hypoalbuminemia increased the risk of AKI after LT (OR = 2.134, 95% CI = 1.412–3.225, I^2^ = 0.0%, *P* = 0.000), which was broadly in line with previous studies cross different clinical settings [32]. The mechanism for the association of preoperative hypoalbuminemia with AKI after LT remains elusive, serum albumin may decrease the risk of AKI by maintaining renal perfusion, binding endogenous toxins and nephrotoxic drugs, alleviating oxidative damage, and delivering protective lysophosphatidic acid [32]. However, the association of serum albumin level and AKI might be U-shape [33], and there is evidence that administration of exogenous albumin failed to alter renal outcomes in the clinical scenario of living donor liver transplantation [34]. Hypotheses are proposed that not hypoalbuminemia itself but rather the underlying causes of it affect the occurrence of AKI after LT [6]. It appears that DCD organ is an independent risk factor for AKI after LT. Leithead hypothesized that hepatic ischaemia reperfusion injury (HIRI) is a leading cause for renal injury in recipients of DCD grafts [35]. It remains questionable whether the greater graft injury is attributed to the added donor WIT as previous studies have obtained conflicting results [36]. Unexpectedly, long WIT and long CIT are not risk factors for AKI after LT in our meta-analysis, though several previous studies have proved that they showed good performance in predicting AKI after LT. We postulate that though to some extent these risk factors reflect graft quality, they may be weak in promoting the occurrence of AKI after LT compared to other risk factors. Besides, with the improvement of organ procurement techniques, long ischemia time does not mean poor graft quality. Donor obesity and graft steatosis increase the susceptibility of the liver to HIRI, which is hypothesized as one of the driving forces of AKI after LT [35]. The exact mechanism for AKI in ABO-incompatible LT patients has not been elucidated, probably owing to plasmapheresis, high isoagglutinin titer and enhanced immunosuppression [37, 38]. Low GW/RBW precipitates patients to persistent portal hypertension and a hyperdynamic state, which may impair the balance between vasodilatory and vasoconstrictor factors and lead to AKI after LT [39]. Overall these findings suggest that graft quality and HIRI may play an important role in the development of AKI after LT and optimizing graft quality and limiting HIRI is feasible measures to prevent AKI after LT.

With regard to surgical factors, our study indicates that intraoperative hypotension, major bleeding, use of vasopressor, large RBC transfusion, and postreperfusion syndrome (PRS) during operation are associated with increased risk of AKI after LT. Those variables reflect that hemodynamic instability, which exerts a major effect on reduction in renal blood flow and renal tissue hypoxia, may play a leading role in the development of AKI after LT. Notably, intraoperative hypotension had the highest OR as a modifiable risk factor for AKI after LT. Hypoperfusion and subsequent inflammation and neuroendocrine response to surgery are the frequent mechanisms affecting renal perfusion during perioperative period [2]. Only when the mean arterial pressure is above the autoregulatory threshold can the kidney maintain glomerular filtration rate in the face of unstable arterial pressure and changing volume status. PRS leads not only to the release of cold and acidotic components by the graft but also pro-inflammatory cytokines that trigger inflammatory response and subsequent renal tubular injury [5]. In this regard, AKI after LT crosses the boundaries of traditional pathophysiological categories and encompasses pre-renal AKI and intrinsic renal AKI. Besides, RBC transfusion can induce oxidative stress and systemic inflammatory response syndrome, impair oxygen delivery and vascular regulation, release procoagulant phospholipids, and increase adhesiveness to vascular endothelium, thereby harming the kidneys [40]. In fact, many modifiable risk factors are interrelated and preventive strategies targeting multiple modifiable risk factors are crucially needed. Therefore, maintaining normovolaemia, reducing blood loss and avoiding unnecessary blood transfusion are of utmost importance for preventing the occurrence of AKI after LT. In addition, it is noteworthy that apart from norepinephrine, vasopressors like epinephrine, dopamine or phenylephrine did not show clinical benefit of renoprotective [41].

Concerning postoperative factors, patients with AKI after LT seemed to have more aggressive immunosuppressive regimen, more often had graft dysfunction, and experienced more hemodynamic insults immediately after operation. The relationship between graft dysfunction and AKI after LT is being increasingly recognized. As for graft dysfunction, evidences from animal experiments have shown that renal cells go through apoptosis during ischaemia reperfusion injury like hepatic cells after LT [42]. Intravascular oxidative stress and functional impairment of the mitochondria can trigger a systemic inflammatory response, thus the deleterious effects not only impair the liver, but also the kidney [43]. In this regard, AKI after LT is not only a pathological condition of single organ failure but also can be seen as a marker of multi-organ injury [4], as there is increasing evidence that AKI directly contributes to remote organ injury and plays an active role in the progression of multi-organ dysfunction [2, 44]. Sepsis is the most frequent cause of AKI in the inpatient population and systemic inflammation resulting in tubular injury can account for sepsis-associated AKI irrespective of ischaemia as an initiating factor [45]. CNIs remain the mainstay of immunosuppression regimens after LT in spite of the well recognized nephrotoxicity. Renal artery vasoconstriction and development of thrombotic microangiopathy are presumed mechanism of CNI related injury [5]. Our work indicated that reduced or delayed CNI regimen combined with MMF decreased the incidence of AKI of LT. Overall tailoring the immunosuppression regimen in recipients at high-risk of developing severe AKI should be first priority in the immediate postoperative period.

The presence of heterogeneity was as expected, which may be due to the differences between individual studies, such as small sample size (postoperative use of vasopressor), different diagnostic criteria (intraoperative platelet transfusion), relatively long duration of evaluation (preoperative hypoalbuminemia, large intraoperative blood loss), relatively high cutoff point of definition (low GW/RBW, long CIT) and insufficient adjustment for confounding factors. Therefore, we conducted specific analyses based on clinical and methodological characteristics of studies and adjusted for the heterogeneity as much as possible. Recruiting larger samples with unified diagnostic criteria and searching for appropriate statistical methods to adjust for confounding factors are necessary in future studies.

### Future directions

Our study raised several questions that need to be addressed in future studies: (1) recipients with different baseline characteristics might show different sensitivity to perioperative precipitating factors and interventions. Therefore, risk stratification for AKI after LT using baseline characteristics is warranted in the future studies and clinical practice. (2) Considering that Scr can only serve as a retrospective marker of kidney function, it is reasonable to imagine that we might detect an ideal biomarker like highly sensitive troponin in the diagnosis of myocardial injury which is easily identifiable and will provide more timely and specific diagnosis of AKI in the near future. (3) At present, most risk prediction models for AKI after LT are lack of external validation in large multicentre cohorts, which significantly limits their clinical implementation. Therefore, finding reliable risk prediction tools (e.g. deep learning model [46] and automated clinical decision support systems [47]) using routinely measured variables continues to be intensive areas of research. (4) It is necessary to establish nonpharmacologic interventions specific for LT recipients based on the identified modifiable risk factors to improve prognosis of AKI after LT. To enable this, multidisciplinary cooperation including surgeons, anesthesiologists, and nephrologists are crucial in both clinical practice and future randomized controlled studies.

### Strengths and limitations

There are several limitations in our study that should be addressed. First, only observational studies are included, thus some high-quality randomized controlled trial studies must have been missed, and our results cannot establish a cause-and-effect relationship. Second, studies included in this review represented a wide range of LT experience, institutional routines, and other possible existence of unknown or unmeasured factors that might influence the heterogeneity and potentially hamper the generalizability of the results. Third, different risk factors and predictors reported in various studies constrain us from accurately describing them in a similar manner. Fourth, not all studies made enough adjustment for confounding factors or provide precise effect size of multivariate analysis, and we can not fully unify the confounding factors due to the large number of studies. Fifth, inevitable heterogeneity may preclude conclusions regarding some factors that are likely to increase or decrease the development of AKI after LT. Finally, although efforts are made to eliminate duplicate data by title, author, cohort source, patient recruitment time, donor type and surgical technique, overlapping data may have been included in our study.

This is the first comprehensive systematic review and meta-analysis taking into account all modifiable risk factors of AKI after LT. The numerous modifiable risk factors could lay foundation for risk stratification, early identification, and effective prevention. Further high-quality studies with larger sample sizes and randomized controlled trials targeting multimodal interventions are crucially needed.

## Conclusions

This is by far the first study to quantitatively summarize the modifiable risk factors of AKI after LT. The modifiable risk factors identified in our study include overweight, preoperative use of diuretics, preoperative anemia, DCD organ, donor BMI ≥ 30 kg/m^2^, ABO-incompatible LT, low GW/RBW, intraoperative hypotension, major bleeding, intraoperative use of vasopressor, large intraoperative RBC transfusion, postreperfusion syndrome, postoperative use of vasopressor, overexposure to CNI, CNI without MMF, graft dysfunction and infection. Effective interventions in the perioperative management and graft allocation and preservation may be promising to reduce the incidence of AKI after LT.

## Supplementary Information

**Additional file 1.**

## Data Availability

The datasets generated and/or analyzed during this study are included in this published article and its Supplementary information files.

## Abbreviations

AKI: acute kidney injury
Scr: serum creatinine
RIFLE: Risk, Injury, Failure, Loss of kidney function, End-stage renal failure
AKIN: Acute Kidney Injury Network
KDIGO: Kindney Disease: Improving Global Outcomes
LT: liver transplantation
RRT: renal replacement therapy
CKD: chronic kidney disease
ICU: intensive care unit
PRISMA: Preferred Reporting Items for Systematic Reviews and Meta-Analysis
MOOSE: Meta-analysis Of Observational Studies in Epidemiology
OR: odds ratio
CI: confidence interval
NOS: Newcastle-Ottawa Scale
RBC: red blood cell
DCD: donation after cardiac death
BMI: body mass index
GW/RBW: graft weight to recipient body weight ratio
CIT: cold ischaemia time
WIT: warm ischaemia time
CNI: calcineurin inhibitor
MMF: mycophenolate mofetil
HIRI: hepatic ischaemia reperfusion injury
PRS: postreperfusion syndrome
