# Supplementary material for: Modifiable risk factors of acute kidney injury after liver transplantation: a systematic review and meta-analysis
Source: BMC Nephrol. 2021 Apr 23;22:149. doi: 10.1186/s12882-021-02360-8 (PMC8063403; doi:10.1186/s12882-021-02360-8)
Supplement: Supplementary file 1 — Additional file 1. [file 12882_2021_2360_MOESM1_ESM.docx]

**Modifiable risk factors of acute kidney injury after liver transplantation: a systematic review and meta-analysis**

Jian Zhou, Xueying Zhang, Lin Lyu, Xiaojun Ma, Guishen Miao, Haichen Chu*

**Additional file**

**Contents：**

**Supplementary 1: PRISMA 2009 Checklist** **3**

**Supplementary 2: List of terms of the search strategy** **6**

**Supplementary 3: Duplicate data identification and elimination** **7**

**Supplementary 4: Characteristics of included studies** **10**

**Supplementary 5: Quality evaluation of included studies** **41**

**Supplementary 6: Forest plots of factors described in at least 2 studies** **44**

**Supplementary 7: Results of sensitivity analysis after excluding 1 single study** **81**

**Supplementary 8: Factors with a significant change of I^2^ after conducting sensitivity analysis** **95**

**Supplementary 9: Subgroup analysis for factors with evident heterogeneity by diagnostic criteria** **97**

**Supplementary 10: Subgroup analysis for factors with evident heterogeneity by duration of evaluation** **100**

**Supplementary 11: Subgroup analysis for factors with evident heterogeneity by statistical method** **102**

**Supplementary 12: Meta-regression of incidence and factors with evident heterogeneity** **104**

**Supplementary 13: Funnel plot and Egger test of factors described in ≥ 10 study**

**110**

**Supplementary 14: Systematic review of factors described in only one study** **118**

**Supplementary 1: PRISMA 2009 Checklist**

| **Section/topic** | **#** | **Checklist item** | **Reported on page #** |
| --- | --- | --- | --- |
| **TITLE** | | |  |
| Title | 1 | Identify the report as a systematic review, meta-analysis, or both. | 1 |
| **ABSTRACT** | | |  |
| Structured summary | 2 | Provide a structured summary including, as applicable: background; objectives; data sources; study eligibility criteria, participants, and interventions; study appraisal and synthesis methods; results; limitations; conclusions and implications of key findings; systematic review registration number. | 2 |
| **INTRODUCTION** | | |  |
| Rationale | 3 | Describe the rationale for the review in the context of what is already known. | 3-4 |
| Objectives | 4 | Provide an explicit statement of questions being addressed with reference to participants, interventions, comparisons, outcomes, and study design (PICOS). | 4 |
| **METHODS** | | |  |
| Protocol and registration | 5 | Indicate if a review protocol exists, if and where it can be accessed (e.g., Web address), and, if available, provide registration information including registration number. | 4 |
| Eligibility criteria | 6 | Specify study characteristics (e.g., PICOS, length of follow-up) and report characteristics (e.g., years considered, language, publication status) used as criteria for eligibility, giving rationale. | 5 |
| Information sources | 7 | Describe all information sources (e.g., databases with dates of coverage, contact with study authors to identify additional studies) in the search and date last searched. | 5 |
| Search | 8 | Present full electronic search strategy for at least one database, including any limits used, such that it could be repeated. | 5 (S 2, Additional File 1) |
| Study selection | 9 | State the process for selecting studies (i.e., screening, eligibility, included in systematic review, and, if applicable, included in the meta-analysis). | 5-6 |
| Data collection process | 10 | Describe method of data extraction from reports (e.g., piloted forms, independently, in duplicate) and any processes for obtaining and confirming data from investigators. | 6 |
| Data items | 11 | List and define all variables for which data were sought (e.g., PICOS, funding sources) and any assumptions and simplifications made. | 6 |
| Risk of bias in individual studies | 12 | Describe methods used for assessing risk of bias of individual studies (including specification of whether this was done at the study or outcome level), and how this information is to be used in any data synthesis. | 7-8 |
| Summary measures | 13 | State the principal summary measures (e.g., risk ratio, difference in means). | 7 |
| Synthesis of results | 14 | Describe the methods of handling data and combining results of studies, if done, including measures of consistency (e.g., I^2^) for each meta-analysis. | 7 |
| Risk of bias across studies | 15 | Specify any assessment of risk of bias that may affect the cumulative evidence (e.g., publication bias, selective reporting within studies). | 7-8 |
| Additional analyses | 16 | Describe methods of additional analyses (e.g., sensitivity or subgroup analyses, meta-regression), if done, indicating which were pre-specified. | 7-8 |
| **RESULTS** | | |  |
| Study selection | 17 | Give numbers of studies screened, assessed for eligibility, and included in the review, with reasons for exclusions at each stage, ideally with a flow diagram. | 8 |
| Study characteristics | 18 | For each study, present characteristics for which data were extracted (e.g., study size, PICOS, follow-up period) and provide the citations. | 8-9 |
| Risk of bias within studies | 19 | Present data on risk of bias of each study and, if available, any outcome level assessment (see item 12). | 8-9 |
| Results of individual studies | 20 | For all outcomes considered (benefits or harms), present, for each study: (a) simple summary data for each intervention group (b) effect estimates and confidence intervals, ideally with a forest plot. | 9-11 |
| Synthesis of results | 21 | Present results of each meta-analysis done, including confidence intervals and measures of consistency. | 9-11 |
| Risk of bias across studies | 22 | Present results of any assessment of risk of bias across studies (see Item 15). | 11-13 |
| Additional analysis | 23 | Give results of additional analyses, if done (e.g., sensitivity or subgroup analyses, meta-regression [see Item 16]). | 11-13 |
| **DISCUSSION** | | |  |
| Summary of evidence | 24 | Summarize the main findings including the strength of evidence for each main outcome; consider their relevance to key groups (e.g., healthcare providers, users, and policy makers). | 13-14 |
| Limitations | 25 | Discuss limitations at study and outcome level (e.g., risk of bias), and at review-level (e.g., incomplete retrieval of identified research, reporting bias). | 21-22 |
| Conclusions | 26 | Provide a general interpretation of the results in the context of other evidence, and implications for future research. | 22-23 |
| **FUNDING** | | |  |
| Funding | 27 | Describe sources of funding for the systematic review and other support (e.g., supply of data); role of funders for the systematic review. | 24 |

From: Moher D, Liberati A, Tetzlaff J, Altman DG, The PRISMA Group (2009). Preferred Reporting Items for Systematic Reviews and Meta-Analyses: The PRISMA Statement. PLoS Med 6(7): e1000097. doi:10.1371/journal.pmed1000097

**Supplementary 2: List of terms of the search strategy**

Our search strategy was as follows: (LT OR OLT OR liver transplantation OR Grafting, Liver OR Liver Grafting OR Transplantation, Liver OR Liver Transplantations OR Liver Transplant OR Liver Transplants OR Transplant, Liver OR Hepatic Transplantation OR Hepatic Transplantations OR Transplantation, Hepatic) AND (AKI OR acute kidney injury OR Acute Kidney Injuries OR Kidney Injuries, Acute OR Kidney Injury, Acute OR Acute Renal Injury OR Acute Renal Injuries OR Renal Injuries, Acute OR Renal Injury, Acute OR Renal Insufficiency, Acute OR Acute Renal Insufficiencies OR Acute Renal Insufficiencies OR Acute Renal Insufficiencies OR Acute Renal Insufficiencies OR Acute Renal Insufficiencies OR Acute Renal Insufficiencies OR Renal Insufficiencies, Acute OR Renal Insufficiencies, Acute OR Acute Renal Insufficiency OR Kidney Insufficiency, Acute OR Acute Kidney Insufficiencies OR Kidney Insufficiencies, Acute OR Acute Kidney Insufficiency OR Kidney Failure, Acute OR Acute Kidney Failures OR Kidney Failures, Acute OR Acute Renal Failure OR Acute Renal Failures OR Renal Failures, Acute OR Renal Failure, Acute OR Acute Kidney Failure).

**Supplementary 3: Duplicate data identification and elimination (by title, author, cohort source, patient recruitment time, donor type and surgical technique)**

| Author | Study | Sample size | NOS | Cohort source | Patient recruitment time | Donor type and surgical technique | Decision |
| --- | --- | --- | --- | --- | --- | --- | --- |
| Cabezuelo, J. B. | Cabezuelo 2003 [[1](#_ENREF_1)] | 57/127 | 5 | University Hospital V. Arrixaca | 1991 - 1997 | OLT | Excluded |
|  | Cabezuelo 2006 [[2](#_ENREF_2)] | 57/127 | 7 | Arrixaca University Hospital | 1991 - 1997 | OLT | Included |
| Hilmi, Ibtesam A. | Hilmi 2015 (1) [[3](#_ENREF_3)] | 221/203 | 7 | University of Pittsburgh Medical Center | January 2005 - December 2009 | Cadaveric LT | Included |
|  | Hilmi 2015 (2) [[4](#_ENREF_4)] | 58/114 | 7 | University of Pittsburgh Medical Center | January 2006 - December 2011 | LDLT | Included |
| Jun, In-Gu | Jun 2016 [[5](#_ENREF_5)] | 999/618 | 7 | Asan Medical Center | November 2008 - December 2014 | LDLT | Excluded |
|  | Jun 2018 [[6](#_ENREF_6)] | 1270/595 | 7 | Asan Medical Center | January 2008 - October 2015 | LDLT | Included |
| Kalisvaart, Marit | Kalisvaart 2017 [[7](#_ENREF_7)] | 61/94 | 7 | Erasmus MC University Medical Center | July 2008 - October 2014 | DBD-LT | Included |
|  | Kalisvaart 2018 [[8](#_ENREF_8)] | 239/129 | 7 | Queen Elizabeth Hospital, and Erasmus MC University Medical Center | July 2008 - July 2016 | DCD-OLT | Included |
| Kim, Won Ho | Kim 2018 [[9](#_ENREF_9)] | 205/378 | 7 | Seoul National University Hospital | 2004 - 2015 | LDLT | Included |
|  | Kim 2019 [[10](#_ENREF_10)] | 265/265 | 7 | Seoul National University Hospital | November 2004 - December 2015 | LDLT | Excluded |
| Leithead, Joanna A. | Leithead 2012 [[11](#_ENREF_11)] | 47/41 | 7 | Queen Elizabeth Hospital | January 2007 - March 2011 | DCD-LT | Included |
|  | Leithead 2013 [[12](#_ENREF_12)] | 107/191 | 7 | Queen Elizabeth Hospital | January 2007 - June 2011 | DBD-LT | Included |
|  | Leithead 2014 [[13](#_ENREF_13)] | 72/72 | 7 | Queen Elizabeth Hospital | January 2007 - March 2011 | SLT (right extended lobe)-DBD | Included |
|  | Leithead 2014 [[14](#_ENREF_14)] | 381/771 | 7 | Queen Elizabeth Hospital | January 2000 - December 2011 | Higher risk grafts LT | Included |

**NOTE：**NOS, Newcastle-Ottawa Scale; OLT, orthotopic liver transplantation; LT, liver transplantation; LDLT, living donor live transplantation; DBD, donation after brain death; DCD, donation after cardiac death; SLT, split liver transplantation.

**Reference:**

1. Cabezuelo JB, Ramirez P, Acosta F, Torres D, Sansano T, Pons JA, Bru M, Montoya M, Rios A, Sánchez Bueno F *et al*: **Does the standard vs piggyback surgical technique affect the development of early acute renal failure after orthotopic liver transplantation?** *Transplant Proc* 2003, **35**(5):1913-1914.

2. Cabezuelo JB, Ramírez P, Ríos A, Acosta F, Torres D, Sansano T, Pons JA, Bru M, Montoya M, Bueno FS *et al*: **Risk factors of acute renal failure after liver transplantation**. *Kidney Int* 2006, **69**(6):1073-1080.

3. Hilmi IA, Damian D, Al-Khafaji A, Planinsic R, Boucek C, Sakai T, Chang CCH, Kellum JA: **Acute kidney injury following orthotopic liver transplantation: incidence, risk factors, and effects on patient and graft outcomes**. *Br J Anaesth* 2015, **114**(6):919-926.

4. Hilmi IA, Damian D, Al-Khafaji A, Sakai T, Donaldson J, Winger DG, Kellum JA: **Acute kidney injury after orthotopic liver transplantation using living donor versus deceased donor grafts: A propensity score-matched analysis**. *Liver Transpl* 2015, **21**(9):1179-1185.

5. Inoue Y, Soyama A, Takatsuki M, Hidaka M, Kinoshita A, Natsuda K, Baimakhanov Z, Kugiyama T, Adachi T, Kitasato A *et al*: **Does the development of chronic kidney disease and acute kidney injury affect the prognosis after living donor liver transplantation?** *Clin Transplant* 2016, **30**(5):518-527.

6. Jun I-G, Kwon H-M, Jung K-W, Moon Y-J, Shin W-J, Song J-G, Hwang G-S: **The Impact of Postreperfusion Syndrome on Acute Kidney Injury in Living Donor Liver Transplantation: A Propensity Score Analysis**. *Anesth Analg* 2018, **127**(2):369-378.

7. Kalisvaart M, de Haan JE, Hesselink DA, Polak WG, Hansen BE, Ijzermans JNM, Gommers D, Metselaar HJ, de Jonge J: **The postreperfusion syndrome is associated with acute kidney injury following donation after brain death liver transplantation**. *Transpl Int* 2017, **30**(7):660-669.

8. Kalisvaart M, Schlegel A, Umbro I, de Haan JE, Scalera I, Polak WG, Ijzermans JNM, Mirza DF, Perera MTPR, Isaac JI *et al*: **The Impact of Combined Warm Ischemia Time on Development of Acute Kidney Injury in Donation After Circulatory Death Liver Transplantation: Stay Within the Golden Hour**. *Transplantation* 2018, **102**(5):783-793.

9. Kim WH, Lee H-C, Lim L, Ryu H-G, Jung C-W: **Intraoperative Oliguria with Decreased SvO₂ Predicts Acute Kidney Injury after Living Donor Liver Transplantation**. *J Clin Med* 2018, **8**(1):29.

10. Kim WH, Oh H-W, Yang S-M, Yu JH, Lee H-C, Jung C-W, Suh K-S, Lee KH: **Intraoperative Hemodynamic Parameters and Acute Kidney Injury After Living Donor Liver Transplantation**. *Transplantation* 2019, **103**(9):1877-1886.

11. Leithead JA, Tariciotti L, Gunson B, Holt A, Isaac J, Mirza DF, Bramhall S, Ferguson JW, Muiesan P: **Donation after cardiac death liver transplant recipients have an increased frequency of acute kidney injury**. *Am J Transplant* 2012, **12**(4):965-975.

12. Leithead JA, Armstrong MJ, Corbett C, Andrew M, Kothari C, Gunson BK, Muiesan P, Ferguson JW: **Hepatic ischemia reperfusion injury is associated with acute kidney injury following donation after brain death liver transplantation**. *Transpl Int* 2013, **26**(11):1116-1125.

13. Leithead JA, Armstrong MJ, Corbett C, Andrew M, Kothari C, Gunson BK, Mirza D, Muiesan P, Ferguson JW: **Split liver transplant recipients do not have an increased frequency of acute kidney injury**. *Transpl Int* 2014, **27**(11):1125-1134.

14. Leithead JA, Rajoriya N, Gunson BK, Muiesan P, Ferguson JW: **The evolving use of higher risk grafts is associated with an increased incidence of acute kidney injury after liver transplantation**. *J Hepatol* 2014, **60**(6):1180-1186.

**Supplementary 4: Characteristics of included studies**

| Name of first author;  Year；  Country；  Cohort source | Definition of AKI;  Diagnosis criteria;  Duration of evaluation | No. of sample/cases;  Study design;  Statistic method;  Procedure;  Incidence of AKI | Adjusting variable | Identified risk factors | OR(95% CI) |
| --- | --- | --- | --- | --- | --- |
| Gabriel Contras; 2001 [[1](#_ENREF_1)]；  USA；  Jackson Memorial Hospital | Early RRT；  The institution of ultrafiltration, dialysis, and/or hemodiafiltration；  within 1 week after OLT | 28/232;  Retrospective study;  Multivariate logistic regression with forward stepwise model;  OLT;  Early RRT: 28/260 (10.8%) | MAP, Blood products, AST,  White race | Preoperative BUN (per 10 mg/dl) | 1.52 (1.15 - 2.01) |
|  |  |  |  | Preoperative Scr (per 1 mg/dl) | 1.91 (1.06 - 3.44) |
|  |  |  |  | Preoperative urine output (per L/d) | 0.12 (0.03 - 0.44) |
| Lima, Emerson Q.;  2003 [[2](#_ENREF_2)];  Brazil;  University of Sao Paulo School of Medicine | ARF;  Scr ≥ 2.0 mg/dL;  Within 30 days after LT | 56/36;  Retrospective study;  Multivariate logistic regression;  LT;  ARF: 56/92 (60.9%) | Preoperative serum urea, Preoperative serum potassium, Anesthesia time, Postoperative cyclosporine use, Postoperative serum bilirubin, Postoperative Scr | Preoperative Scr > 1.0 mg/dL | 9.24 (1.8 - 47.6) |
|  |  |  |  | Intraoperative blood transfusions > 5 units | 5.17 (1.12 - 23.9) |
|  |  |  |  | Postoperative hypotension | 7.14 (1.15 - 41.6) |
|  |  |  |  | Intraoperative hypotension | 3.85 (1.05 - 13.7) |
| Lebrón Gallardo, Miguel.;  2004 [[3](#_ENREF_3)];  Spain;  UCI Hospital Carlos Haya | ED;  Scr > 1.5 mg/dL or, in patients with pretransplant RD, Scr level > 2 that of pretransplant;  Within 3 months after LT | 158/87;  Retrospective study;  Multivariate logistic regression with gradual step-by-step withdrawal of variables;  OLT;  ED: 158/245 (64.1%)  RRT：28/245 (11.4%) CVVHF: 26/245 (10.6%) Hemodialysis: 2/245 (0.8%) | Age，MELD，FFP，Lowest postoperative prothombin，Highest bilirubin postoperative，Intraoperative fibrinolysis，Reperfusion syndrome，Postoperative hypotension | Preoperative Scr | 25.40 (6.70 - 96.20) |
|  |  |  |  | Homologous packed erythrocytes | 1.30 (1.10 - 1.50) |
|  |  |  |  | Higher APACHE II score at ICU admission | 1.10 (1.01 - 1.20) |
|  |  |  |  | Infection | 2.80 (1.30 - 6.40) |
| Sanchez,Edmund Q.;  2004 [[4](#_ENREF_4)];  USA;  BaylorUniversity Medical Center | RRT;  Either conventional intermittent hemodialysis or CRRT (continuous venovenous hemodialysis or  continuous venovenous hemodiafiltration);  NA | 87/637;  Retrospective study;  Multivariate logistic regression;  OLT;  RRT: 87/724 (12.0%)  Hemodialysis: 17/724 (2.3%)  CRRT: 70 (9.7%) | Preoperative hemoglobin, Preoperative total bilirubin,  Waiting list time, UNOS status 1 or 2A, Retransplant, Length of anesthesia, Length of bypass, Intraoperative RBC, Intraoperative FFP, Urinary output, Postoperative RBC, Postoperative FFP | Preoperative Scr (>1.9 mg/dL) | 3.57 (1.65 - 7.72) |
|  |  |  |  | MELD score (>21) | 2.50 (1.27 - 4.92) |
|  |  |  |  | BUN (>27 mg/dL) | 2.68 (1.30 - 5.54) |
|  |  |  |  | Postoperative ICU day (> 3) | 10.23 (5.60 -18.66) |
| Cabezuelo, J. B.;  2006 [[5](#_ENREF_5)];  Spain;  Arrixaca University Hospital | ARF;  A persistent rise of 50% increase or more of the Scr;  Early-ARF: within 1 week after LT | Early-ARF: 57/127;  Retrospective study;  Multivariate logistic regression;  OLT;  Early-ARF: 57/184 (30.9%); | UNOS status, Preoperative serum bilirubin, Urgent retransplant, Child-Pugh score, Urinary alterations, Intraoperative FFP transfusion, Intraoperative cryoprecipitate transfusion, Intraoperative complications, Intraoperative noradrenaline use, Intraoperative dobutamine use, Surgery type, Postoperative mechanical ventilation, Postoperative platelets transfusion, Postoperative bacterial infection, Postoperative acute rejection | Preoperative serum albumin < 3.2 g/dl (Early-ARF) | 0.30 (0.20 - 0.40) |
|  |  |  |  | Preoperative ARF(Early-ARF) | 10.00 (1.30 - 78.00) |
|  |  |  |  | Postoperative dopamine use (Early-ARF) | 1.60 (1.30 - 2.10) |
|  |  |  |  | Graft dysfunction II–IV vs I  (Early-ARF) | 5.60 (1.80 - 17.00) |
| Guitard, J.;  2006 [[6](#_ENREF_6)];  France;  CHU Rangueil | ARI/ARF;  RIFLE criteria；  Within the 1 month of LT | ARI:39/55 (ARF: 21/73);  Retrospective study;  Multivariate logistic regression with step-by-step analysis;  Cadaveric LT;  ARI: 39/94 (41.5%);  ARF：21/94 (22.3%)  Dialysis: 10 /94 (10.6%) | Requirement for blood transfusions during OLT, Time to AST peak and ALT peak, Mean arterial blood pressure at 6 hours post-OLT, Occurrence of severe infection post-OLT, Level of Scr at post-OLT Days 1,3, 5, 7, 10 and 15 | Pre-OLT BMI (>24) (ARI) | 8.62 (2.24 - 33.00) |
|  |  |  |  | Need for vasopressive drugs (ARI) | 41.50 (5.30-322.70) |
|  |  |  |  | CNIs, dosage at day7 (< 4/0.1 mg/kg/d) (ARI) | 3.46 (1.14 - 10.40) |
| Wei, Y.;  2006 [[7](#_ENREF_7)];  China;  West China Hospital, Sichuan University | ARF;  Scr > 1.5 mg/dL (135 mol/L) among patients with normal preoperative renal function or a 50% increase in Scr for those subjects with preexistent renal impairment before transplantation;  Within 30 days after LT | 25/64;  Retrospective study;  multivariate logistic regression;  OLT;  ARF: 25/89 (28.1%) | Sex, Causes, Child-Pugh score, Venovenous bypass, Operation modality, Age, Preoperative tuberculosis, Ascites, Intraoperative blood transfusion volume, Intraoperative urine volume, Preoperative Scr | Duration of operation | 1.55 (1.14 - 2.10) |
| O'Riordan, A.;  2007 [[8](#_ENREF_8)];  Ireland;  Irish National liver transplant unit | ARI/ARF;  RIFLE criteria;  Within 2 weeks after LT | ARI: 39/221;  ARF: 90/221;  Retrospective study;  Multivariate logistic regression;  OLT;  ARI: 39/350 (11.1%);  ARF: 90/350 (25.7%) | ARI: Pre-OLT Mayo end-stage liver disease score, Intra-operative blood loss, Red cell concentrate volume;  ARF: Pre-OLT hypertension, Pre-OLT Mayo end-stage liver disease score, Indications for OLT, Hepatitis C virus, Fulminant hepatic failure, Primary biliary cirrhosis, Intraoperative blood loss | Pre-OLT hypertension (ARI) | 8.00 (1.30 - 50.70) |
|  |  |  |  | Alcoholic liver disease (ARI) | 5.00 (1.10 - 22.30) |
|  |  |  |  | Pre-OLT creatinine (ARF) | 3.60 (1.80 - 7.20) |
|  |  |  |  | Intraoperative use of inotrope (ARF) | 2.90 (1.40 - 5.90) |
|  |  |  |  | Postoperative aminoglycoside use (ARF) | 2.50 (1.20 - 5.10) |
| Shu, Ming;  2007 [[9](#_ENREF_9)];  China;  Ruijin Hospital | ARF;  A postoperative Scr level of more than 130 µmol/L or an increase in Scr of greater than 100 µmol/L;  Within 72 hours after surgery | 14/48;  Retrospective study;  Multivariate logistic regression with forward stepwise variable selection;  OLT;  14/62 (22.6%) | Age (> 60 y), Postoperative sepsis, Postoperative relaparotomy, Postoperative respiratory failure, Diuretics | Intraoperative blood transfusion volume of more than 15 U | 15.75 (1.04 - 237.11) |
|  |  |  |  | Intraoperative MAP | 1.15 (1.01 - 1.30) |
|  |  |  |  | Postoperative intra-abdominal hypertension (IAH) | 18.36 (1.72 - 195.80) |
| Iglesias, Jose I.;  2010 [[10](#_ENREF_10)];  USA;  NIDDK-LTD:  three participating clinical centers including Mayo Clinic;  University of Nebraska Medical Center; University of California | AKI;  AKIN criteria;  Within 48 hours | 243/445;  Retrospective cohort study;  Multivariate logistic regression with forward variable selection;  OLT;  AKI: 243/668 (35%) | Sex, Race, BSA, Hepatic disease leading to transplant, Porto-systemic encephalopathy, Ascites, Preoperative platelets, Preoperative PT, Preoperative sodium, Preoperative total bilirubin, Preoperative albumin, intraoperative blood transfusion, Venovenous bypass, Postoperative loop diuretic use, Postoperative cyclosporin use, Postoperative tacrolimus use | BMI | 1.09 (1.05 -1.13) |
|  |  |  |  | CTP score | 1.31 (1.15 - 1.49) |
|  |  |  |  | Urine output (cross-clamp) | 0.99 (0.990 - 0.996) |
|  |  |  |  | ΔSCr/Δt (mg/dL/day) | 0.99 (0.97-1.00) |
|  |  |  |  | Stroke volume (postoperative) | 1.006 (1.001-1.011) |
|  |  |  |  | Race (non-Caucasian) | 1.93 (1.18-3.10） |
|  |  |  |  | Tacrolimus (postoperative) | 1.93 (1.10 -3.40） |
| Kundakci, A.;  2010 [[11](#_ENREF_11)]；  Turkey;  The Baskent University Hospital | AKD;  RIFLE criteria;  Within 7 days after LT | 64/48;  Retrospective study;  Multivariate logistic regression;  OLT;  AKD: 64/112 (57%) with risk, injury, and failure frequencies of 19%, 11%, and 28%, respectively. | Intraoperative inotropic agents requirement, Received more intraoperative colloid, Longer durations of anesthesia | MELD score | 1.11 (1.02-1.20) |
|  |  |  |  | Duration of anhepatic phase | 1.02 (1.00-1.04) |
|  |  |  |  | Intraoperative acidosis | 0.28 (0.09-0.83) |
| Sakai, Tetsuro;  2010 [[12](#_ENREF_12)];  USA;  University of Pittsburgh Medical Center | ARF;  RIFLE criteria;  NA | 95/314;  Retrospective study;  Primary deceased donor LT;  Pearson’s chi-square test or Fisher’s exact test;  ARF: 95/409 (23.2%) | - (Univariate analysis) | Surgical methods (Piggyback technique without venovenous bypass /Retrohepatic caval resection technique with venovenous bypass) | 0.34 (0.19 - 0.62) |
|  |  |  |  | Surgical methods (Piggyback technique without venovenous bypass / Piggyback technique with venovenous bypass) | 0.55 (0.31 - 0.97) |
| Xu, Xiao;  2010 [[13](#_ENREF_13)];  China;  The First Affiliated Hospital, Zhejiang University School of Medicine | AKI;  Scr >1.5 mg/dL with an increase of 50% above the baseline level or/and the presence of need for RRT;  Within 1 week after LT | 33/69;  Retrospective cohort study;  Multivariate logistic regression with forward stepwise;  OLT;  AKI: 33/102 (32.4%);  RRT: 10 (9.8%) | Age, MELD score, Blood urea nitrogen, Serum sodium, Serum potassium, Red blood cells, Fresh frozen plasma, Platelets, Blood loss, Adrenaline | Preoperative Scr > 1.2 mg/dl | 3.03 (0.90 - 10.25) |
|  |  |  |  | Intraoperative urine output ≤ 60 ml/h | 9.42 (2.93 - 30.28) |
|  |  |  |  | Intraoperative hypotension status | 4.67 (1.34 - 16.36) |
|  |  |  |  | Intraoperative use of noradrenaline | 0.09 (0.01- 0.55) |
| Zhu, M;  2010 [[14](#_ENREF_14)];  China;  Renji Hospital. | AKI;  AKIN criteria;  Within 28 days after LT | AKI:116/77;  Stage 1 and 2 AKI: 83/77;  Stage 3 AKI: 33/77;  Retrospective study ;  Multivariate logistic regression;  OLT;  AKI: 116/193(60.1%), namely, stages 1, 2, and 3 in 30%, 13% and 17.1% respectively | AKI: Age, DM, Hypertension, Preoperative Scr, APACHE Ⅱ, Intraoperative red cell concentrate, Anesthesia, Operative time | MELD (per score; AKI) | 1.05 (1.01 - 1.09) |
|  |  |  |  | Gender (Male/Female) | 2.39 (1.01 - 5.65) |
| Chen, Jie;  2011 [[15](#_ENREF_15)];  USA;  University of California, Los Angeles Medical Center | ARI/ARF;  RIFLE criteria;  Within 1 week after LT | 118/118;  A Matched Study;  Multivariate logistic regression;  118 ARI or ARF patients were matched with patients without post-LT renal injury by gender, creatinine, and BMI;  LT;  ARI and ARF:118/334 (38.4%);  ARI: 63/334 (20.4%);  ARF: 55/334 (18.0%) | Gender, Scr, and BMI | RBC transfusion | (application of dummy variable) |
|  |  |  |  | 0 - 5 units | 1 (reference) |
|  |  |  |  | 6 - 9 units | 2.66 (1.04 - 6.85) |
|  |  |  |  | 10 - 17 units | 7.06 (2.74 - 18.19) |
|  |  |  |  | ≥ 18 units | 8.84 (3.24 - 24.10) |
|  |  |  |  | Large dose of vasopressors | 2.24 (1.09 - 4.61) |
|  |  |  |  | Hypoalbuminemia (< 3.5 mmol/L) | 2.79 (1.43 - 5.43) |
| Leithead, J. A.;  2012 [[16](#_ENREF_16)];  UK;  Queen Elizabeth Hospital | AKI;  RIFLE criteria;  Following transplantation but prior to hospital discharge | 47/41;  Retrospective study;  Nonparsimonious multiple logistic regression with propensity risk score matching method;  DCD-LT;  AKI: 47/88 (53.4%) | Age, Gender, Ethnicity, Diagnosis, BMI, DM, Hypertension, Ascites, eGFR, MELD, Waiting list time | Higher peak AST during the immediate postoperative period | 7.44 (2.78 - 19.88) |
| Leithead, J. A.;  2013 [[17](#_ENREF_17)];  UK;  Queen Elizabeth Hospital | AKI;  RIFLE criteria;  Following transplantation but prior to hospital discharge | 107/191;  Retrospective study ;  Multivariate logistic regression;  DBD-LT;  AKI: 107/298 (35.9%) | Age, Female gender, Ethnicity, Hepatitis C, Pretransplant DM, Hypertension, eGFR (ml/min/1.73 m^2^), MELD score, Renal sparing immunosuppression | Refractory ascites | 2.75 (1.18 - 6.39) |
|  |  |  |  | Intraoperative RBC transfusion (U) | (application of dummy variable) |
|  |  |  |  | 0 | 1 (reference) |
|  |  |  |  | 1 - 4 | 1.68 (0.81 - 3.49) |
|  |  |  |  | ≥ 5 | 3.57 (1.47 - 8.67) |
|  |  |  |  | Intraoperative use of inotropes | 2.88 (1.31 - 6.33) |
|  |  |  |  | Postoperative peak AST (U/L) | (application of dummy variable) |
|  |  |  |  | < 1500 | 1 (reference) |
|  |  |  |  | 1500 - 2999 | 2.02 (1.04 - 3.94) |
|  |  |  |  | ≥ 3000 | 8.02 (3.28 - 19.64) |
|  |  |  |  | Sepsis | 2.86 (1.34 - 6.13) |
| Liu, Shuang;  2013 [[18](#_ENREF_18)];  China;  Shanghai First People’s Hospital | ARF;  RIFLE criteria;  Within their  hospital stay period | 43/346;  Retrospective study ;  Multivariate logistic regression;  OLT;  ARF: 43/389 (11.1%) | Age, Etiology, Child-Pugh grade, ABO blood group, Blood loss, Infections, Cryoprecipitate, Steroid | Cryoprecipitate (per U) | 1.1 (1.0 - 1.1) |
|  |  |  |  | ABO blood group compatible | 0.33 (0.15 - 0.73) |
|  |  |  |  | Infections before ARF | 4.0 (1.8 - 8.9) |
|  |  |  |  | Blood loss (per 500 ml) | 1.1 (1.0 - 1.1) |
| Romano, Thiago Gomes;  2013 [[19](#_ENREF_19)];  Brazil;  Hospital Sirio Libanes | AKI;  An increase equal or greater than 0.3 mg/dL in Scr;  Within 72 hours after LT | 52/40;  Retrospective study;  Multivariate logistic regression with backward stepwise variable selection;  OLT;  AKI: 52 /92 (56.5%) | Preoperative bilirubin, Preoperative INR, In-hospital days prior surgery, Infection prior surgery, MELD of indication, Use of terlipressin during surgery, Volume of blood components during surgery, Noradrenaline dose of admission in ICU | Calculated MELD | 1.03 (1.00 - 1.06) |
| Utsumi, Masashi;  2013 [[20](#_ENREF_20)];  Japan;  Okayama University Hospital | ARI;  RIFLE criteria;  Within 28 days after LT | 126/74;  Retrospective study;  Multivariate logistic regression;  LDLT;  ARI: 121/200 (60.5%);  R-class: 47/200 (23.5%);  I-class: 42/200 (21%);  F-class: 32/200 (16%) | Recipient age, Sex, Background disease, Hypertension, Donor age, Right/left lobe graft, Operative time, Cold ischemic time, Warm ischemic time, Immunosuppressive induction of CNI, Combined use of mycophenolate mofetil | MELD ≥ 20 | 2.96 (1.19 - 7.63) |
|  |  |  |  | Small-for-size graft (GW/RBW < 0.7%) | 3.10 (1.04 - 9.79) |
|  |  |  |  | Blood loss/body weight > 55 ml/kg | 3.70 (1.53 - 9.53) |
|  |  |  |  | Overexposure to calcineurin inhibitor | 2.59 (1.14 - 6.11) |
|  |  |  |  | Preoperative DM | 3.23 (1.02 - 10.7) |
| Karapanagiotou, A.; 2014 [[21](#_ENREF_21)];  Greece;  Hippokration General Hospital | AKI;  RIFLE criteria;  Within 7 days after LT | 23/48;  Retrospective study;  Chi-square test;  OLT with piggyback technique;  AKI: 23/71 (32.4%) | - (Univariate analysis) | Vasoactive support | 11.88 (3.52 - 40.14) |
|  |  |  |  | Infection | 3.60 (1.22 - 10.62) |
| Klaus, F.;  2014 [[22](#_ENREF_22)];  Brazil;  Irmandade Santa Casa de Misericórdia de Porto Alegre | AKI;  An increase ≥ 50% from preoperative baseline Scr levels;  During the hospitalization period. | 64/49;  Retrospective cohort study;  Multivariate logistic regression with backward variable selection;  Deceased-organ donor LT with piggyback method;  AKI: 64/113 (6.6%);  RRT: 22/113 (19.5%) | Preoperative Scr, Preoperative hemoglobin, Preoperative albumin, Preoperative total bilirubin，Preoperative INR，Proteinuria | MELD score | 1.12 (1.02 - 1.23) |
|  |  |  |  | Diuretic use | 3.78 (1.72 - 8.30) |
| Leithead, Joanna A;  2014 [[23](#_ENREF_23)]；  UK;  Queen Elizabeth Hospital | AKI;  KDIGO criteria;  Following transplantation but prior to hospital discharge | 72/72;  Retrospective single-centre study;  Backward sequential logistic regression with propensity risk score matching method;  SLT (right extended lobe)-DBD;  Before PRS matching:  AKI in SLT: 33/76 (43.4%); AKI in FSLT: 168/301 (55.8%);  After PRS matching:  AKI in SLT: 29/ 72 (40.3%); AKI in FSLT: 34/72 (47.2%) | Age, Pretransplant eGFR,  Pretransplant MELD | Pretransplant refractory ascites/No refractory ascites | 3.96 (1.15-13.69) |
|  |  |  |  | Intraoperative RBC transfusion(≥ 5 units /0 - 4 units) | 5.01 (1.63 - 15.40) |
|  |  |  |  | Peak postoperative AST (per u/l) | 2.11 (1.25 - 3.54) |
| Leithead, Joanna A;  2014 [[24](#_ENREF_24)];  UK;  Queen Elizabeth Hospital | AKI;  KDIGO criteria;  Within 7 days after LT | 381/771;  Single-center study;  Multivariate logistic regression;  Higher risk grafts first-single-organ LT for chronic liver disease;  AKI: 381/1152 (33.1%) | Age, Gender, Hepatitis C infection, Non-alcoholic fatty liver disease, Diabetes mellitus, eGFR, Hyponatraemia | Increasing recipient warm ischaemic time | 1.02 (1.00 - 1.03) |
|  |  |  |  | DCD transplantation | 2.67 (1.71- 4.18) |
|  |  |  |  | Donor age ≥ 60 years | 1.49 (1.07 - 2.09) |
|  |  |  |  | Donor BMI ≥ 30 kg/m^2^ | 1.99 (1.40 - 2.84) |
| Nadeem, Ashraf;  2014 [[25](#_ENREF_25)];  Saudi Arabia;  King Faisal Specialist Hospital and Research Centre | AKI;  RIFLE criteria;  Within 3 days after LT | 57/101;  Observational study;  Multivariate logistic regression with backward variable selection ;  104/158 (65.8%) LDLT, 53 /158 (33.5%) cadaveric LT and 1/158 (0.6%) retransplant  AKI: 58/158 (36.4%);  R-class: 30/158 (52%);  I-class: 12/158 (21%);  F-class: 16/158 (27%) | APACHE Ⅱ score at admission to ICU, Colloids ≥ 1,500 ml received in operating room, Vasopressors requirement at 2 days posttransplant, Serum chloride level at day 2 | Infusion of greater than 3,200 ml of chloride-liberal fluids | 6.25 (2.69 - 14.5) |
|  |  |  |  | Preoperative MELD score | 1.08 (1.02 - 1.15) |
| Papadopoulos, S.;  2014 [[26](#_ENREF_26)];  Greece;  “Hippokratio” Hospital | AKI;  AKIN criteria;  Within 7 days after LT | 16/55 (Stratified by RRT)  Retrospective study;  Multivariate analysis;  LT;  AKI: 37/71(52.1%);  (stage I, 22.5%; stage II, 7.0%; and stage III, 22.5%) | NA | Transfusion of >10 units of RBCs | 1.15 (1.80 - 7.53) |
|  |  |  |  | MELD score > 23 (univariate) | 5.10 (1.47 - 17.70) |
|  |  |  |  | Hemodynamic instability (univariate) | 6.30 (3.35 - 7.90) |
| Sirivatanauksorn, Y.;  2014 [[27](#_ENREF_27)];  Thailand;  Siriraj Hospital | ARF;  An increased in Scr level more than 1.5 times compared with the preoperative level;  Within 1 week after LT | 58/23;  Retrospective study;  Multivariate logistic regression;  OLT;  58/81 (71.6%) | Preoperative prothrombin time longer than 15 seconds, Intraoperative blood loss more than 2500 mL, Requirement of more than 1000 mL blood | Intraoperative hypotension > 30 min | 3.84 (1.11 - 13.30) |
|  |  |  |  | Presence of postoperative hypotension | 5.44 (1.12 - 26.48) |
| Smoter, P.;  2014 [[28](#_ENREF_28)];  Poland;  Medical University of Warsaw | ARF;  An eGRF < 60 mL/min/1.73 m^2^;  Within 7 days after LT | 46/52;  Retrospective case-control study;  Multivariate logistic regression;  Cadaveric donor OLT;  ARF: 46/98 (46.9%) | NA | Intraoperative transfusion < 6 units of blood (univariate) | 0.19 (0.06 - 0.60) |
|  |  |  |  | Transfusion < 6 units of blood (univariate) | 0.35 (0.13 - 0.93) |
|  |  |  |  | High-dose tacrolimus immunosuppression (univariate) | 2.04 (6.22 - 18.11) |
|  |  |  |  | Intraoperative creatinine > 0.9mg/dL | 0.42 (0.05 - 0.95) |
|  |  |  |  | Creatinine 24 hours post-transplantation > 0.9 mg/dL | 6.39 (1.52 - 26.91) |
|  |  |  |  | Urea 24 hours post-transplantation > 40mg/dL | 45.00 (7.19 - 370.56) |
| J.M. Kim;  2014 [[29](#_ENREF_29)];  Korea;  Severance Hospital, Yonsei University Health System | ARF;  Postoperative CRRT application;  Within 1 week after LT | 42/115;  Retrospective study;  Multivariate logistic regression;  110 LDLT and 47 deceased donor LT;  CRRT: 42/157 (26.8%) | Preoperative hemoglobin, Preoperative hematocrit, Preoperative BUN, Preoperative total bilirubin, Preoperative INR, Intraoperative RBC transfusions, Length of ICU stay, Postoperative vasopressor use, Duration of the use of postoperative vasopressor, Ischemia reperfusion injury grade | Hepatic encephalopathy | 5.47 (1.93 - 15.52) |
|  |  |  |  | Deceased donor | 3.47 (1.23 - 9.80) |
|  |  |  |  | MELD score | 1.09 (1.03 - 1.15) |
|  |  |  |  | Intraoperative blood loss (L) | 1.16 (1.06 - 1.27) |
|  |  |  |  | Hepatocellular carcinoma | 0.11 (0.02 - 0.84) |
| Aksu Erdost, H.;  2015 [[30](#_ENREF_30)];  Turkey;  School of Medicine, Dokuz Eylul University | ARI;  RIFLE criteria;  By the 1st and 7th days of the surgery | 35/405;  Retrospective study;  Multivariate logistic regression;  LT;  ARI: 35/440 (7.95%) | Age, Sex | Preoperative hemoglobin (<9 mg/dL) | 2.84 (1.16 - 6.94) |
|  |  |  |  | Blood loss (mL) | 0.9996 (0.9994 - 0.9999) |
| Barreto, Adller G. C.;  2015 [[31](#_ENREF_31)];  Brazil;  Walter Cantidio University Hospital | AKI;  AKIN criteria;  Within 72 hours after LT | 64/70;  Retrospective cohort study;  Multivariate logistic regression with backward stepwise elimination algorithm;  OLT;  AKI: 64/134 (46.7%); Hemodialysis: 33/64 (51.5%) | Presence of encephalopathy preoperatively, Serum sodium | Viral etiology for underlying end stage liver disease | 2.90 (1.20 - 7.00) |
|  |  |  |  | Warm ischemia time | 1.10 (1.01 - 1.20) |
|  |  |  |  | Serum level of lactate | 1.30 (1.02 - 1.89) |
| Chen, Hsiu-Pin；  2015 [[32](#_ENREF_32)]；  China (Taiwan)；  Taiwanese National Health Insurance Research Database | ARF；  IDC-9-CM codes；  NA | 214/2648;  Retrospective, population-based, cohort study;  Multivariate logistic regression;  LT;  ARF: 214/3317 (6.5%) | Age, Sex, Pre-existing  hypertension | Cerebrovascular diseases | 2.06 (1.12 - 3.80) |
| Hand, William R.; 2015 [[33](#_ENREF_33)]；  USA；  Medical University of South Carolina | AKI；  RIFLE criteria;  Within 7 days of surgery | 57/60;  Retrospective cross-sectional study;  Multivariatelogistic regression and propensity score matching;  OLT;  AKI: 57/117 (48.7%)  RRT: 18/174 (10.3%) | Admission severity of illness; Mean intraoperative CVP (mm Hg); Intraoperative estimated blood loss (mL); Given intraoperative  platelets; Used cell saver  intraoperatively | Intraoperative colloidal use  (Received HES/received albumin) | 2.94 (1.13 - 7.70) |
| Hilmi, I. A.;  2015 [[34](#_ENREF_34)]；  USA;  University of Pittsburgh Medical Center | AKI;  A 50% increase in SCr from the baseline (preoperative value) or a 26.5 mmol/L increase from baseline within 48 h without urine output;  Within 72 h of surgery | 221/203;  Multivariate logistic regression with backward stepwise elimination algorithm;  Cadaveric LT;  AKI: 221/424 (52.1%) | Aetiology of end-stage liver disease, Intraoperative use of RBC, Intraoperative use of FFP, Intraoperative use of platelets | Female gender | 1.8 (1.18 - 2.88) |
|  |  |  |  | Weight > 100 kg | 2.3 (1.39 - 3.91) |
|  |  |  |  | CTP score | 1.4 (1.24 - 1.57) |
|  |  |  |  | Pre-existing DM | 1.9 (1.24 - 3.05) |
| Hilmi, I. A.;  2015 [[35](#_ENREF_35)]；  USA;  University of  Pittsburgh Medical Center | AKI;  A 50% increase  in Scr from baseline (preoperative) values within 48 hours;  Within 72 h of surgery | Before matching: 21/79,  After matching: 58/114;  Retrospective study;  Multivariate logistic regression with forward stepwise elimination algorithm, Propensity score matching,  Mixed effects logistic regression;  LDLT;  Before matching: AKI in LDLT: 21/100 (21.0%),  AKI in CDLT: 221/424 (52.1%);  After matching: AKI in LDLT: 20/86 (23.3%),  AKI in CDLT: 38/86 (44.2%); | MELD score, Use of VVBP, Number of platelet units | Severe postreperfusion syndrome (PRS) | 4.9 (1.3 - 17.2) |
|  |  |  | MELD score, Scr, Age, Gender, Pre-LT DM | Live donor liver graft (vs.  cadaveric) | 0.31 (0.10 - 0.98) |
| Mukhtar, Ahmed;  2015 [[36](#_ENREF_36)];  Egypt;  Wady El-Neel liver  transplant center | AKI;  AKIN criteria;  Within 96 hours after LT | 115/188;  Retrospective case-controlled study;  Multivariate logistic regression and propensity score matching;  LDLT;  AKI: 115/303 (38.0%);  Dialysis: 28/303 (9.2%) | Age, Sex, Reason for transplant, Duration of surgery, Ischemia time, Postoperative factors | GW/RBW < 0.8 | 2.65 (1.10 - 6.30) |
|  |  |  |  | Intraoperative RBC transfusion ≥ 10 units | 2.97 (1.70 - 4.90) |
|  |  |  |  | Intraoperative terlipressin therapy | 0.40 (0.20 - 0.80) |
|  |  |  |  | Intraoperative use of vasopressors | 2.10 (1.18 - 3.85) |
|  |  |  |  | MELD score ≥ 20 | 1.50 (0.80 - 2.60) |
|  |  |  |  | Preoperative DM | 1.10 (0.60 - 1.90) |
|  |  |  |  | Preoperative Scr | 0.75 (0.40 - 1.40) |
| Park, Mi Hye;  2015 [[37](#_ENREF_37)];  Korea;  Samsung Changwon Hospital | AKI;  RIFLE criteria;  Within one month after LDLT | 147/391;  Retrospective study;  Multivariate logistic regression with forward stepwise variable selection;  LDLT;  AKI: 147/538 (27.3%);  RRT: 34/538 (6.3%) | DM, Alcoholic liver cirrhosis, Child-Turcotte-Pugh score, Estimated GRWR, Cold ischemia time, Intraoperative RBC transfusion, Intraoperative FFP transfusion, Intraoperative platelet transfusion | BMI > 27.5 kg/m^2^ | 2.46 (1.32 - 4.55) |
|  |  |  |  | Serum albumin < 3.5 mg/dl | 1.76 (1.05 - 2.94) |
|  |  |  |  | MELD score > 20 | 2.01 (1.17 - 3.44) |
|  |  |  |  | Operation time > 600 min | 1.81 (1.07 - 3.06) |
|  |  |  |  | Warm ischemic time > 40 min | 2.61 (1.55 - 4.38） |
|  |  |  |  | Postreperfusion syndrome | 2.96 (1.55 - 4.38） |
|  |  |  |  | Mean blood glucose during the day of surgery >150 mg/dl | 1.66 (1.01 - 2.70） |
|  |  |  |  | Cryoprecipitate > 6 units | 4.96 (2.84 - 8.64） |
|  |  |  |  | Blood loss/body weight > 60 ml/kg | 4.05 (2.28 - 7.21） |
|  |  |  |  | CNI use without combined MMF | 1.87 (1.14 - 3.06） |
| Sang, Bo-Hyun; 2015 [[38](#_ENREF_38)];  Korea;  Asan Medical Center | AKI;  AKIN criteria;  On postoperative 1 to 7 days | 593/405;  Retrospective study;  Multivariate logistic regression;  LDLT;  AKI: 593/998 (59.4%) | Child-Turcotte-Pugh score,  Model for end-stage liver  disease score, Mean blood pressure, Hemoglobin, Platelet count, FFP transfusion, Platelet transfusion, Warm ischemic time, Graft-to-recipient weight ratio | Postoperative albumin level  < 3.0g/dL within 2 d | 0.42 (0.28 - 0.64) |
|  |  |  |  | BMI | 1.06 (1.02 - 1.11) |
|  |  |  |  | Packed RBC transfusion | 1.01 (1.00 - 1.02) |
|  |  |  |  | Anesthetic time | 1.002 (1.001- 1.003) |
| Wyssusek, K. H.;  2015 [[39](#_ENREF_39)];  Australia;  Princess Alexandra Hospital | AKI;  Scr >133 µmol/l with an increase of 50% above the baseline and/or the requirement for RRT;  Within 1 week after LT | 24/73;  Retrospective study;  Multivariate analyses with non-parametric feature selection approach;  Deceased-donor OLT;  AKI: 24/97 (24.7%). | Age, sex, Pre-Sodium, Pre-Potassium, Pre-Urea, Pre-Creatinine, Intra-RBC, Intra-FFP, Intra-Platelets, Intra-Urine output, Intra-Adrenaline use, Intraoperative hypotension | High BMI | 1.18 (1.00 - 1.40) |
|  |  |  |  | High MELD score | 1.17 (NA) |
|  |  |  |  | Intraoperative use of noradrenaline | 5.98 (1.80 - 19.88) |
| Andert, Anne;  2016 [[40](#_ENREF_40)];  Germany;  University Hospital Aachen | ARF;  An increase in Scr of more than 50% combined with oliguria, anuria, or the need for RRT;  On postoperativeday 1-14 | 16/141;  Retrospective study;  Chi squre test;  LT with an extracorporeal veno-venous/porto-systemic bypass;  ARF: 16/157 (10.2%) | - (Univariable analysis) | Dnonr BMI > 30 | 4.86 (1.65 - 14.32) |
| Erdost, H. A.;  2016 [[41](#_ENREF_41)];  Turkey;  School of Medicine, Dokuz Eylül University | ARI;  RIFLE criteria;  Within 48 hours after LT | 35/405;  Retrospective study;  Multivariate logistic regression;  LT;  ARI: 35/440 (8.0%) | Sex, Age | Preoperative hemoglobin (< 9 mg/dL) | 2.83 (1.16 - 6.93) |
|  |  |  |  | Blood loss (mL) | 1.00 (0.99 - 1.00) |
| Inoue, Yusuke;  2016 [[42](#_ENREF_42)];  Japan;  Nagasaki University Hospital | AKI;  An increase in the Scr level of 0.5mg/dL above the baseline;  Within 1 week after LDLT | 78/40;  Retrospective study;  Multivariate logistic regression;  LDLT;  AKI: 78/118 (66.1%) | - (Univariate analysis) | Sepsis | 3.28 (1.29 - 8.33) |
|  |  |  |  | Intraoperative bleeding | 4.41 (1.94 - 10.02) |
|  |  |  |  | BMI | 1.040 (1.001 - 1.080) |
|  |  |  |  | Preoperative hemoglobin | 0.899 (0.845 - 0.957) |
|  |  |  |  | Preoperative serum albumin | 0.627 (0.510 - 0.771) |
|  |  |  |  | Intraoperative FFP transfusion | 1.012 (1.001 - 1.022 |
|  |  |  |  | Operation time | 1.001 (1.000 - 1.002) |
| Laing, R. W.;  2016 [[43](#_ENREF_43)];  UK;  University Hospitals Birmingham NHS Foundation Trust | AKI;  RIFLE criteria (Peak Scr ≥ 2.0 – 2.9 times baseline);  Within 90 days after operation | 89/285;  Retrospective propensity-matched study;  Propensity score matching;  DCD-OLT;  AKI: 89/374 (23.8%) | Donor age and BMI, recipient age and BMI, recipient primary diagnosis and MELD | DCD | 2.75 (1.66 - 4.55) |
| Wadei, H. M.;  2016 [[44](#_ENREF_44)];  USA;  Mayo Clinic | AKI (stage 3) requiring RRT; AKIN criteria;  Within 1 month after LT | 71/1178;  NA;  Multivariate logistic regression;  LT;  AKI requiring RRT: 71/1249 (5.6%) | Waiting time，Donor risk index | HCV infection | 1.76 (1.07 - 3.44) |
|  |  |  |  | Operative time (per 15 min) | 0.92 (0.88 - 0.97) |
|  |  |  |  | Serum creatinine at LT (per mg/dL) | 2.05 (1.59 - 2.65） |
|  |  |  |  | RBC (per L) | 1.21 (1.13 - 1.30） |
|  |  |  |  | EAD | 2.68 (1.54 - 4.68） |
| Wiesen, Patricia;  2016 [[45](#_ENREF_45)];  Belgium;  University Hospital of Liege | AKI;  Sequential organ failure assessment score stratification (a creatinine level above 12 mg/L);  Within 1 week after LT | 109/78;  Retrospective study;  Multivariate logistic regression;  LT;  AKI: 109/187 (58.30%) | Age, Preoperative bilirubin, Preoperative urea, Ascites, Prior bacterial infection, Surgical revision, Intraoperative transfusion, Postoperatvie AST, Postoperative urea | BMI | 1.10 (1.03 - 1.18) |
|  |  |  |  | Preoperative creatinine level | 11.07 (5.28 - 23.23) |
|  |  |  |  | Intraoperative use of vasopressor | 3.31 (1.75 - 6.29) |
|  |  |  |  | Maximal postoperative bilirubin level | 1.44 (1.01 - 2.05) |
|  |  |  |  | Minimal postoperative hemoglobin level | 0.06 (0.01 - 0.29) |
| Atalan, Hakan K.;  2017 [[46](#_ENREF_46)];  Turkey;  Atasehir Memorial Hospital | AKI;  KDIGO criteria;  In postoperative day 7 | 27/193;  Retrospective study;  Multivariate logistic regression;  LDLT;  AKI: 27/220 (12.3%) | Sex, Norepinephrine requirement, MELD, GW/RW ratio | Serum tacrolimus levels (postoperative day 7 tacrolimus level ≥ 10.2ng/dL) | 2.80 (1.10 - 7.00) |
|  |  |  |  | Intraoperative blood loss ≥14.5ml/kg | 2.70 (1.02 - 7.30) |
|  |  |  |  | Intraoperative hypotension (duration of MAP below 60mmHg ≥ 5.5min) | 5.10 (1.70 - 15.00) |
|  |  |  |  | Intraoperative usage of gelatin > 5ml/kg | 3.40 (1.20 - 9.90) |
| Chae, Min Suk;  2017 [[47](#_ENREF_47)];  Korea;  St. Mary’s Hospital | AKI;  AKIN criteria;  Within 48 hours after LDLT | 76/258;  Retrospective study;  Multivariate logistic regression;  LDLT;  AKI: 76/334 (22.7%) | DM, MELD score (pts), C-reactive protein (mg/L), Severe postreperfusion syndrome, Total RBC transfusion, Total FFP transfusion, Furosemide administration | BMI | 1.10 (1.02 - 1.18) |
|  |  |  |  | Intraoperative furosemide administration | 1.02 (1.01 - 1.03) |
|  |  |  |  | Oxygen content 5 minutes after graft reperfusion | 0.83 (0.73 - 0.96) |
| Chen, Xiaohong;  2017 [[48](#_ENREF_48)];  China;  Zhongshan Hospital of Fudan University | AKI;  AKIN criteria;  Within 48 hours after LT | 109/457;  Retrospective study;  Multivariate logistic regression;  LT in hepatocellular carcinoma patients  AKI: 109/566 (19.26%) | Age, Liver cirrhosis, MELD score, Child–Pugh class, Preoperative PT, Preoperative albumin, Preoperative BUN, Operating time, Blood loss, Blood transfusion | Long anhepatic time | 3.59 (1.37 - 9.39) |
|  |  |  |  | Prolonged duration of systolic blood pressure (SBP) < 90 mmHg | 1.07 (1.05 - 1.09) |
| Jochmans, Ina;  2017 [[49](#_ENREF_49)];  Belgium;  University Hospitals Leuven | AKI;  RIFLE criteria;  At 12 hours after reperfusion | 21/59;  Prospective cohort study;  Multivariate logistic regression;  LT;  AKI: 21/80 (26%) | Cold ischemia time (h) | Peak AST occurring at 6 hours | 2.42 (1.24 - 4.91) |
| Kalisvaart, Marit; 2017 [[50](#_ENREF_50)];  Netherlands;  Erasmus MC University Medical Center | AKI;  AKIN criteria;  Within the first week after LT | 61/94;  Retrospective study;  Multivariate logistic regression;  DBD-LT;  AKI: 61/155 (39.4%) | Preoperative Scr, Intraoperative RBC transfusion; Intraoperative average dose norepinephrine | Cold ischemia time | 1.302 (1.067 - 1.590) |
|  |  |  |  | Warm ischemia time | 1.064 (1.002 - 1.130) |
|  |  |  |  | Postreperfusion syndrome | 2.283 (1.061 - 4.915) |
| Mizota, Toshiyuki; 2017 [[51](#_ENREF_51)];  Japan;  Kyoto University Hospital | Severe AKI;  Stages 2-3 according to KDIGO criteria;  During the first 7 postoperative days | 71/160;  Retrospective observational study;  Multivariate logistic regression;  LT;  Severe AKI: 71/231 (30.7%) | Sex, BMI, DM, Blood loss, Graft-recipient weight ratio, CNI overexposure | Nadir MAP during the surgery (per 10 mmHg decrease) | 2.11 (1.32 - 3.47) |
| Park, Ju Yeon;  2017 [[52](#_ENREF_52)];  Korea;  Pusan National University Yangsan Hospital | AKI;  AKIN criteria;  Within 48 hours postoperatively | 43/68;  Retrospective study;  Propensity score matching (1:2);  LT;  AKI: 43/111 (38.7%) | Age, Sex, Cause of liver transplantation, MELD score, Child-Pugh score, Presence of preoperative ascites, Hepatic encephalopathy, Esophageal varix, Hepatorenal syndrome, Preoperative medications, Underlying systemic diseases | Underweight (negative result) | 0.79 (0.35 - 1.80) |
| Rahman, Suehana;  2017 [[53](#_ENREF_53)];  UK;  Royal Free Hospital with Royal Free London NHS Foundation Trust | AKI;  AKIN criteria;  Within 24 hours after LT | 58/58;  A single-centre retrospective study;  Multivariate logistic regression;  LT;  AKI: 58/116 (50%);  CVVHF: 28/116 (24%) | Surgical technique, Transfusion of red cell concentrate, fresh frozen plasma, platelets and cell salvage blood | Peak serum AST < 24 h post OLT | 1.001 (1.00 -1.001) |
|  |  |  |  | Age | 1.12 (1.02 - 1.23) |
| Sun Kai;  2017 [[54](#_ENREF_54)]；  USA;  University of California Los Angeles Medical Center | AKI;  AKIN criteria;  Within 48 hours after LT | 106/136;  Primary LT using classic technique (complete ICV cross-clamping technique);  AKI: 569/1037 (54.9%) for the entire studied patient population , AKI in the VVB and non-VVB groups before match was 56.5% and 49.8% and 51.1% and 55.2% after  match, respectively. | Cold ischemia time, Warm ischemia time, Surgery time,  Requirement of vasopressors,  Postreperfusion syndrome,  Transfusion of red blood cells,  Transfusion of fresh frozen plasma | Use of intraoperative venovenous bypass | 0.1 (0.1 - 0.4) |
| Yoo, Seokha;  2017 [[55](#_ENREF_55)];  Korea;  Seoul National University Hospital | AKI;  RIFLE criteria;  Within 7 days after LT | 132/172;  Retrospective study;  Multivariate logistic regression with backward stepwise variable selection;  LT;  AKI: 132/304 (43.4%) | Age，Deceased donor，Emergency operation，MELD score，Initial diagnosis，Comorbidity，Total anesthesia time，Intraoperative RBC，Graft failure，Hypoglycemia | Increased perioperative glucose variability | (application of dummy variable) |
|  |  |  |  | First quartile | 1 (reference) |
|  |  |  |  | Second quartile | 1.59 (0.78 - 3.26) |
|  |  |  |  | Third quartile | 2.47 (1.22 - 5.00) |
|  |  |  |  | Fourth quartile | 2.16 (1.05 - 4.42) |
|  |  |  |  | Male | 0.42 (0.25 - 0.72) |
|  |  |  |  | Pulmonary hypertension | 3.63 (1.06 - 12.44) |
|  |  |  |  | GW/RW < 0.8 | 3.11 (1.03 - 9.37) |
| Zongyi, Yin;  2017 [[56](#_ENREF_56)];  China;  China Liver Transplant Registry | AKI;  RIFLE criteria;  Within their hospital stay period | 194/4288;  NA;  Multivariate logistic regression;  OLT;  AKI: 194/4882 (3.97%) | China classification of DCD, Cause of donor death, Donor blood examination (Sodium, BUN, Albumin, Blood sugar, Scr, AST), Blood type compatibility, Hepatocarcinoma-related disease, Cirrhosis, Hepatic failure, Preoperative Scr, Intraoperative transfusion of RBC and FFP, Autologous blood cell transfusion, Intraoperative use of noradrenaline and dobutamine, Postoperative complications, Initial induction of CNI | Cold ischemia time > 7h | 1.06 (1.03 - 1.09) |
|  |  |  |  | Warm ischemia time > 10min | 1.03 (1.01 - 1.05） |
|  |  |  |  | Intraoperative blood loss > 2500mL | 1.23 (1.00 - 1.45） |
|  |  |  |  | Preoperative Scr > 354μmol/L | 1.35 (1.18 - 1.76） |
|  |  |  |  | Treatment with dopamine > 6 days | 1.85 (1.42 - 2.28） |
|  |  |  |  | Overexposure to CNI (Yes) | 2.84 (1.76 - 5.36） |
|  |  |  |  | Combined use of MMF(No) | 2.18 (1.34 - 6.89） |
| Cheng, Yuan;  2018 [[57](#_ENREF_57)];  China;  Fuzhou General Hospital | AKI;  KDIGO criteria;  Within 1week after LT | 206/115;  Retrospective study;  Multivariate logistic regression with forward stepwise method;  OLT;  AKI: 206/321 (64.2%) | Preoperative BUN，Preoperative albuin, Preoperative total bilirubin, Preoperative PT‑INR, Intraoperative volume of fresh frozen plasma transfusion | Preoperative calculated MELD score | 1.05 (1.01 - 1.09) |
|  |  |  |  | Intraoperative volume of red cell suspension transfusion | 1.001 (1.000 - 1.001） |
|  |  |  |  | Preoperative liver cirrhosis | 2.02 (1.17 - 3.48） |
| Codes, Liana;  2018 [[58](#_ENREF_58)];  Brazil;  Portuguese Hospital of Salvador | AKI;  KDIGO criteria;  Within 7 days after LT | 87/34;  Retrospective study;  Multivariate analysis with stepwise logistic regression;  LT;  AKI: 87/121 (71.9%) | APACHE II score 24 h after admission, Number of packed red blood cell units, Use of vasoactive drugs, Peak AST levels (U/L), Early allograft dysfunction, Cumulative fluid balance in the first 12 h | Male sex | 9.29 (1.48 - 58.24) |
|  |  |  |  | Cumulative fluid balance in the first 4 d | 2.30 (1.37 - 3.86) |
| Jun, In-Gu;  2018 [[59](#_ENREF_59)];  Korea;  Asan Medical Center | AKI;  RIFLE criteria;  Within 7 days after LDLT | 1270/595;  Multivariable logistic regression analysis, propensity score matching and inverse probability of treatment weighting analysis;  LDLT;  AKI: 1270/1865 (68.1%) | Age, DM, MELD score, Vasopressor use, RBC transfusion, FFP transfusion, Total urine output, Cold ischemic time, Tacrolimus use | Postreperfusion syndrome | 1.31 (1.04 - 1.67) |
|  |  |  |  | Body mass index | 1.06 (1.01 - 1.11) |
|  |  |  |  | Preoperative hemoglobin | 0.90 (0.86 - 0.96) |
|  |  |  |  | Preoperative albumin | 0.55 (0.45 - 0.67) |
|  |  |  |  | Graft-recipient weight ratio | 0.55 (0.34 - 0.91) |
| Kalisvaart, Marit;  2018 [[60](#_ENREF_60)];  UK and Netherlands;  Queen Elizabeth Hospital, NHS Foundation Trust, and Erasmus MC University Medical Center | Severe AKI (stage 2/3);  KDIGO criteria;  Within 7 days after LT | 239/129;  2-center retrospective study;  Multivariate logistic regression;  Orthotopic DCD LT;  AKI: 239/368 (65%);  Severe AKI (stage 2/3): 151/368 (41%) | Donor age, Donor gender, Donor BMI, Recipient age, Recipient gender, Diagnosis liver disease, Hepatocellular carcinoma, MELD, Preoperative eGFR , Cold ischemia time, Tacrolimus peak level | Increasing duration of the combined WIT | 1.03 (1.01 - 1.05) |
|  |  |  |  | RBC transfusion (units) | 1.10 (1.03 - 1.18) |
|  |  |  |  | Recipient BMI | 1.05 (0.99 - 1.12) |
| Kwon, Hye-Mee;  2018 [[61](#_ENREF_61)];  South Korea;  Asan Medical Center, University of Ulsan College of Medicine | AKI;  KDIGO criteria;  Within 7 days after LT | 777/359;  Retrospective study;  Multivariate logistic regression; inverse probability treatment weighting (IPTW) of propensity score;  LDLT;  AKI: 777/1136 (68.4%);  Stage 1: 466/1136 (41.0%);  Stage 2: 233/1136 (20.5%);  Stage 3: 78/1136 (6.9%) | Sex, Hypertension, MELD score, Hepatitis B cirrhosis, Donor age, Graft-to-recipient weight ratio | BMI | 1.09 (1.04 - 1.13) |
|  |  |  |  | Early postoperative MBP (by decrease of 10 mmHg) | 1.36 (1.16 - 1.59) |
|  |  |  |  | Cystatin C GFR (by decrease of 10 ml/min/1.73 m^2^) | 1.05 (1.01 - 1.10) |
|  |  |  |  | Alcoholic cirrhosis | 1.44 (0.99 - 2.12) |
|  |  |  |  | Transfused pRBC (unit) | 1.02 (1.00 - 1.04) |
|  |  |  |  | Albumin | 0.52 (0.40 - 0.68) |
| Wang, Yue;  2018 [[62](#_ENREF_62)];  China;  First Affiliated Hospital, Xi’an Jiaotong University School of Medicine | AKI;  KDIGO criteria;  Within 7 days after LT | 71/66;  Retrospective observational study;  Multivariate logistic regression, propensity score matching;  OLT;  AKI: 71/137 (51.8%) | Gender, Age, Coexisting conditions, MELD, Blood type, Total bilirubin, Blood urea nitrogen, Albumin, Platelet, Leukocyte count,  Intraoperative blood loss | Transfusion of older blood | 2.47 (1.13 - 5.41) |
| Widmer, Jeannette D.;  2018 [[63](#_ENREF_63)]；  UK；  Royal Free Hospital | RRT;  KDIGO criteria;  Within 7 days after LT | 85/293;  Retrospective study;  Multivariate logistic regression;  DBD-LT with piggy-back (177) and conventionally cava replacement technique (201);  AKI: 56/378 (14.8%),  RRT: 85/378 (22.5%) | Donor age, Cold storage (h), Recipient age, Piggy-back vs Cava replacement, Duration of transplantation, Recipient warm ischemia time, FFP transfusion | MELD score | 1.077 (1.035 - 1.121) |
|  |  |  |  | RBC transfusion | 1.153 (1.065 - 1.248) |
| Carrier, Francois Martin;  2019 [[64](#_ENREF_64)];  Canada;  Center hospitalier de l’Université de Montréal | RRT;  KDIGO criteria;  NA | 31/496;  Retrospective cohort study;  Multivariate logistic regression;  AKI in 48 hours: 384/524 (73.3%);  AKI in 7 days: 169/518 (32.6%);  RRT: 31/527 (5.9%) | Fluid balance (L), Intraoperative phlebotomy, Age (years), Retransplantation,  Acute liver failure, DM,  Baseline CVP (mmHg), Vena cava clamping time (minutes), Baseline hemoglobin (g/L), Piggyback, Any intraoperative starch | Gender (male) | 0.33 (0.14 - 0.82) |
|  |  |  |  | MELD score | 1.07 (1.01 - 1.14) |
|  |  |  |  | Cold ischemia time (h) | 1.22 (1.04 - 1.45) |
|  |  |  |  | Baseline creatinine (10 mol/L) | 1.09 (1.01 - 1.18) |
| Kim, Won Ho;  2019 [[65](#_ENREF_65)];  Korea;  Seoul National University Hospital | AKI;  KDIGO criteria;  Within 7 days after LT | 265/265;  Retrospectively reviewed;  Multivariate logistic regression with backward stepwise variable selection, propensity score matching;  LDLT;  AKI; 265/734 (36.1%);  Stage 1: 224/734 (28.9%);  Stage 2 or 3: 41/734 (5.6%) | Age, Sex, BMI, Graft ischemic time, Prothrombin time: international normalized ratio, Total bilirubin, Scr, CTP score, History of hypertension, DM, Non-alcoholic steatohepatitis, Previous abdominal surgery, Graft-recipient body-weight ratio, Operation time, Intraoperative red blood cells transfusion amount | Preoperative hemoglobin, g/dL | 0.78 (0.66 - 0.91) |
|  |  |  |  | or Preoperative hemoglobin < 10 g/dL | 1.60 (1.11 - 2.30) |
|  |  |  |  | Previous abdominal surgery | 1.12 (1.04 - 1.24) |
|  |  |  |  | Operation time (h) | 1.10 (1.02 - 1.23) |
|  |  |  |  | Estimated blood loss per weight, per 10ml | 1.05 (1.02 - 1.08) |
|  |  |  |  | Baseline CVP, per 5 cmH_2_O increase | 1.20 (1.09 - 1.32) |
|  |  |  |  | SvO_2_ at 5 min before reperfusion, per 10% decrease | 1.45 (1.27 - 1.71) |
|  |  |  |  | Baseline right ventricular end-diastolic volume, per 10 ml increase | 1.48 (1.24 -1.78) |
|  |  |  |  | Postoperative hypoalbuminemia < 3.0 g/dL during postoperative two days | 1.18 (1.09 - 1.32) |
| Tan, Lingcan;  2019 [[66](#_ENREF_66)];  China;  West China Hospital, Sichuan University | AKI;  KDIGO criteria;  Within 48 hours after LT | 106/121;  Retrospective study;  Multivariate logistic regression with backward stepwise elimination algorithm;  LT;  AKI; 106/227 (46.7%);  Stage 1: 89/227 (39.2%);  Stage 2: 6/227 (2.6%)  Stage 3: 11/227 (4.8%)  RRT: 11/227 (4.8%) | Anhepatic phase, Intraoperative blood loss, FFP transfusion | BMI > 25 | 3.40 (1.69 - 6.86) |
|  |  |  |  | Prolonged inferior vena cava clamping | 1.02 (1.00 - 1.02) |
|  |  |  |  | Prolonged cold ischemia time | 1.07 (1.00 - 1.14） |
|  |  |  |  | Post-operative RBC requirement > 10 units | 4.88 (1.96 - 12.15） |
| Yu, Je Hyuk;  2019 [[67](#_ENREF_67)];  Korea;  Seoul National University Hospital | AKI;  KDIGO criteria;  Within 7 days after LT | 342/543;  Retrospective observational study;  Conditional logistic regression analysis with backward Wald variable selection;  LDLT;  AKI: 342/885 (38.6%) ;  Stage 1: 251/885 (28.4%); stages 2 and 3: 91/885 (10.3%) | Preoperative beta-blocker administration, Preoperative diuretics administration, Postreperfusion syndrome | BMI, recipient | 1.07 (1.01 - 1.12) |
|  |  |  |  | ABO-incompatible liver transplantation | 2.54 (1.20 - 5.38) |
|  |  |  |  | MELD score | 1.02 (1.00 - 1.04) |
|  |  |  |  | Preoperative hemoglobin, g/dL | 0.89 (0.82 - 0.97) |
|  |  |  |  | RBC transfusion, per unit | 1.09 (1.06 - 1.11) |

**Note:** AKI, acute kidney injury; OR, odds ratio; CI, confidential interval; RRT, renal replacement therapy; OLT, orthotopic liver transplantation;

MAP, mean arterial pressure; AST, aspartate aminotransferase; BUN, blood urea nitrogen; Scr, serum creatinine; ARF, acute renal failure; LT, liver transplantation; ED, early-onset renal dysfunction; MELD, model for end-stage liver disease; FFP, fresh frozen plasma; APACHE, Acute Physiology and Chronic Health Evaluation; ICU, intensive care unit; CVVHF, continuous veno-venous hemofiltration; RBC, red blood cell; CRRT, continuous renal replacement therapy; UNOS, united network for organ sharing; ARI, acute renal injury; ALT, alanine aminotransferase; RIFLE, Risk, Injury, Failure, Loss of kidney function, and End-stage kidney disease; CNI, calcineurin inhibitor; IAH, intra-abdominal hypertension; AKIN, acute kidney injury network; BSA, body surface area; PT, prothrombin time; BMI, body mass index; CTP, Child-Turcotte-Pugh; AKD, acute kidney dysfunction; DM, diabetes mellitus; DCD, donation after cardiac death; eGFR, estimated glomerular filtration rate; DBD, donation after brain death; INR, international normalized ratio; LDLT, living donor liver transplantation; GW/RBW: graft to recipient body weight ratio; SLT, split liver transplantation; FSLT, full-size liver transplantation; PRS, propensity risk score; CVP, central venous pressure; CDLT, cadaveric donor liver transplantation; VVBP, veno-venous bypass; GRWR, graft to recipient body weight ratio; MMF, mycophenolate mofetil; EAD, early allograft dysfunction; GW/RW, graft weight/recipient weight; SBP, systolic blood pressure; ICV, inferior vena cava; VVB, venovenous bypass; KDIGO, Kidney Disease Improving Global Outcome; WIT, warm ischemia time; MBP, mean blood pressure; pRBC, packed red blood cell; SvO_2_, mixed venous oxygen saturation.

**Reference:**

1. Gabriel C GG, Andrew A. Q,Cynthia C, Mark A. L., Gaspar A. B.,David R., Emilio G,: **An Epidemiologic Study of Early Renal Replacement Therapy after Orthotopic Liver Transplantation**. *J Am Soc Nephrol* 2001, **13**:228–233.

2. Lima EQ, Zanetta DMT, Castro I, Massarollo PCB, Mies S, Machado MM, Yu L: **Risk factors for development of acute renal failure after liver transplantation**. *Ren Fail* 2003, **25**(4):553-560.

3. Lebrón Gallardo M, Herrera Gutierrez ME, Seller Pérez G, Curiel Balsera E, Fernández Ortega JF, Quesada García G: **Risk factors for renal dysfunction in the postoperative course of liver transplant**. *Liver Transpl* 2004, **10**(11):1379-1385.

4. Sanchez EQ, Gonwa TA, Levy MF, Goldstein RM, Mai ML, Hays SR, Melton LB, Saracino G, Klintmalm GB: **Preoperative and perioperative predictors of the need for renal replacement therapy after orthotopic liver transplantation**. *Transplantation* 2004, **78**(7):1048-1054.

5. Cabezuelo JB, Ramírez P, Ríos A, Acosta F, Torres D, Sansano T, Pons JA, Bru M, Montoya M, Bueno FS *et al*: **Risk factors of acute renal failure after liver transplantation**. *Kidney Int* 2006, **69**(6):1073-1080.

6. Guitard J, Cointault O, Kamar N, Muscari F, Lavayssière L, Suc B, Ribes D, Esposito L, Barange K, Durand D *et al*: **Acute renal failure following liver transplantation with induction therapy**. *Clin Nephrol* 2006, **65**(2):103-112.

7. Wei Y, Zhang L, Lin H, Li J, Li B, Yan L, Wen T, Zeng Y, Lu S: **Factors related to post-liver transplantation acute renal failure**. *Transplant Proc* 2006, **38**(9):2982-2984.

8. O'Riordan A, Wong V, McQuillan R, McCormick PA, Hegarty JE, Watson AJ: **Acute renal disease, as defined by the RIFLE criteria, post-liver transplantation**. *Am J Transplant* 2007, **7**(1):168-176.

9. Shu M, Peng C, Chen H, Shen B, Zhou G, Shen C, Li H: **Intra-abdominal hypertension is an independent cause of acute renal failure after orthotopic liver transplantation**. *Front Med China* 2007, **1**(2):167-172.

10. Iglesias JI, DePalma JA, Levine JS: **Risk factors for acute kidney injury following orthotopic liver transplantation: the impact of changes in renal function while patients await transplantation**. *BMC Nephrol* 2010, **11**:30-30.

11. Kundakci A, Pirat A, Komurcu O, Torgay A, Karakayalı H, Arslan G, Haberal M: **Rifle criteria for acute kidney dysfunction following liver transplantation: incidence and risk factors**. *Transplant Proc* 2010, **42**(10):4171-4174.

12. Sakai T, Matsusaki T, Marsh JW, Hilmi IA, Planinsic RM: **Comparison of surgical methods in liver transplantation: retrohepatic caval resection with venovenous bypass (VVB) versus piggyback (PB) with VVB versus PB without VVB**. *Transpl Int* 2010, **23**(12):1247-1258.

13. Xu X, Ling Q, Wei Q, Wu J, Gao F, He Z-L, Zhou L, Zheng S-S: **An effective model for predicting acute kidney injury after liver transplantation**. *Hepatobiliary Pancreat Dis Int* 2010, **9**(3):259-263.

14. Zhu M, Li Y, Xia Q, Wang S, Qiu Y, Che M, Dai H, Qian J, Ni Z, Axelsson J *et al*: **Strong impact of acute kidney injury on survival after liver transplantation**. *Transplant Proc* 2010, **42**(9):3634-3638.

15. Chen J, Singhapricha T, Hu K-Q, Hong JC, Steadman RH, Busuttil RW, Xia VW: **Postliver transplant acute renal injury and failure by the RIFLE criteria in patients with normal pretransplant serum creatinine concentrations: a matched study**. *Transplantation* 2011, **91**(3):348-353.

16. Leithead JA, Tariciotti L, Gunson B, Holt A, Isaac J, Mirza DF, Bramhall S, Ferguson JW, Muiesan P: **Donation after cardiac death liver transplant recipients have an increased frequency of acute kidney injury**. *Am J Transplant* 2012, **12**(4):965-975.

17. Leithead JA, Armstrong MJ, Corbett C, Andrew M, Kothari C, Gunson BK, Muiesan P, Ferguson JW: **Hepatic ischemia reperfusion injury is associated with acute kidney injury following donation after brain death liver transplantation**. *Transpl Int* 2013, **26**(11):1116-1125.

18. Liu S, Wang X, Lu Y, Li T, Gong Z, Sheng T, Hu B, Peng Z, Sun X: **The effects of intraoperative cryoprecipitate transfusion on acute renal failure following orthotropic liver transplantation**. *Hepatol Int* 2013, **7**(3):901-909.

19. Romano TG, Schmidtbauer I, Silva FMdQ, Pompilio CE, D'Albuquerque LAC, Macedo E: **Role of MELD score and serum creatinine as prognostic tools for the development of acute kidney injury after liver transplantation**. *PLoS One* 2013, **8**(5):e64089-e64089.

20. Utsumi M, Umeda Y, Sadamori H, Nagasaka T, Takaki A, Matsuda H, Shinoura S, Yoshida R, Nobuoka D, Satoh D *et al*: **Risk factors for acute renal injury in living donor liver transplantation: evaluation of the RIFLE criteria**. *Transpl Int* 2013, **26**(8):842-852.

21. Karapanagiotou A, Dimitriadis C, Papadopoulos S, Kydona C, Kefsenidis S, Papanikolaou V, Gritsi-Gerogianni N: **Comparison of RIFLE and AKIN criteria in the evaluation of the frequency of acute kidney injury in post-liver transplantation patients**. *Transplant Proc* 2014, **46**(9):3222-3227.

22. Klaus F, Keitel da Silva C, Meinerz G, Carvalho LM, Goldani JC, Cantisani G, Zanotelli ML, Duro Garcia V, Keitel E: **Acute kidney injury after liver transplantation: incidence and mortality**. *Transplant Proc* 2014, **46**(6):1819-1821.

23. Leithead JA, Armstrong MJ, Corbett C, Andrew M, Kothari C, Gunson BK, Mirza D, Muiesan P, Ferguson JW: **Split liver transplant recipients do not have an increased frequency of acute kidney injury**. *Transpl Int* 2014, **27**(11):1125-1134.

24. Leithead JA, Rajoriya N, Gunson BK, Muiesan P, Ferguson JW: **The evolving use of higher risk grafts is associated with an increased incidence of acute kidney injury after liver transplantation**. *J Hepatol* 2014, **60**(6):1180-1186.

25. Nadeem A, Salahuddin N, El Hazmi A, Joseph M, Bohlega B, Sallam H, Sheikh Y, Broering D: **Chloride-liberal fluids are associated with acute kidney injury after liver transplantation**. *Crit Care* 2014, **18**(6):625-625.

26. Papadopoulos S, Karapanagiotou A, Kydona C, Dimitriadis C, Theodoridou T, Piperidou M, Imvrios G, Fouzas I, Gritsi-Gerogianni N: **Causes and incidence of renal replacement therapy application in orthotopic liver transplantation patients: our experience**. *Transplant Proc* 2014, **46**(9):3228-3231.

27. Sirivatanauksorn Y, Parakonthun T, Premasathian N, Limsrichamrern S, Mahawithitwong P, Kositamongkol P, Tovikkai C, Asavakarn S: **Renal dysfunction after orthotopic liver transplantation**. *Transplant Proc* 2014, **46**(3):818-821.

28. Smoter P, Nyckowski P, Grat M, Patkowski W, Zieniewicz K, Wronka K, Hinderer B, Morawski M: **Risk factors of acute renal failure after orthotopic liver transplantation: single-center experience**. *Transplant Proc* 2014, **46**(8):2786-2789.

29. Kim JM, Jo YY, Na SW, Kim SI, Choi YS, Kim NO, Park JE, Koh SO: **The predictors for continuous renal replacement therapy in liver transplant recipients**. *Transplant Proc* 2014, **46**(1):184-191.

30. Aksu Erdost H, Ozkardesler S, Ocmen E, Avkan-Oguz V, Akan M, Iyilikci L, Unek T, Ozbilgin M, Meseri Dalak R, Astarcioglu I: **Acute Renal Injury Evaluation After Liver Transplantation: With RIFLE Criteria**. *Transplant Proc* 2015, **47**(5):1482-1487.

31. Barreto AGC, Daher EF, Silva Junior GB, Garcia JHP, Magalhães CBA, Lima JMC, Viana CFG, Pereira EDB: **Risk factors for acute kidney injury and 30-day mortality after liver transplantation**. *Ann Hepatol* 2015, **14**(5):688-694.

32. Chen H-P, Tsai Y-F, Lin J-R, Liu F-C, Yu H-P: **Incidence and Outcomes of Acute Renal Failure Following Liver Transplantation: A Population-Based Cohort Study**. *Medicine (Baltimore)* 2015, **94**(52):e2320-e2320.

33. Hand WR, Whiteley JR, Epperson TI, Tam L, Crego H, Wolf B, Chavin KD, Taber DJ: **Hydroxyethyl starch and acute kidney injury in orthotopic liver transplantation: a single-center retrospective review**. *Anesth Analg* 2015, **120**(3):619-626.

34. Hilmi IA, Damian D, Al-Khafaji A, Planinsic R, Boucek C, Sakai T, Chang CCH, Kellum JA: **Acute kidney injury following orthotopic liver transplantation: incidence, risk factors, and effects on patient and graft outcomes**. *Br J Anaesth* 2015, **114**(6):919-926.

35. Hilmi IA, Damian D, Al-Khafaji A, Sakai T, Donaldson J, Winger DG, Kellum JA: **Acute kidney injury after orthotopic liver transplantation using living donor versus deceased donor grafts: A propensity score-matched analysis**. *Liver Transpl* 2015, **21**(9):1179-1185.

36. Mukhtar A, Mahmoud I, Obayah G, Hasanin A, Aboul-Fetouh F, Dabous H, Bahaa M, Abdelaal A, Fathy M, El Meteini M: **Intraoperative terlipressin therapy reduces the incidence of postoperative acute kidney injury after living donor liver transplantation**. *J Cardiothorac Vasc Anesth* 2015, **29**(3):678-683.

37. Park MH, Shim HS, Kim WH, Kim H-J, Kim DJ, Lee S-H, Kim CS, Gwak MS, Kim GS: **Clinical Risk Scoring Models for Prediction of Acute Kidney Injury after Living Donor Liver Transplantation: A Retrospective Observational Study**. *PLoS One* 2015, **10**(8):e0136230-e0136230.

38. Sang B-H, Bang J-Y, Song J-G, Hwang G-S: **Hypoalbuminemia Within Two Postoperative Days Is an Independent Risk Factor for Acute Kidney Injury Following Living Donor Liver Transplantation: A Propensity Score Analysis of 998 Consecutive Patients**. *Crit Care Med* 2015, **43**(12):2552-2561.

39. Wyssusek KH, Keys ALB, Yung J, Moloney ET, Sivalingam P, Paul SK: **Evaluation of perioperative predictors of acute kidney injury post orthotopic liver transplantation**. *Anaesth Intensive Care* 2015, **43**(6):757-763.

40. Andert A, Becker N, Ulmer F, Schöning W, Hein M, Rimek A, Neumann U, Schmeding M: **Liver Transplantation and Donor Body Mass Index >30: Use or Refuse?** *Ann Transplant* 2016, **21**:185-193.

41. Erdost HA, Ozkardesler S, Akan M, Iyilikci L, Unek T, Ocmen E, Dalak RM, Astarcioglu I: **Comparison of the RIFLE, AKIN, and KDIGO Diagnostic Classifications for Acute Renal Injury in Patients Undergoing Liver Transplantation**. *Transplant Proc* 2016, **48**(6):2112-2118.

42. Inoue Y, Soyama A, Takatsuki M, Hidaka M, Kinoshita A, Natsuda K, Baimakhanov Z, Kugiyama T, Adachi T, Kitasato A *et al*: **Does the development of chronic kidney disease and acute kidney injury affect the prognosis after living donor liver transplantation?** *Clin Transplant* 2016, **30**(5):518-527.

43. Laing RW, Scalera I, Isaac J, Mergental H, Mirza DF, Hodson J, Wilkin RJW, Perera MTPR, Muiesan P: **Liver Transplantation Using Grafts From Donors After Circulatory Death: A Propensity Score-Matched Study From a Single Center**. *Am J Transplant* 2016, **16**(6):1795-1804.

44. Wadei HM, Lee DD, Croome KP, Mai ML, Golan E, Brotman R, Keaveny AP, Taner CB: **Early Allograft Dysfunction After Liver Transplantation Is Associated With Short- and Long-Term Kidney Function Impairment**. *Am J Transplant* 2016, **16**(3):850-859.

45. Wiesen P, Massion PB, Joris J, Detry O, Damas P: **Incidence and risk factors for early renal dysfunction after liver transplantation**. *World J Transplant* 2016, **6**(1):220-232.

46. Atalan HK, Gucyetmez B, Aslan S, Yazar S, Polat KY: **Postoperative acute kidney injury in living donor liver transplantation recipients**. *Int J Artif Organs* 2017:0-0.

47. Chae MS, Lee N, Park DH, Lee J, Jung HS, Park CS, Lee J, Choi JH, Hong SH: **Influence of oxygen content immediately after graft reperfusion on occurrence of postoperative acute kidney injury in living donor liver transplantation**. *Medicine (Baltimore)* 2017, **96**(31):e7626-e7626.

48. Chen X, Ding X, Shen B, Teng J, Zou J, Wang T, Zhou J, Chen N, Zhang B: **Incidence and outcomes of acute kidney injury in patients with hepatocellular carcinoma after liver transplantation**. *J Cancer Res Clin Oncol* 2017, **143**(7):1337-1346.

49. Jochmans I, Meurisse N, Neyrinck A, Verhaegen M, Monbaliu D, Pirenne J: **Hepatic ischemia/reperfusion injury associates with acute kidney injury in liver transplantation: Prospective cohort study**. *Liver Transpl* 2017, **23**(5):634-644.

50. Kalisvaart M, de Haan JE, Hesselink DA, Polak WG, Hansen BE, Ijzermans JNM, Gommers D, Metselaar HJ, de Jonge J: **The postreperfusion syndrome is associated with acute kidney injury following donation after brain death liver transplantation**. *Transpl Int* 2017, **30**(7):660-669.

51. Mizota T, Hamada M, Matsukawa S, Seo H, Tanaka T, Segawa H: **Relationship Between Intraoperative Hypotension and Acute Kidney Injury After Living Donor Liver Transplantation: A Retrospective Analysis**. *J Cardiothorac Vasc Anesth* 2017, **31**(2):582-589.

52. Park JY, Park J-H, Lee SS, Ri H-S, Kim H-J, Choi YM, Choi YJ, Yoon J-U: **The Association of Preoperative Body Mass Index with Acute Kidney Injury in Liver Transplantation Recipients: A Retrospective Study**. *Korean J Crit Care Med* 2017, **32**(3):265-274.

53. Rahman S, Davidson BR, Mallett SV: **Early acute kidney injury after liver transplantation: Predisposing factors and clinical implications**. *World J Hepatol* 2017, **9**(18):823-832.

54. Sun K, Hong F, Wang Y, Agopian VG, Yan M, Busuttil RW, Steadman RH, Xia VW: **Venovenous Bypass Is Associated With a Lower Incidence of Acute Kidney Injury After Liver Transplantation in Patients With Compromised Pretransplant Renal Function**. *Anesth Analg* 2017, **125**(5):1463-1470.

55. Yoo S, Lee H-J, Lee H, Ryu H-G: **Association Between Perioperative Hyperglycemia or Glucose Variability and Postoperative Acute Kidney Injury After Liver Transplantation: A Retrospective Observational Study**. *Anesth Analg* 2017, **124**(1):35-41.

56. Zongyi Y, Baifeng L, Funian Z, Hao L, Xin W: **Risk factors of acute kidney injury after orthotopic liver transplantation in China**. *Sci Rep* 2017, **7**:41555-41555.

57. Cheng Y, Wei G-Q, Cai Q-C, Jiang Y, Wu A-P: **Prognostic Value of Model for End-Stage Liver Disease Incorporating with Serum Sodium Score for Development of Acute Kidney Injury after Liver Transplantation**. *Chin Med J (Engl)* 2018, **131**(11):1314-1320.

58. Codes L, de Souza YG, D'Oliveira RAC, Bastos JLA, Bittencourt PL: **Cumulative positive fluid balance is a risk factor for acute kidney injury and requirement for renal replacement therapy after liver transplantation**. *World J Transplant* 2018, **8**(2):44-51.

59. Jun I-G, Kwon H-M, Jung K-W, Moon Y-J, Shin W-J, Song J-G, Hwang G-S: **The Impact of Postreperfusion Syndrome on Acute Kidney Injury in Living Donor Liver Transplantation: A Propensity Score Analysis**. *Anesth Analg* 2018, **127**(2):369-378.

60. Kalisvaart M, Schlegel A, Umbro I, de Haan JE, Scalera I, Polak WG, Ijzermans JNM, Mirza DF, Perera MTPR, Isaac JI *et al*: **The Impact of Combined Warm Ischemia Time on Development of Acute Kidney Injury in Donation After Circulatory Death Liver Transplantation: Stay Within the Golden Hour**. *Transplantation* 2018, **102**(5):783-793.

61. Kwon H-M, Moon Y-J, Jung K-W, Jeong H-W, Park Y-S, Jun I-G, Song J-G, Hwang G-S: **Low Mean Arterial Blood Pressure is Independently Associated with Postoperative Acute Kidney Injury After Living Donor Liver Transplantation: A Propensity Score Weighing Analysis**. *Ann Transplant* 2018, **23**:236-245.

62. Wang Y, Li Q, Ma T, Liu X, Wang B, Wu Z, Dang S, Lv Y, Wu R: **Transfusion of Older Red Blood Cells Increases the Risk of Acute Kidney Injury After Orthotopic Liver Transplantation: A Propensity Score Analysis**. *Anesth Analg* 2018, **127**(1):202-209.

63. Widmer JD, Schlegel A, Ghazaly M, Richie Davidson B, Imber C, Sharma D, Malago M, Pollok J-M: **Piggyback or Cava Replacement: Which Implantation Technique Protects Liver Recipients From Acute Kidney Injury and Complications?** *Liver Transpl* 2018, **24**(12):1746-1756.

64. Carrier FM, Chassé M, Wang HT, Aslanian P, Iorio S, Bilodeau M, Turgeon A: **Perioperative fluid management strategies and postoperative outcomes in liver transplantation: A systematic review**. *Transplantation* 2019, **103**(8):74.

65. Kim WH, Oh H-W, Yang S-M, Yu JH, Lee H-C, Jung C-W, Suh K-S, Lee KH: **Intraoperative Hemodynamic Parameters and Acute Kidney Injury After Living Donor Liver Transplantation**. *Transplantation* 2019, **103**(9):1877-1886.

66. Tan L, Yang Y, Ma G, Zhu T, Yang J, Liu H, Zhang W: **Early acute kidney injury after liver transplantation in patients with normal preoperative renal function**. *Clin Res Hepatol Gastroenterol* 2019, **43**(4):475-482.

67. Yu JH, Kwon Y, Kim J, Yang S-M, Kim WH, Jung C-W, Suh K-S, Lee KH: **Influence of Transfusion on the Risk of Acute Kidney Injury: ABO-Compatible versus ABO-Incompatible Liver Transplantation**. *J Clin Med* 2019, **8**(11):1785.

**Supplementary 5: Quality evaluation of included studies**

| First Author | Year | S1 | S2 | S3 | S4 | C | E1 | E2 | E3 | sum |
| --- | --- | --- | --- | --- | --- | --- | --- | --- | --- | --- |
| Gabriel Contras | 2001 | ★ | - | - | ★ | ★★ | ★ | ★ | - | 6 |
| Lima, Emerson Q. | 2003 | ★ | - | - | ★ | ★★ | ★ | ★ | - | 6 |
| Lebrón Gallardo, Miguel | 2004 | ★ | - | - | ★ | ★★ | ★ | ★ | - | 6 |
| Sanchez, Edmund Q. | 2004 | - | ★ | - | ★ | ★★ | ★ | ★ | - | 6 |
| Cabezuelo, J. B. | 2006 | ★ | ★ | - | ★ | ★★ | ★ | ★ | - | 7 |
| Guitard, J. | 2006 | ★ | ★ | - | ★ | ★★ | ★ | ★ | - | 7 |
| Wei, Y. | 2006 | ★ | ★ | - | ★ | ★★ | ★ | ★ | - | 7 |
| O'Riordan, A. | 2007 | ★ | ★ | - | ★ | ★★ | ★ | ★ | - | 7 |
| Shu, Ming | 2007 | ★ | ★ | - | ★ | ★★ | ★ | ★ | - | 7 |
| Iglesias, Jose I. | 2010 | ★ | ★ | - | ★ | ★★ | ★ | ★ | - | 7 |
| Kundakci, A. | 2010 | ★ | ★ | - | ★ | ★★ | ★ | ★ | - | 7 |
| Sakai, Tetsuro | 2010 | ★ | ★ | - | ★ | - | ★ | ★ | - | 5 |
| Xu, Xiao | 2010 | ★ | ★ | - | ★ | ★★ | ★ | ★ | - | 7 |
| Zhu, M. | 2010 | ★ | ★ | - | ★ | ★★ | ★ | ★ | - | 7 |
| Chen, Jie | 2011 | ★ | ★ | - | ★ | ★★ | ★ | ★ | - | 7 |
| Leithead, J. A. | 2012 | ★ | ★ | - | ★ | ★★ | ★ | ★ | - | 7 |
| Leithead, J. A. | 2013 | ★ | ★ | - | ★ | ★★ | ★ | ★ | - | 7 |
| Liu, Shuang | 2013 | ★ | ★ | - | ★ | ★★ | ★ | ★ | - | 7 |
| Romano, Thiago Gomes | 2013 | ★ | ★ | - | ★ | ★★ | ★ | ★ | - | 7 |
| Utsumi, Masashi | 2013 | ★ | ★ | - | ★ | ★★ | ★ | ★ | - | 7 |
| Karapanagiotou, A. | 2014 | ★ | ★ | - | ★ | - | ★ | ★ | - | 5 |
| Klaus, F. | 2014 | ★ | - | - | ★ | ★★ | ★ | ★ | - | 6 |
| Leithead, Joanna A. | 2014 | ★ | ★ | - | ★ | ★★ | ★ | ★ | - | 7 |
| Leithead, Joanna A. | 2014 | ★ | ★ | - | ★ | ★★ | ★ | ★ | - | 7 |
| Nadeem, Ashraf | 2014 | ★ | ★ | - | ★ | ★★ | ★ | ★ | - | 7 |
| Papadopoulos, S. | 2014 | ★ | ★ | - | ★ | ★★ | ★ | ★ | - | 7 |
| Sirivatanauksorn, Y. | 2014 | ★ | ★ | - | ★ | ★★ | ★ | ★ | - | 7 |
| Smoter, P. | 2014 | - | - | - | ★ | ★★ | ★ | ★ | - | 5 |
| J.M. Kim | 2014 | - | - | - | ★ | ★★ | ★ | ★ | - | 5 |
| Aksu Erdost, H. | 2015 | ★ | - | - | ★ | ★★ | ★ | ★ | - | 6 |
| Barreto, Adller G. C. | 2015 | ★ | ★ | - | ★ | ★★ | ★ | ★ | - | 7 |
| Chen, Hsiu-Pin | 2015 | - | ★ | - | ★ | ★★ | ★ | ★ | - | 6 |
| Hand, William R. | 2015 | ★ | ★ | - | ★ | ★★ | ★ | ★ | - | 7 |
| Hilmi, I. A. | 2015 | ★ | ★ | - | ★ | ★★ | ★ | ★ | - | 7 |
| Hilmi, Ibtesam A. | 2015 | ★ | ★ | - | ★ | ★★ | ★ | ★ | - | 7 |
| Mukhtar, Ahmed | 2015 | ★ | ★ | - | ★ | ★★ | ★ | ★ | - | 7 |
| Park, Mi Hye | 2015 | ★ | ★ | - | ★ | ★★ | ★ | ★ | - | 7 |
| Sang, Bo-Hyun | 2015 | ★ | ★ | - | ★ | ★★ | ★ | ★ | - | 7 |
| Wyssusek, K. H. | 2015 | ★ | ★ | - | ★ | ★★ | ★ | ★ | - | 7 |
| Andert, Anne | 2016 | ★ | ★ | - | ★ | - | ★ | ★ | - | 5 |
| Erdost, H. A. | 2016 | ★ | - | - | ★ | ★★ | ★ | ★ | - | 6 |
| Inoue, Yusuke | 2016 | ★ | ★ | - | ★ | - | ★ | ★ | - | 5 |
| Laing, R. W. | 2016 | ★ | - | - | ★ | ★★ | ★ | ★ | - | 6 |
| Wadei, H. M. | 2016 | ★ | ★ | - | ★ | ★★ | ★ | ★ | - | 7 |
| Wiesen, Patricia | 2016 | ★ | ★ | - | ★ | ★★ | ★ | ★ | - | 7 |
| Atalan, Hakan K. | 2017 | ★ | ★ | - | ★ | ★★ | ★ | ★ | - | 7 |
| Chae, Min Suk | 2017 | ★ | ★ | - | ★ | ★★ | ★ | ★ | - | 7 |
| Chen, Xiaohong | 2017 | ★ | ★ | - | ★ | ★★ | ★ | ★ | - | 7 |
| Jochmans, Ina | 2017 | ★ | ★ | - | ★ | ★★ | ★ | ★ | - | 7 |
| Kalisvaart, Marit | 2017 | ★ | ★ | - | ★ | ★★ | ★ | ★ | - | 7 |
| Mizota, Toshiyuki | 2017 | ★ | ★ | - | ★ | ★★ | ★ | ★ | - | 7 |
| Park, Ju Yeon | 2017 | ★ | ★ | - | ★ | ★★ | ★ | ★ | - | 7 |
| Rahman, Suehana | 2017 | ★ | ★ | - | ★ | ★★ | ★ | ★ | - | 7 |
| Sun, Kai | 2017 | ★ | ★ | - | ★ | ★★ | ★ | ★ | - | 7 |
| Yoo, Seokha | 2017 | ★ | ★ | - | ★ | ★★ | ★ | ★ | - | 7 |
| Zongyi, Yin | 2017 | ★ | ★ | - | ★ | ★★ | ★ | ★ | - | 7 |
| Cheng, Yuan | 2018 | ★ | ★ | - | ★ | ★★ | ★ | ★ | - | 7 |
| Codes, Liana | 2018 | ★ | ★ | - | ★ | ★★ | ★ | ★ | - | 7 |
| Jun, In-Gu | 2018 | ★ | ★ | - | ★ | ★★ | ★ | ★ | - | 7 |
| Kalisvaart, Marit | 2018 | ★ | ★ | - | ★ | ★★ | ★ | ★ | - | 7 |
| Kwon, Hye-Mee | 2018 | ★ | ★ | - | ★ | ★★ | ★ | ★ | - | 7 |
| Wang, Yue | 2018 | ★ | ★ | - | ★ | ★★ | ★ | ★ | - | 7 |
| Widmer, Jeannette D. | 2018 | ★ | ★ | - | ★ | ★★ | ★ | ★ | - | 7 |
| Carrier, François Martin | 2019 | ★ | ★ | - | ★ | ★★ | ★ | ★ | - | 7 |
| Kim, Won Ho | 2019 | ★ | ★ | - | ★ | ★★ | ★ | ★ | - | 7 |
| Tan, Lingcan | 2019 | ★ | ★ | - | ★ | ★★ | ★ | ★ | - | 7 |
| Yu, Je Hyuk | 2019 | ★ | ★ | - | ★ | ★★ | ★ | ★ | - | 7 |

**Note:** S1, Is the case definition adequate?; S2, Representativeness of the cases; S3, Selection of controls; S4, Definition of controls; Comparability, E1, Ascertainment of exposure; E2, Same method of ascertainment for cases and controls; E3, Non-Response rate.

A study can be awarded a maximum of one star for each numbered item within the Selection and Exposure categories. A maximum of two stars can be given for Comparability.

**Supplementary 6: Forest plots of factors described in at least 2 studies**

Age (per year)


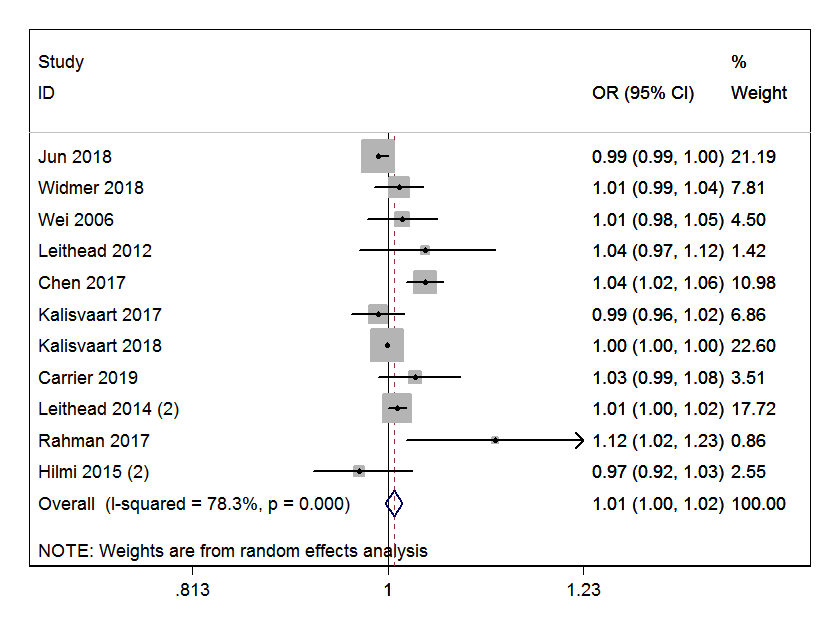
Older age


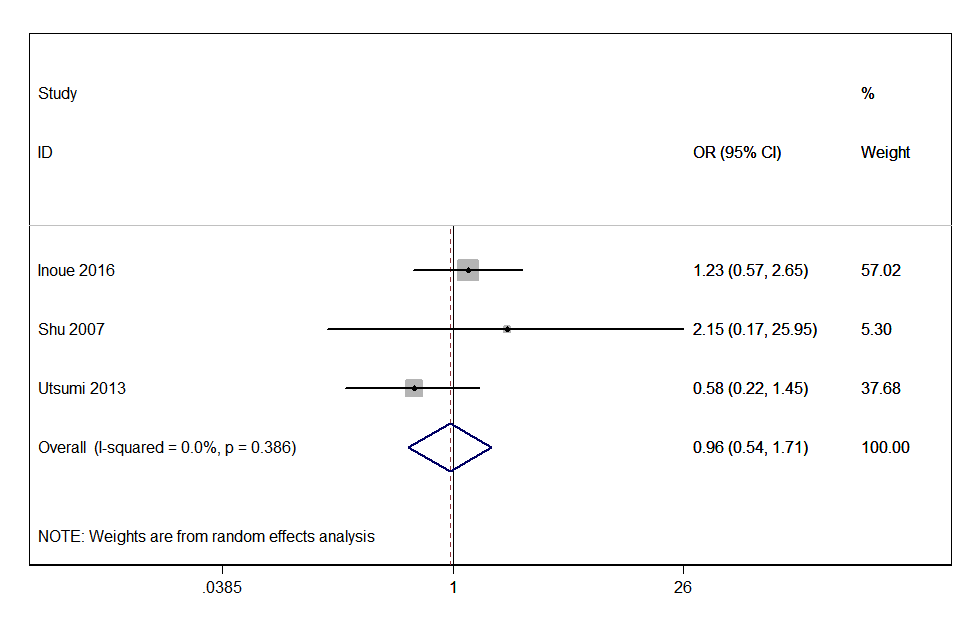


Female gender


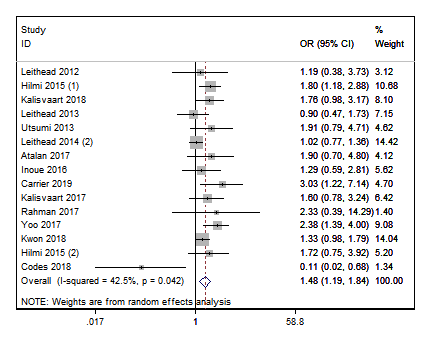


Weight (per kg)


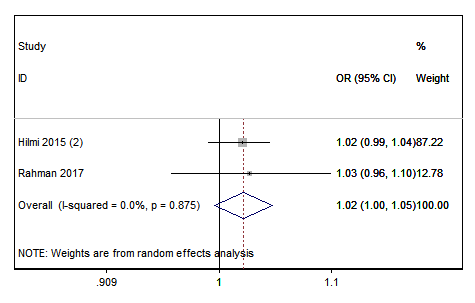


BMI (per kg/m^2^)


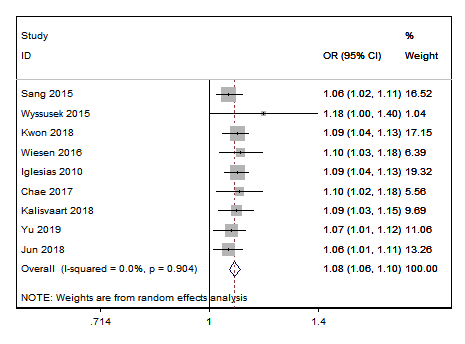


Overweight


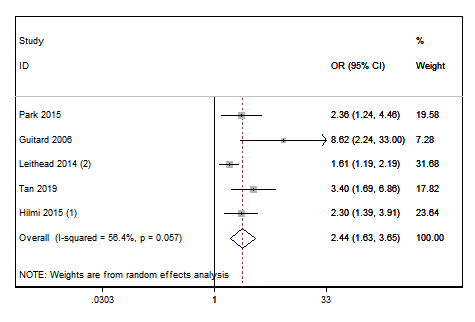


White race


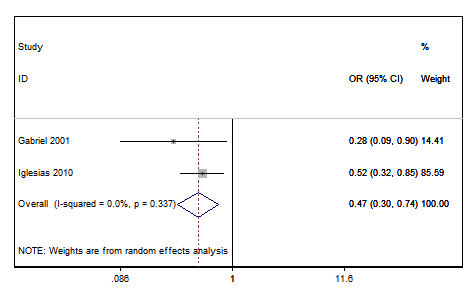


Hepatocellular carcinoma


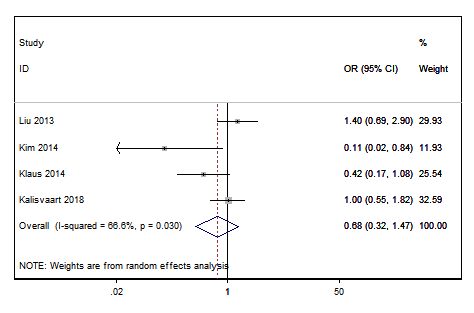


Fulminant hepatic failure


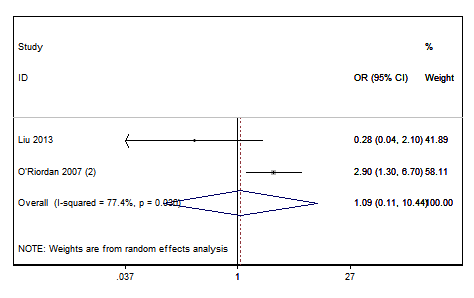


Alcoholic liver disease


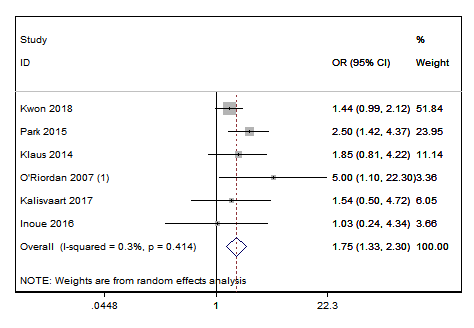


Primary biliary cirrhosis


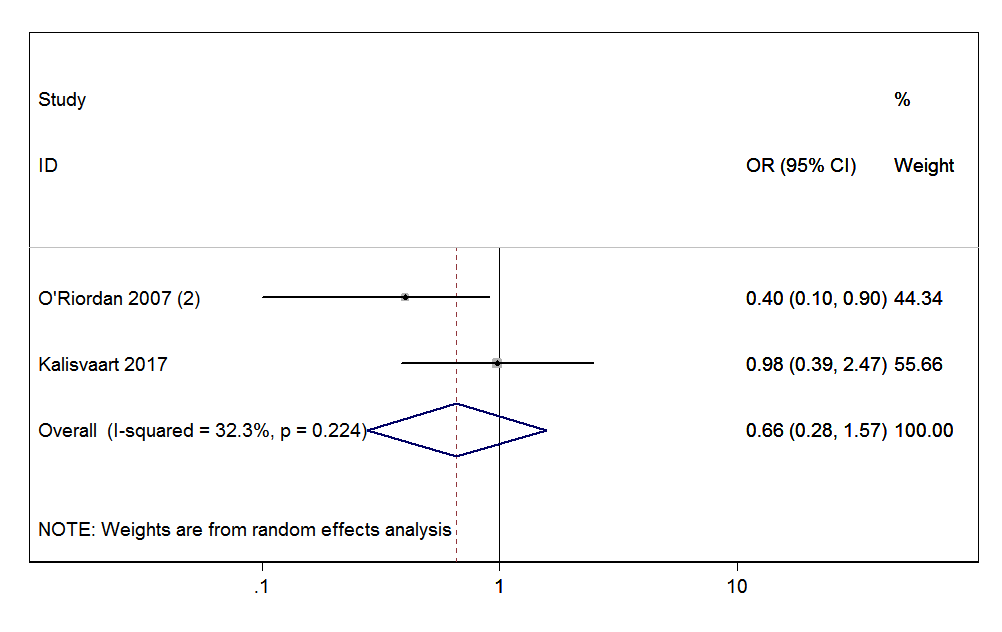


Hepatitis B virus infection


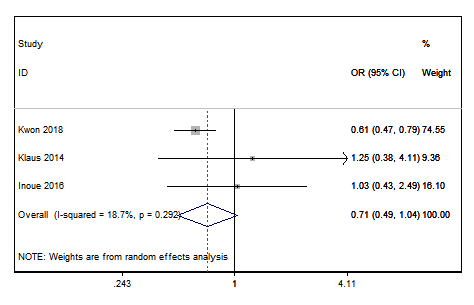


Hepatitis C virus infection


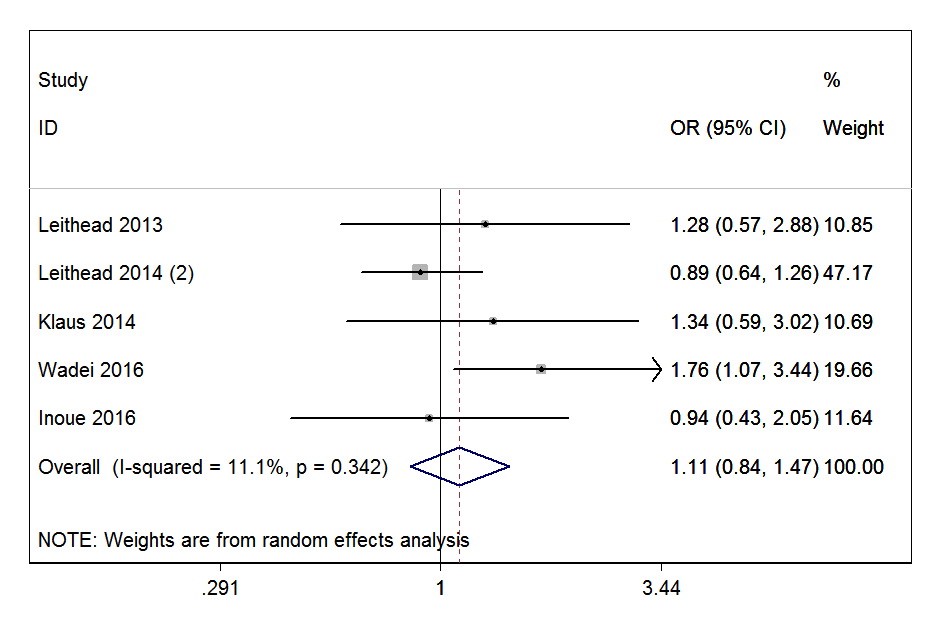


Cirrhosis


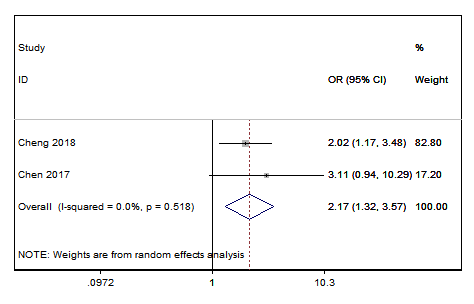


Refractory ascites


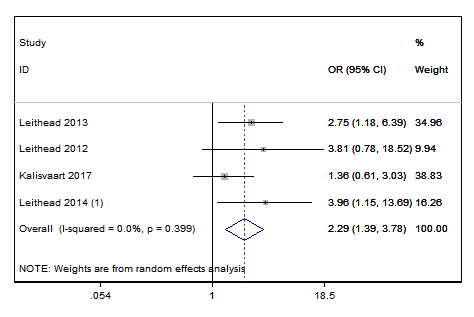


Pre-existing diabetes mellitus


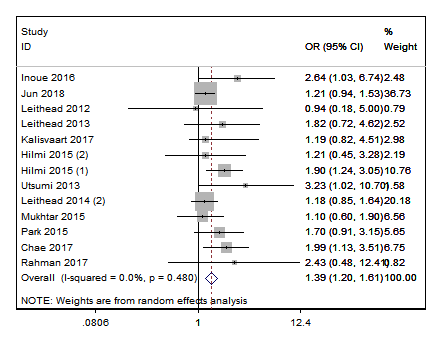


Preoperative hypertension


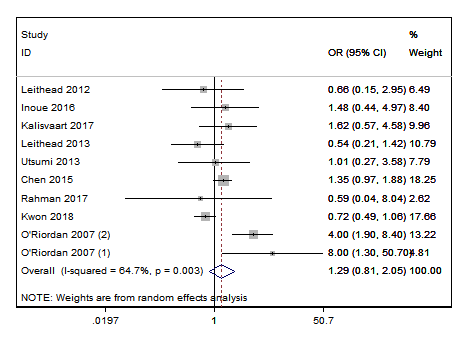


Preoperative use of diuretic


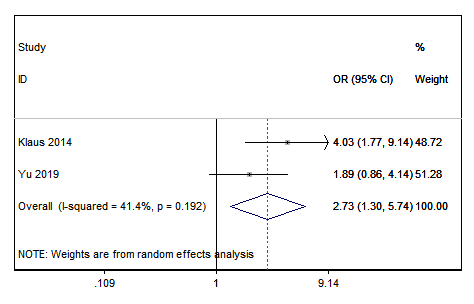


Child-Turcotte-Pugh grade C


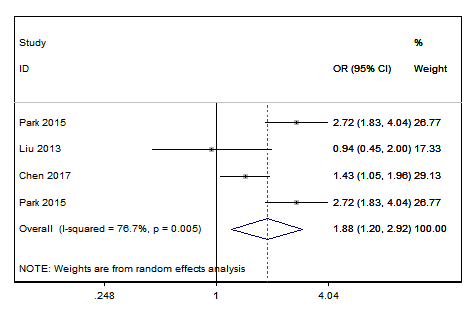


Child-Turcotte-Pugh score (per score)


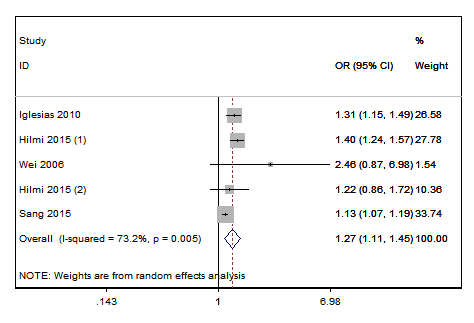


MELD (per score)


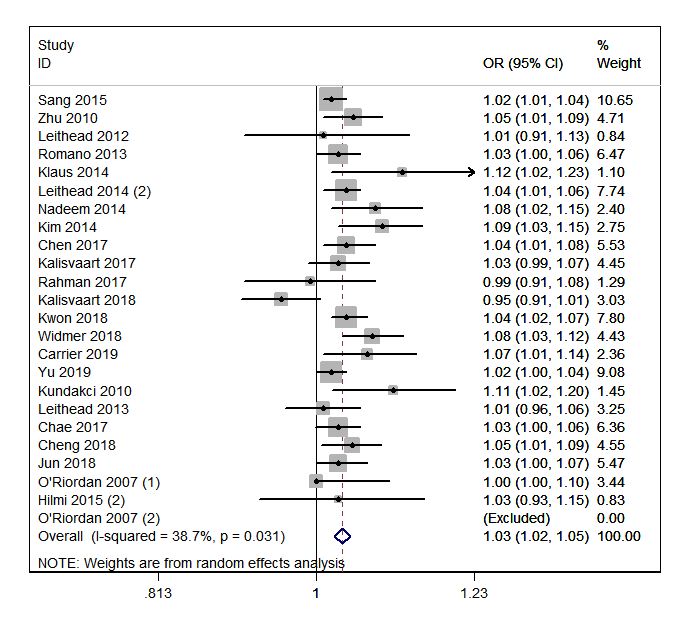


High MELD score


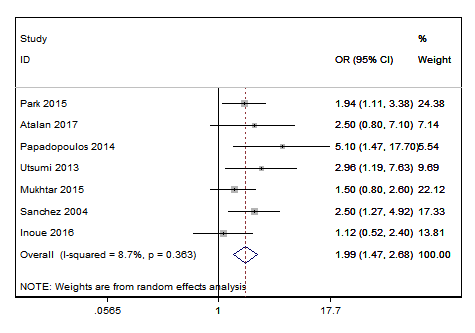


Preoperative eGFR (per ml/min/1.73m^2^)


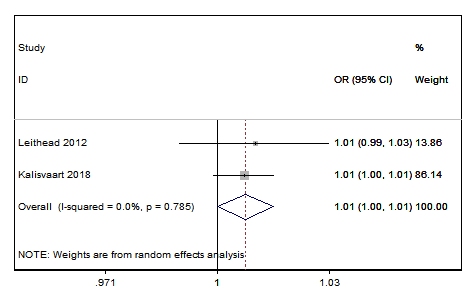


APACHE Ⅱ(per score)


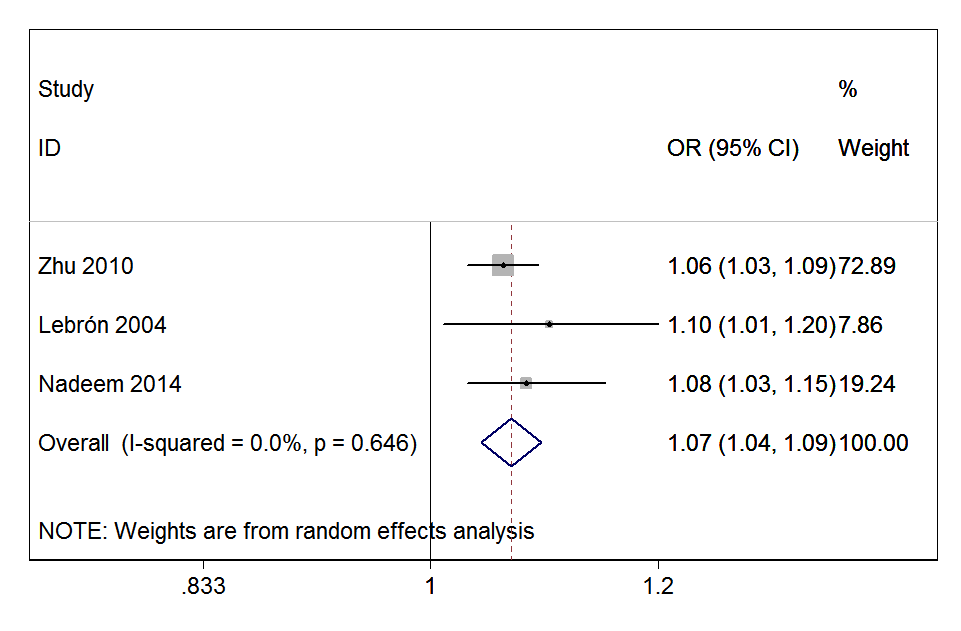


Preoperative serum creatinine (per μmol/L)


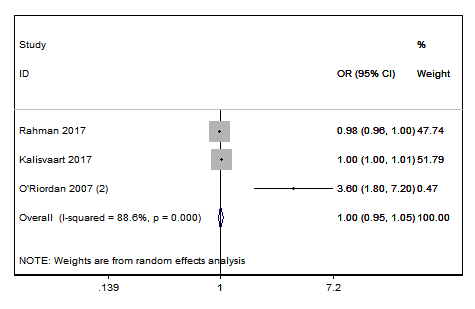


Preoperative serum creatinine (per mg/dL)


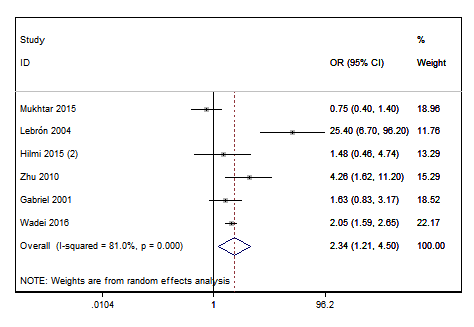


High preoperative serum creatinine


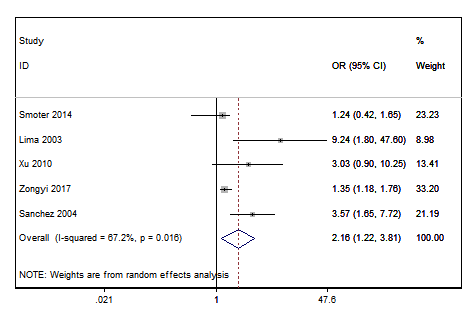


Preoperative serum albumin (per g/dL)


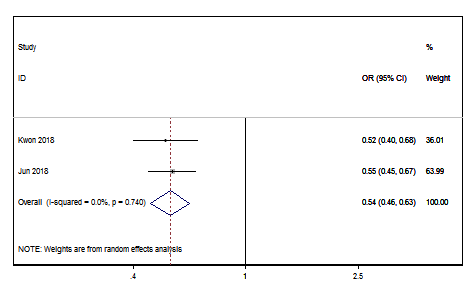


Preoperative hypoalbuminemia


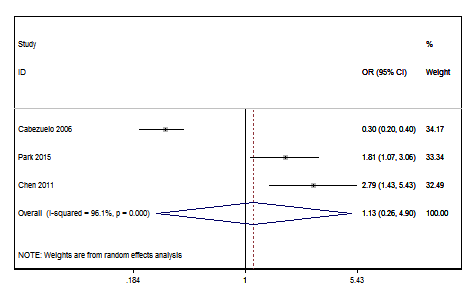


Preoperative hemoglobin (per g/dL)


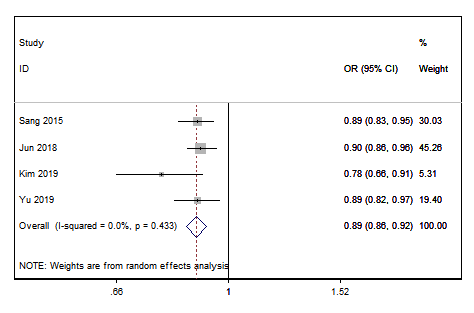


Preoperative anemia


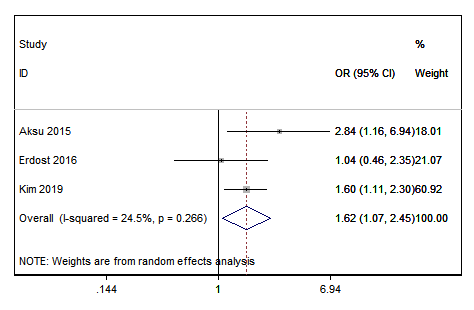


Cadaveric donor liver graft


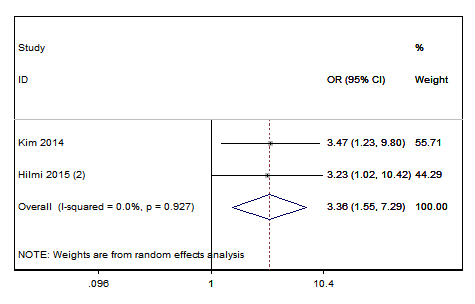


DCD organ


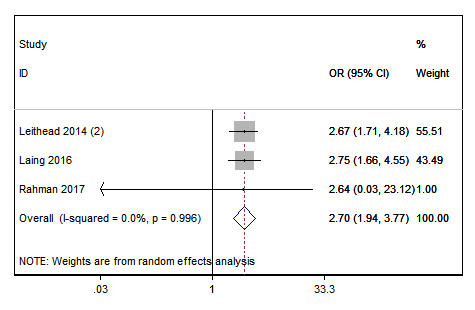


Donor age (per year)


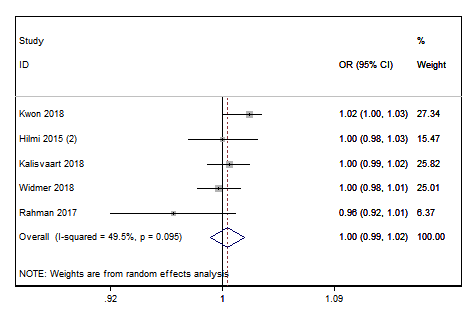


Older donor age


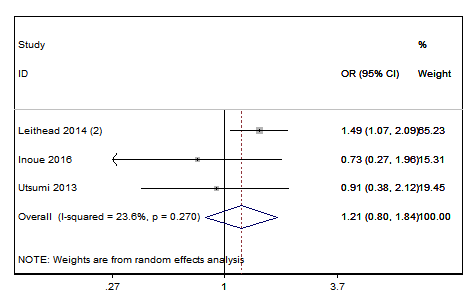


Donor BMI ≥ 30 kg/m^2^

^
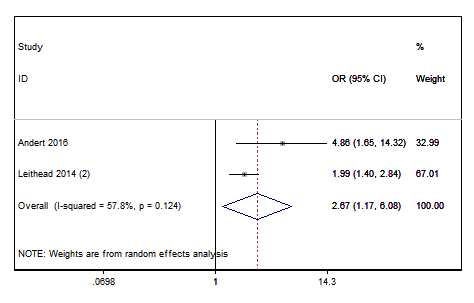
^

Donor risk index (per point)


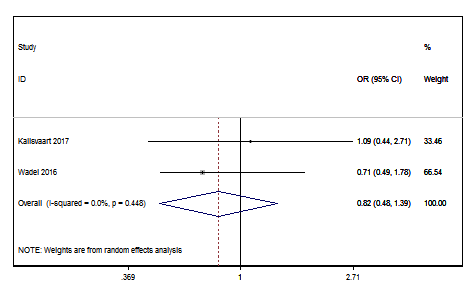


ABO-incompatible liver transplantation


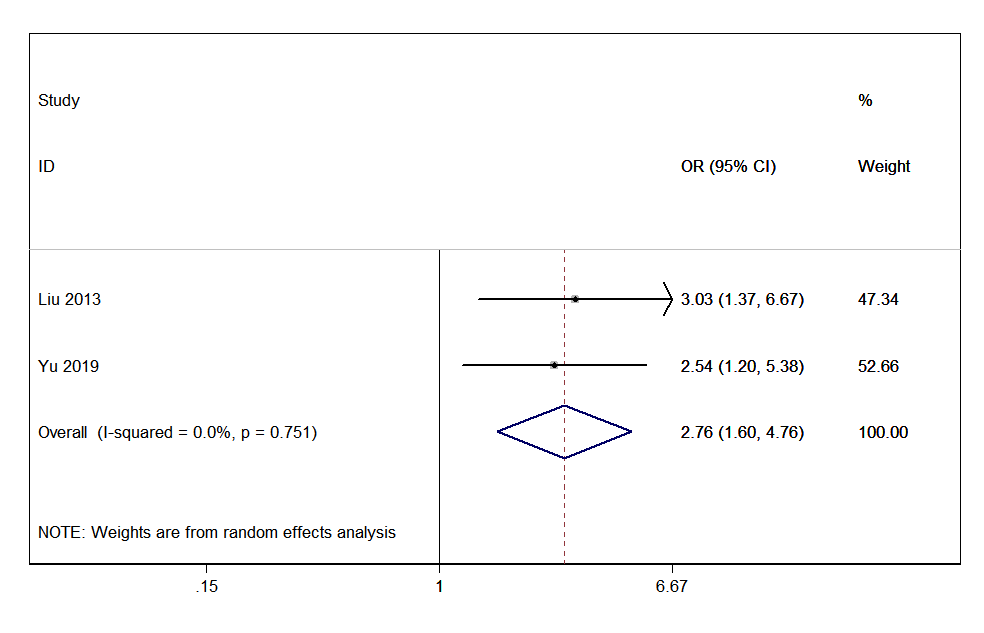


Graft-recipient weight ratio


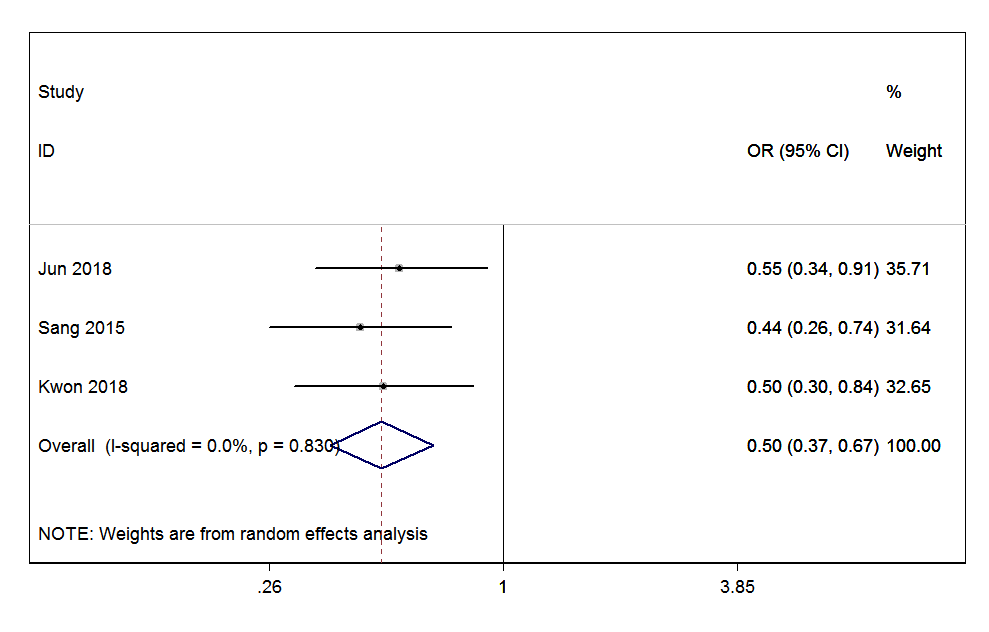


Low graft to recipient body weight ratio


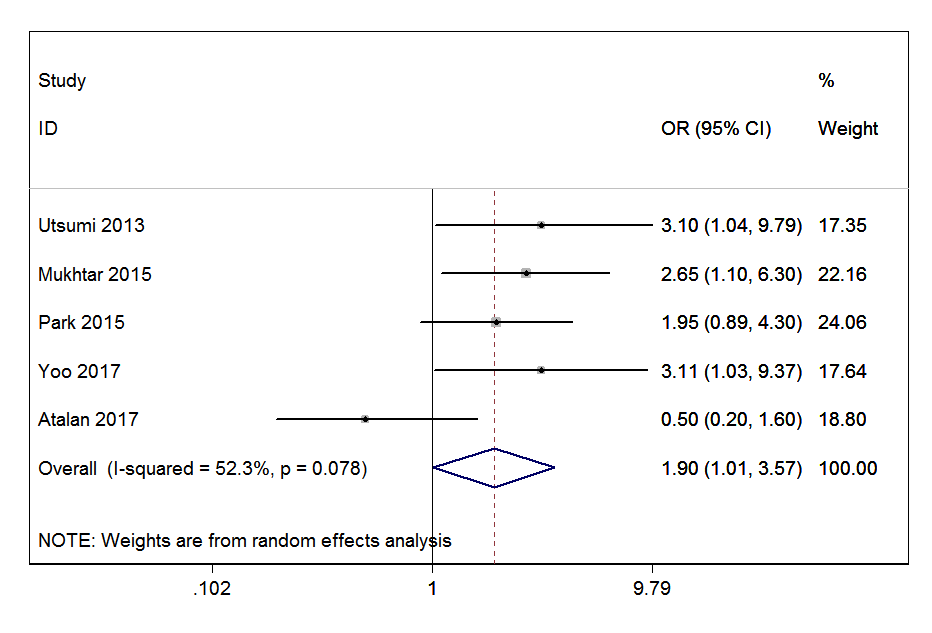


Cold ischaemic time (per min)


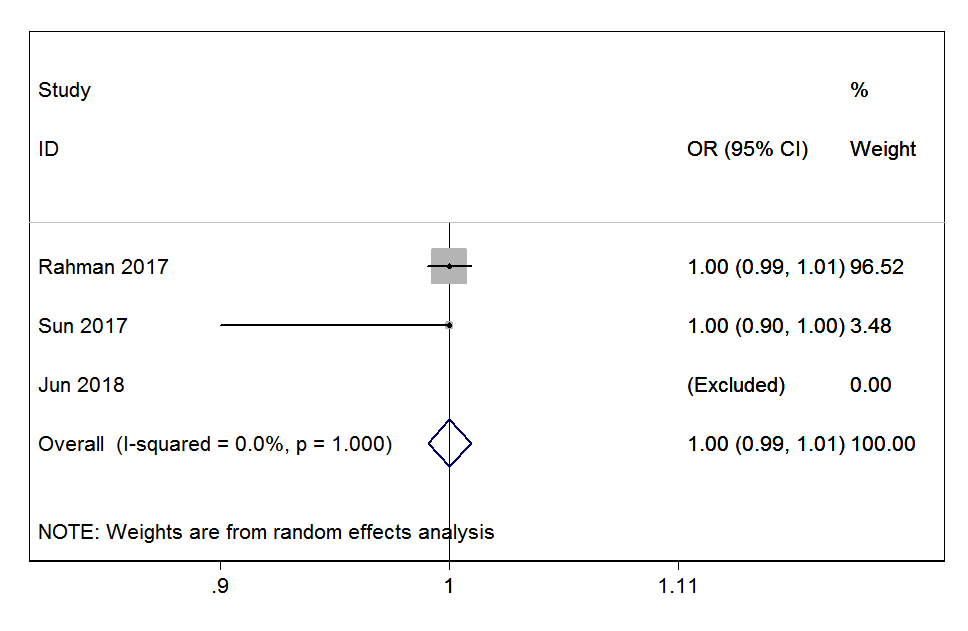


Cold ischaemic time (per hour)


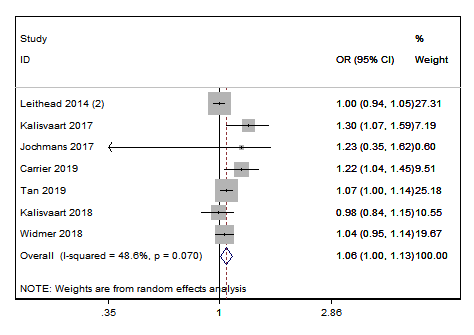


Long cold ischaemic time


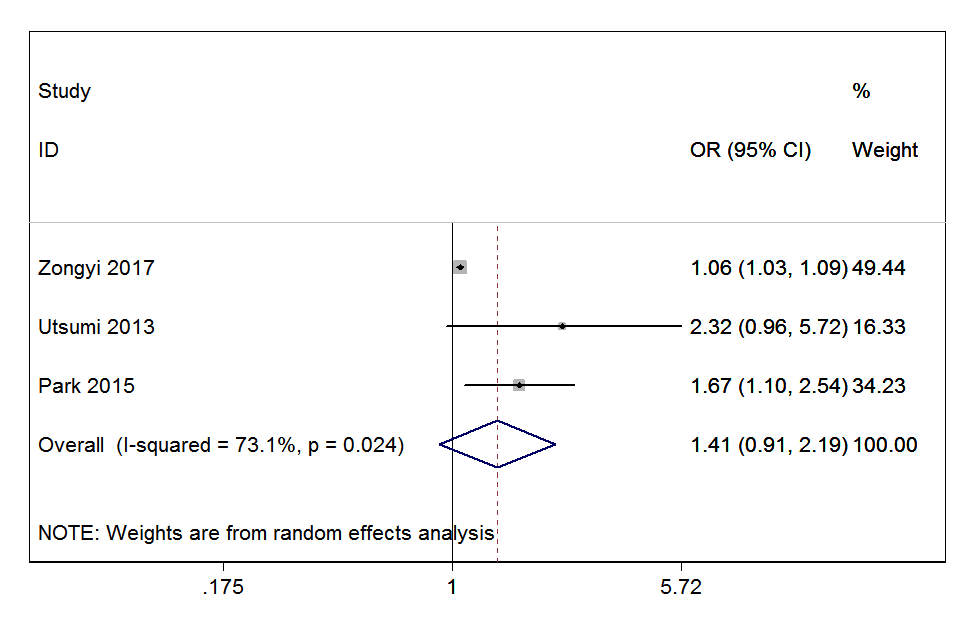


Warm ischaemic time (per min)


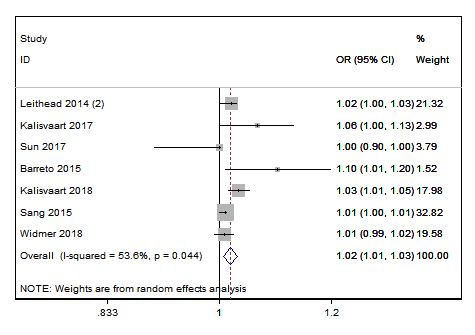


Long warm ischaemic time


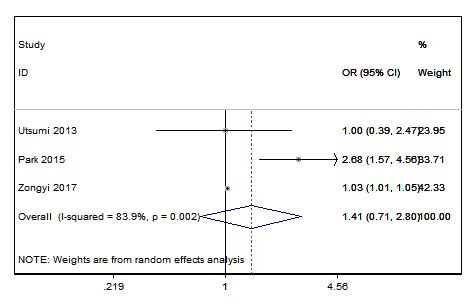


Piggyback surgical technique


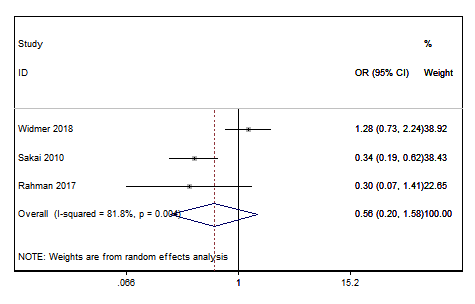


Split liver transplantation


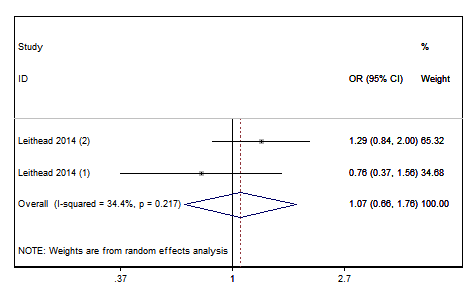


Venovenous bypass


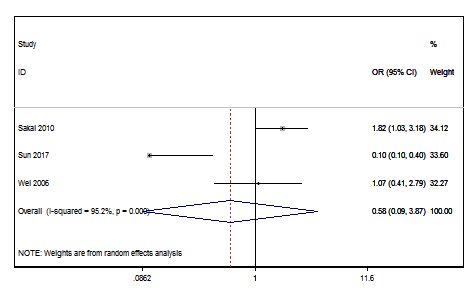


Intraoperative hypotension


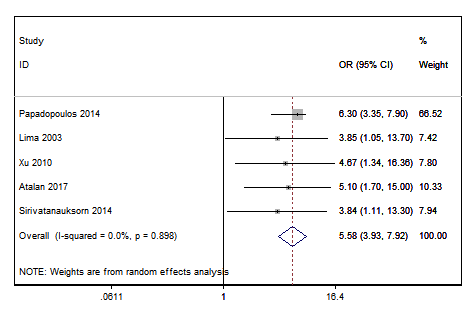


Intraoperative blood loss (per liter)


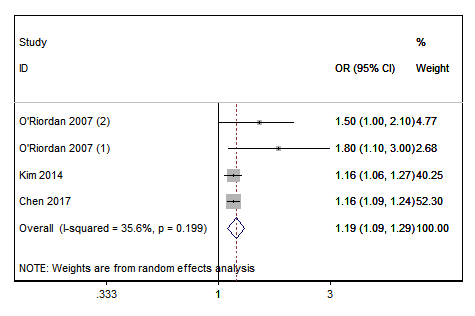


Large intraoperative blood loss


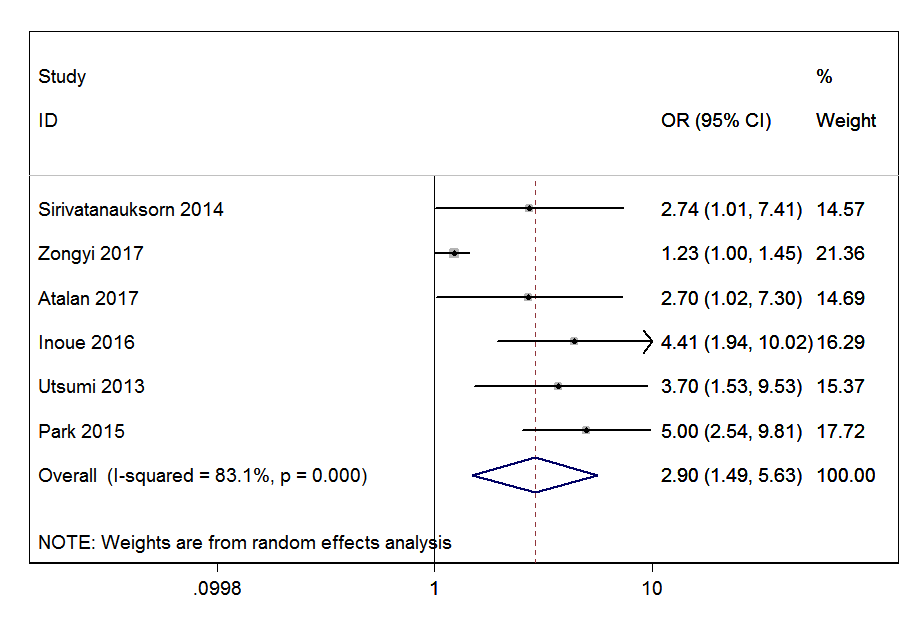


Intraoperative use of vasopressor


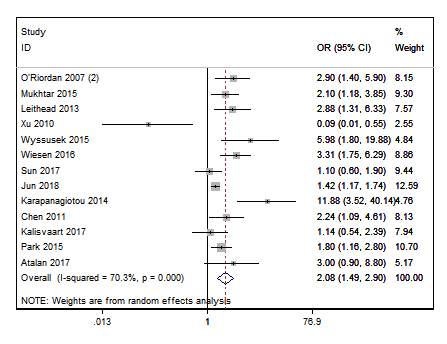


Intraoperative colloidal use


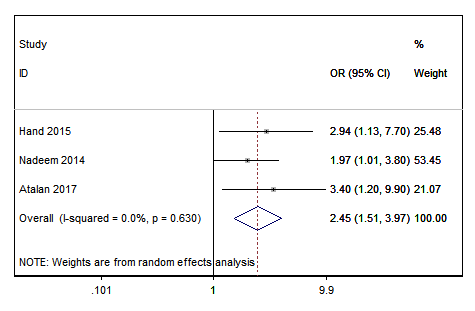


Intraoperative RBC transfusion (per unit)


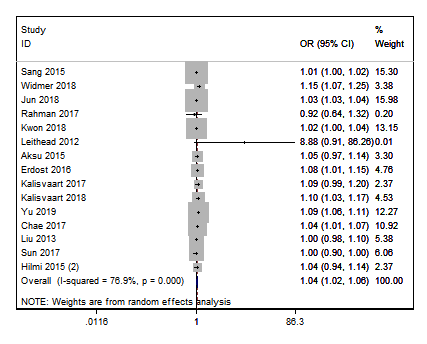


Intraoperative RBC transfusion (per liter)


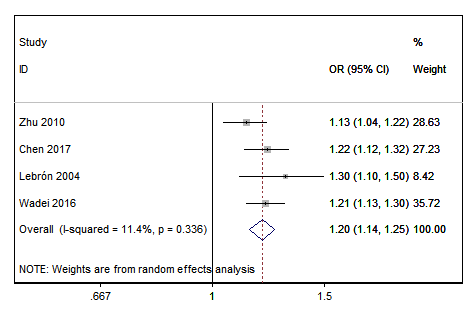


Large intraoperative RBC transfusion


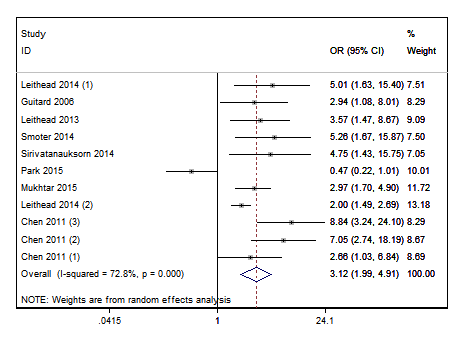


Intraoperative FFP transfusion (per unit)


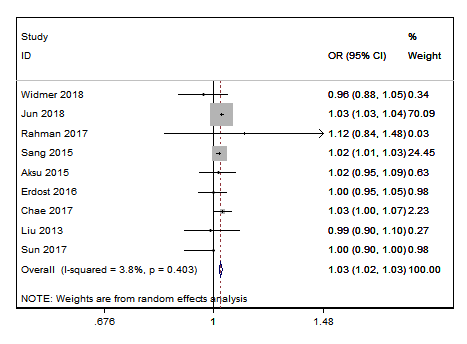


Intraoperative platelet transfusion (per unit)


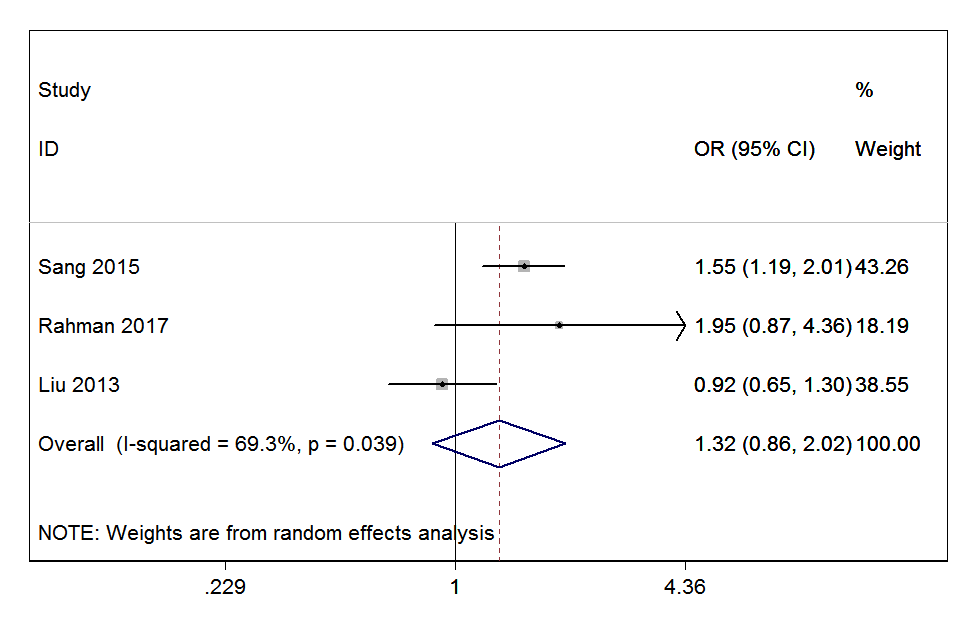


Intraoperative urine output (per mL)


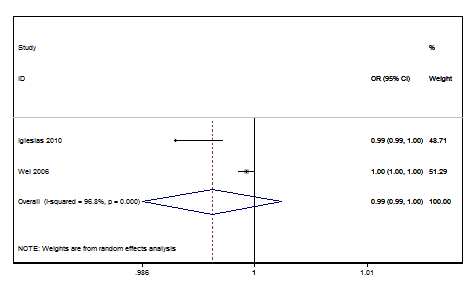


Postreperfusion syndrome


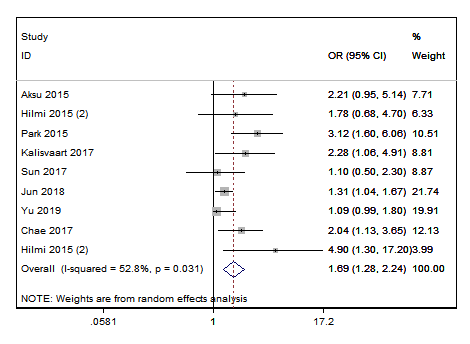


Duration of operation (per hour)


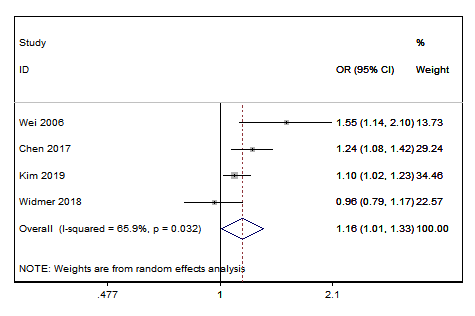


Long operation time


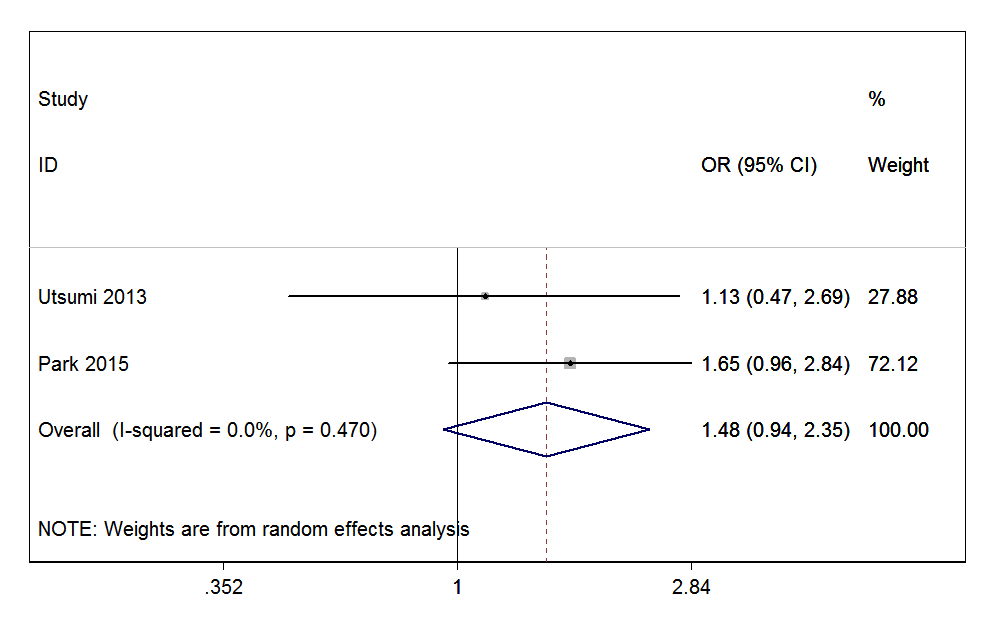


Postoperative hypotension


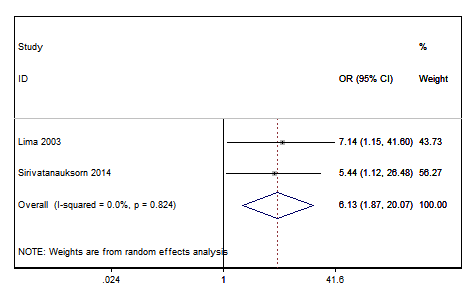


Large postoperative RBC transfusion


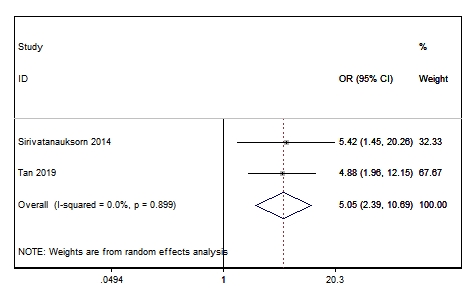


Postoperative use of vasopressor


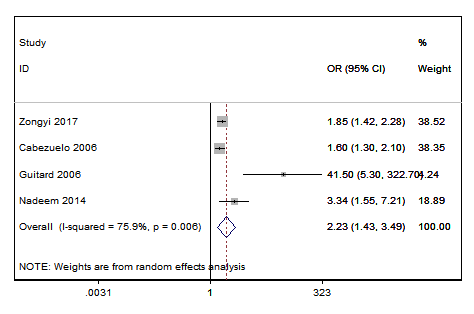


Postoperative peak AST (per u/L)


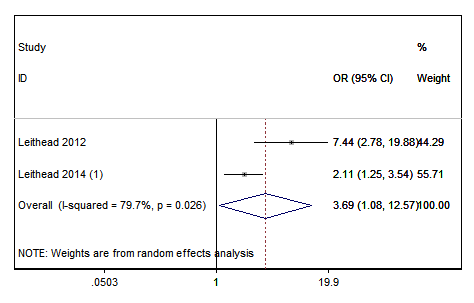


Postoperative peak AST (per IU/L)


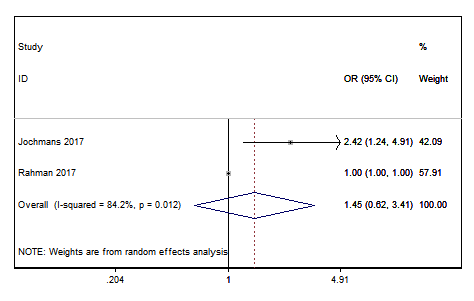


Overexposure to CNI


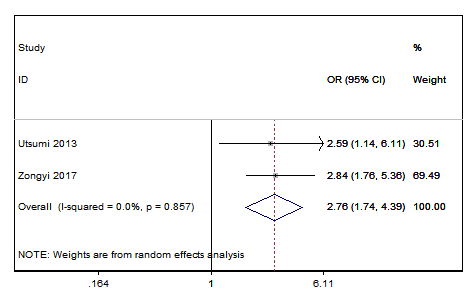


No combined use of mycophenolate mofetil


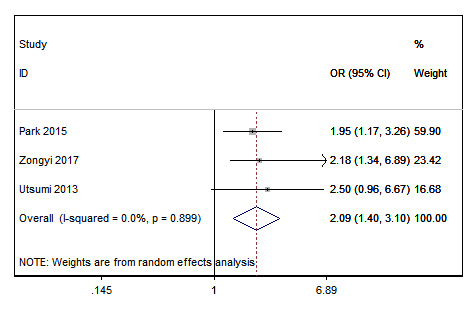


Postoperative tacrolimus peak level (per ug/L)


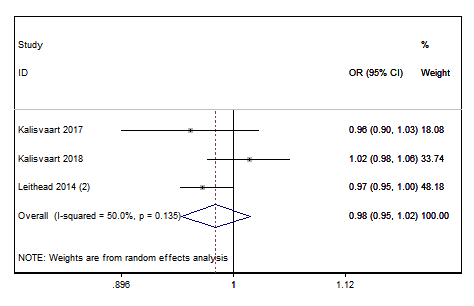


Postoperative tacrolimus use


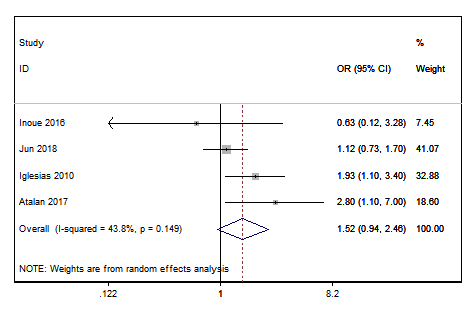


Postoperative hypoalbuminemia


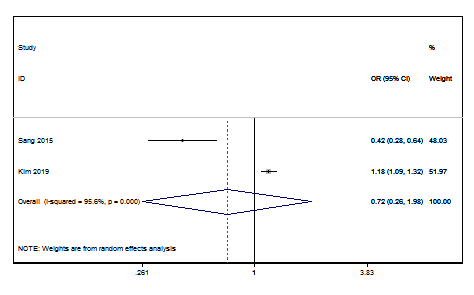


Graft dysfunction


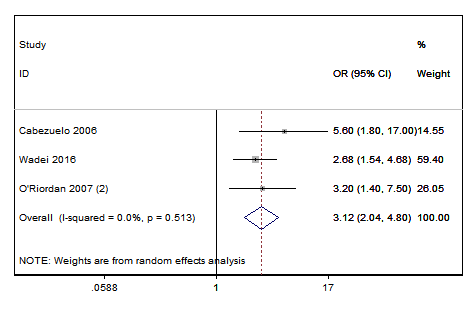


Infection


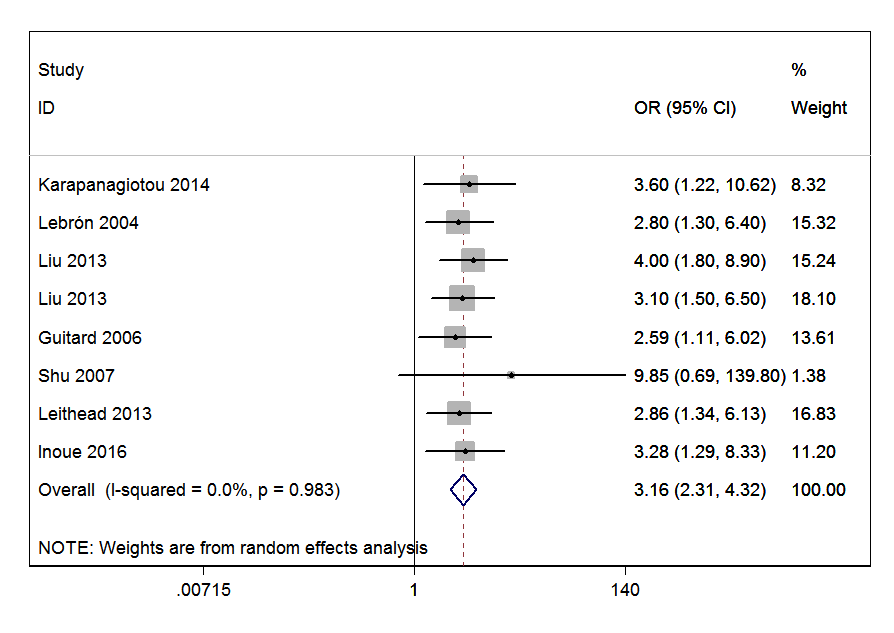


**Supplementary 7: Results of sensitivity analysis after excluding 1 single study**

Age (per year) (heterogeneity: I^2^ = 78.3%, P for Chi^2^ test = 0.000)


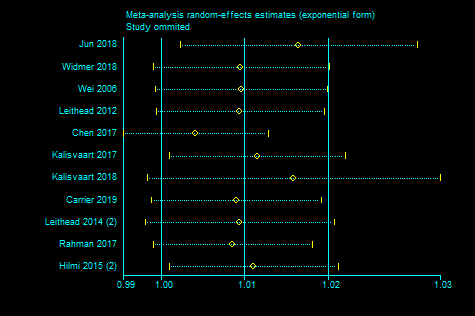


Female gender (heterogeneity: I^2^ = 42.5%, P for Chi^2^ test = 0.042)


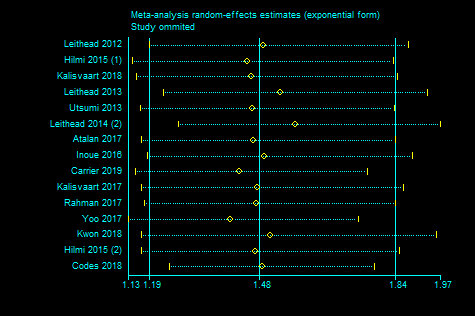


Overweight (heterogeneity: I^2^ = 56.4%, P for Chi^2^ test = 0.057)


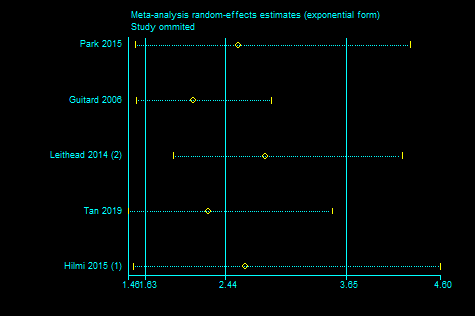


Hepatocellular carcinoma (heterogeneity: I^2^ = 66.6%, P for Chi^2^ test = 0.030)


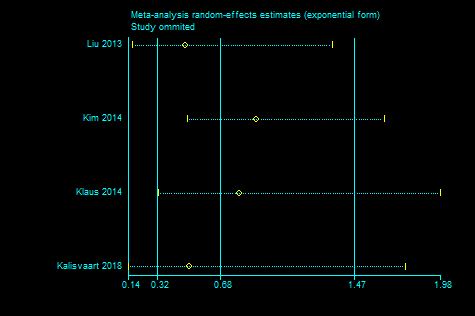


Preoperative hypertension (heterogeneity: I^2^ = 64.7%, P for Chi^2^ test = 0.003)


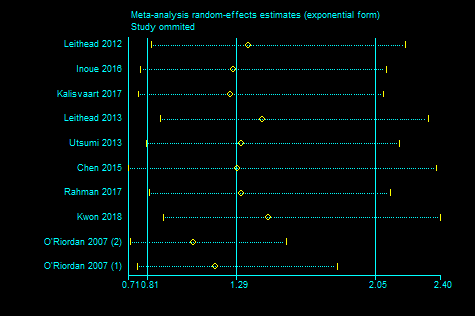


Child-Turcotte-Pugh grade C (heterogeneity: I^2^ = 76.7%, P for Chi^2^ test = 0.005)


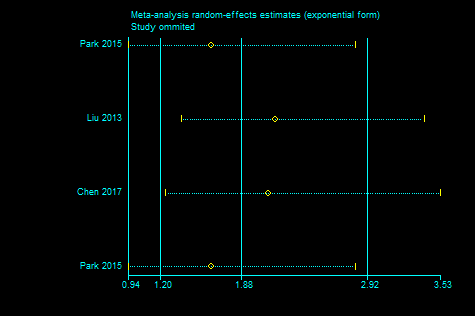


Child-Turcotte-Pugh score (per score) (heterogeneity: I^2^ = 73.2%, P for Chi^2^ test = 0.005)


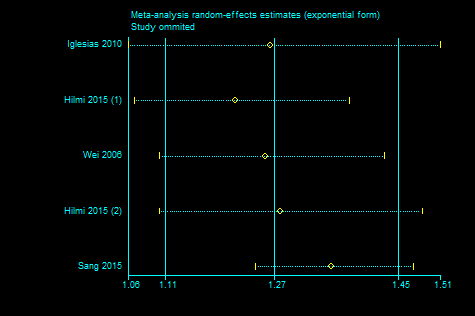


MELD (per score) (heterogeneity: I^2^ = 38.7%, P for Chi^2^ test = 0.031)


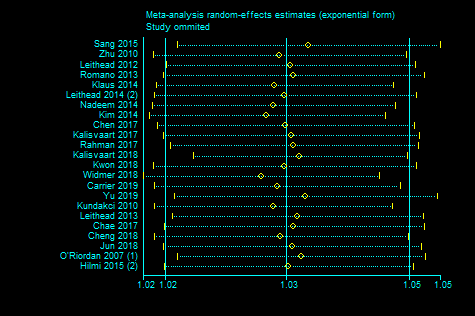


Preoperative serum creatinine (μmol/L) (heterogeneity: I^2^ = 88.6%, P for Chi^2^ test = 0.000)


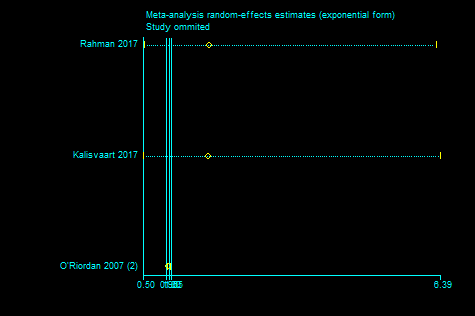


Preoperative serum creatinine (per mg/dL) (heterogeneity: I^2^ = 81.0%, P for Chi^2^ test = 0.000)


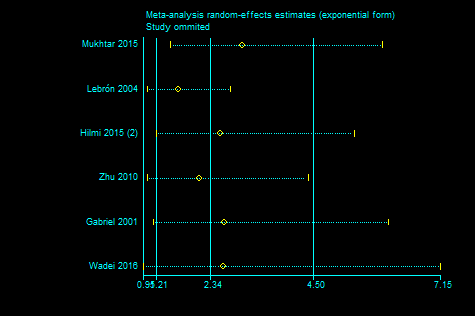


High preoperative serum creatinine (heterogeneity: I^2^ = 67.2%, P for Chi^2^ test = 0.016)


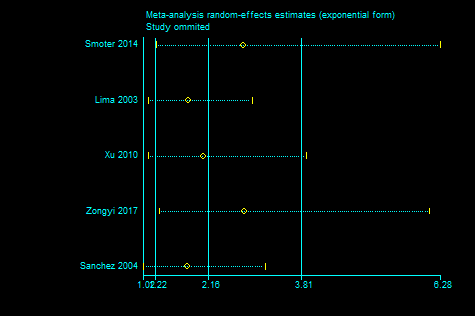


Preoperative hypoalbuminemia (heterogeneity: I^2^ = 96.1%, P for Chi^2^ test = 0.000)


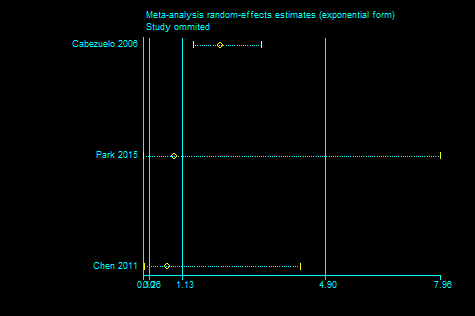


Donor age (per year) (heterogeneity: I^2^ = 49.5%, P for Chi^2^ test = 0.095)


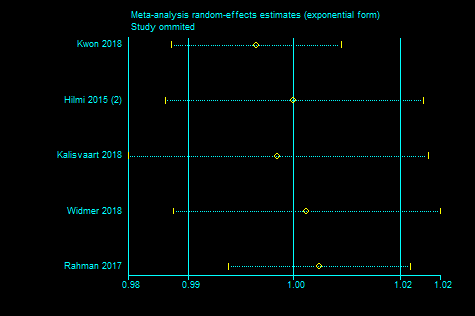


Low graft to recipient body weight ratio (heterogeneity: I^2^ = 52.3%, P for Chi^2^ test = 0.078)


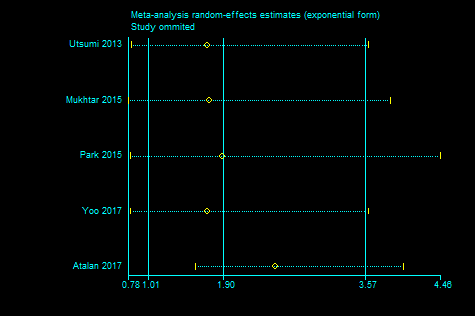


Cold ischaemic time (per hour) (heterogeneity: I^2^ = 48.6%, P for Chi^2^ test = 0.070)


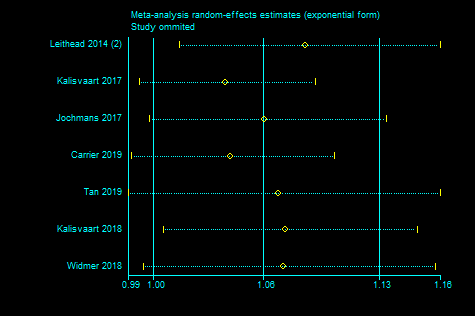


Long cold ischaemic time (heterogeneity: I^2^ = 73.1%, P for Chi^2^ test = 0.024)


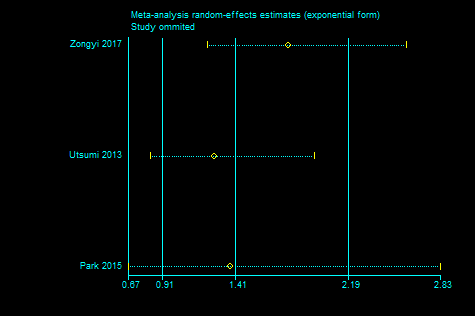


Warm ischaemic time (per min) (heterogeneity: I^2^ = 53.6%, P for Chi^2^ test = 0.044)


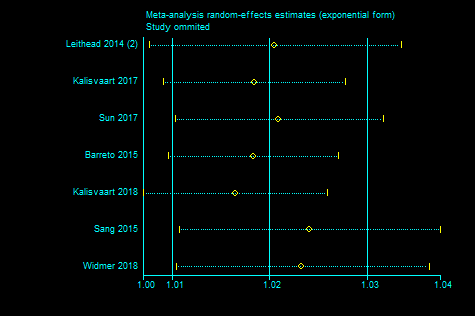


Long warm ischaemic time (heterogeneity: I^2^ = 83.9%, P for Chi^2^ test = 0.002)


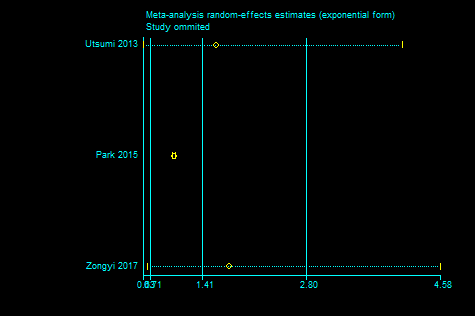


Piggyback surgical technique (heterogeneity: I^2^ = 81.8%, P for Chi^2^ test = 0.004)


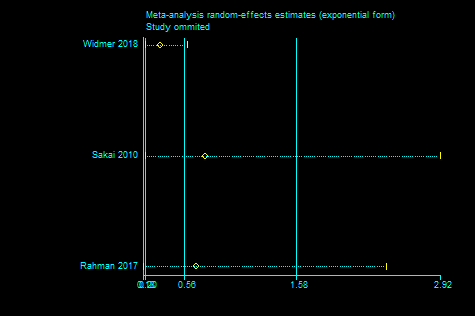


Venovenous bypass (heterogeneity: I^2^ = 95.2%, P for Chi^2^ test = 0.000)


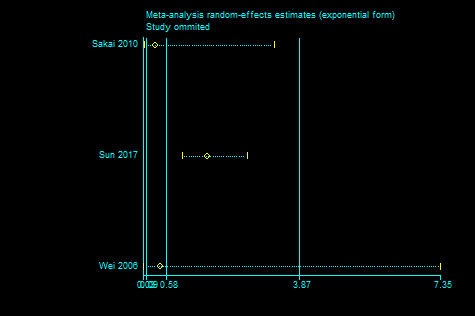


Large intraoperative blood loss (heterogeneity: I^2^ = 83.1%, P for Chi^2^ test = 0.000)


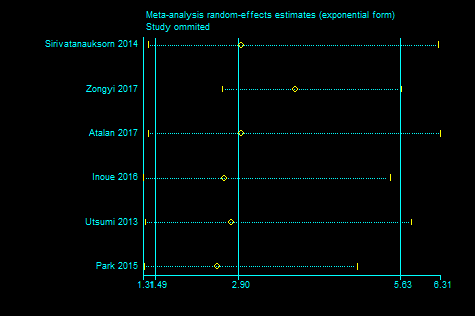


Intraoperative use of vasopressor (heterogeneity: I^2^ = 70.3%, P for Chi^2^ test = 0.000)


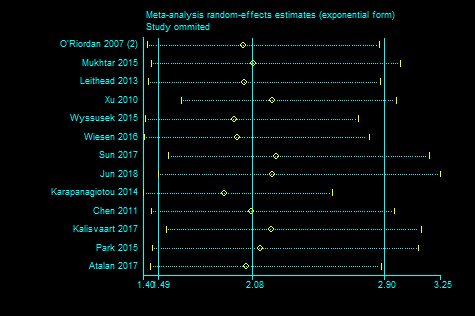


Intraoperative RBC transfusion (per unit) (heterogeneity: I^2^ = 76.9%, P for Chi^2^ test = 0.000)


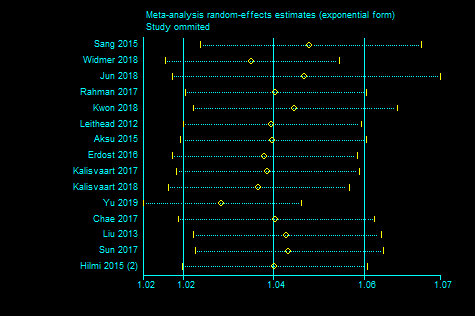


Large intraoperative RBC transfusion (heterogeneity: I^2^ = 72.8%, P for Chi^2^ test = 0.000)


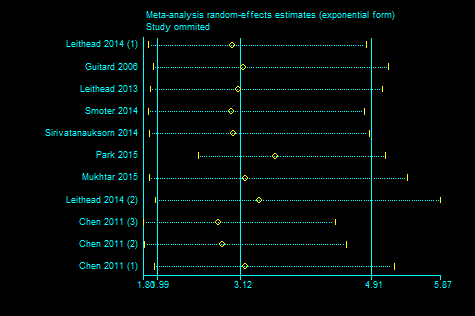


Intraoperative platelet transfusion (per unit) (heterogeneity: I^2^ = 69.3%, P for Chi^2^ test = 0.039)


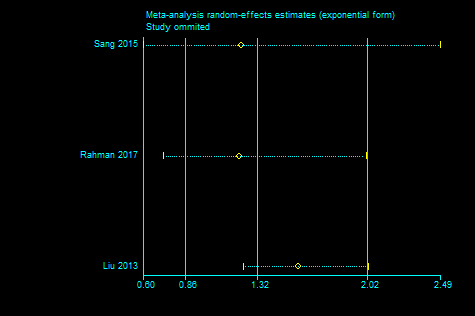


Postreperfusion syndrome (heterogeneity: I^2^ = 52.8%, P for Chi^2^ test = 0.031)


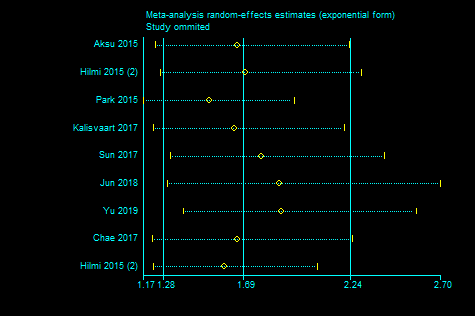


Duration of operation (per hour) (heterogeneity: I^2^ = 65.9%, P for Chi^2^ test = 0.032)

Postoperative use of vasopressor (heterogeneity: I^2^ = 75.9%, P for Chi^2^ test = 0.006)

**Supplementary 8: Factors with a significant change of I^2^ (I^2^ change > 30%) after conducting sensitivity analysis**

| Factor | Result | OR (95% CI) | Heterogeneity | | P Value | Possible source of heterogeneity | A or B is more credible |
| --- | --- | --- | --- | --- | --- | --- | --- |
|  |  |  | I^2^ (%) | Chi^2^ |  |  |  |
| Overweight | Primary result | 2.437 (1.629 - 3.646) | 56.4 | 0.057 | 0.000 |  |  |
|  | Result after excluding Leithead 2014 (2) | 2.838 (1.901 - 4.208) | 21.1 | 0.283 | 0.000 | A relatively high cutoff point of overweight | B |
| Child score (per score) | Primary result | 1.272 (1.115 - 1.452) | 73.2 | 0.005 | 0.000 |  |  |
|  | Result after excluding Sang 2015 | 1.355 (1.246 - 1.474) | 0.0 | 0.587 | 0.000 | Insufficient adjustment of confounding factors | B |
| Preoperative hypoalbuminemia | Primary result | 1.127 (0.259 - 4.905) | 96.1 | 0.000 | 0.874 |  |  |
|  | Result after excluding Cabezuelo 2006 | 2.134 (1.412 - 3.225) | 0.0 | 0.321 | 0.000 | A relatively long duration of evaluation | B |
| Donor age (per year) | Primary result | 1.004 (0.991 - 1.017) | 49.5 | 0.095 | 0.578 |  |  |
|  | Result after excluding Kwon 2018 | 0.999 (0.989 - 1.009) | 0.0 | 0.416 | 0.870 | Insufficient adjustment of confounding factors | B |
| Low graft to recipient body weight ratio | Primary result | 1.902 (1.013 - 3.568) | 52.3 | 0.078 | 0.045 |  |  |
|  | Result after excluding Atalan 2017 | 2.514 (1.573 - 4.020) | 0.0 | 0.875 | 0.000 | A relatively high cutoff point of graft to recipient body weight ratio | B |
| Long cold ischaemic time | Primary result | 1.408 (0.907 - 2.187) | 73.1 | 0.024 | 0.128 |  |  |
|  | Result after excluding Zongyi 2017 | 1.772 (1.213 - 2.588) | 0.0 | 0.513 | 0.003 | A relatively high cutoff point of long cold ischaemic time | B |
| Long warm ischaemic time | Primary result | 1.411 (0.711 - 2.799) | 83.9 | 0.002 | 0.325 |  |  |
|  | Result after excluding  Park 2015 | 1.028 (1.011 - 1.046) | 0.0 | 0.953 | 0.001 | Not found | A |
| Piggyback surgical technique | Primary result | 0.556 (0.195 - 1.585) | 81.8 | 0.004 | 0.272 |  |  |
|  | Result after excluding Widmer 2018 | 0.337 (0.194 - 0.586) | 0.0 | 0.888 | 0.000 | Not found | A |
| Venovenous bypass | Primary result | 0.577 (0.086 - 3.865) | 95.2 | 0.000 | 0.571 |  |  |
|  | Result after excluding Sun 2017 | 1.585 (0.976 - 2.575) | 0.0 | 0.350 | 0.063 | Adjusted odds ratio | B |
| Large intraoperative blood loss | Primary result | 2.900 (1.495 - 5.627) | 83.1 | 0.000 | 0.002 |  |  |
|  | Result after excluding Zongyi 2017 | 3.859 (2.638 - 5.646) | 0.0 | 0.803 | 0.000 | A relatively long duration of evaluation | B |
| Intraoperative platelet transfusion (per unit) | Primary result | 1.321 (0.863 - 2.024) | 69.3 | 0.039 | 0.200 |  |  |
|  | Result after excluding Liu 2013 | 1.584 (1.235 - 2.033) | 0.0 | 0.597 | 0.000 | Different diagnostic criteria | B |
| Postoperative use of vasopressor | Primary result | 2.234 (1.431 - 3.488) | 75.9 | 0.006 | 0.000 |  |  |
|  | Result after excluding Guitard 2006 | 1.827 (1.435 - 2.327) | 42.0 | 0.178 | 0.000 | A relatively small sample size | B |

**Note:** OR, odds ratio; CI, confidence interval; A, the primary result; B, the result after excluding one literature.

**Supplementary 9: Subgroup analysis for factors with evident heterogeneity by diagnostic criteria of AKI**

| Factor | No. of study | Sample | OR (95% CI) | Heterogeneity | | P Value |
| --- | --- | --- | --- | --- | --- | --- |
|  |  |  |  | I^2^ | Chi^2^ |  |
| Age (per year) | 11 | 5476 | 1.006 (0.998 - 1.015) | 78.3 | 0.000 | 0.155 |
| RIFLE | 3 | 2108 | 0.990 (0.985 - 0.995) | 0.0 | 0.407 | 0.000 |
| AKIN | 2 | 682 | 1.064 (0.994 - 1.139) | 57.7 | 0.124 | 0.072 |
| KDIGO | 4 | 2425 | 1.006 (0.996 - 1.015) | 59.4 | 0.061 | 0.267 |
| Other | 2 | 261 | 0.996 (0.953 - 1.041) | 47.6 | 0.167 | 0.860 |
| Female gender | 15 | 5399 | 1.479 (1.186 - 1.845) | 42.5 | 0.042 | 0.001 |
| RIFLE | 5 | 1045 | 1.572 (1.067 - 2.315) | 28.2 | 0.233 | 0.022 |
| AKIN | 1 | 116 | 2.326 (0.385 - 14.042) | - | - | 0.357 |
| KDIGO | 6 | 3524 | 1.357 (0.909 - 2.027) | 66.3 | 0.011 | 0.135 |
| Other | 3 | 714 | 1.669 (1.176 - 2.370) | 0.0 | 0.764 | 0.004 |
| Overweight | 5 | 2420 | 2.437 (1.629 - 3.646) | 56.4 | 0.057 | 0.000 |
| RIFLE | 2 | 617 | 3.919 (1.135 - 13.531) | 65.6 | 0.088 | 0.031 |
| KDIGO | 2 | 1379 | 2.184 (1.062 - 4.490) | 72.8 | 0.055 | 0.034 |
| Other | 1 | 424 | 2.300 (1.371 - 3.858) | - | - | 0.002 |
| Hepatocellular carcinoma | 4 | 1027 | 0.681 (0.316 - 1.469) | 66.6 | 0.030 | 0.328 |
| RIFLE | 1 | 389 | 1.400 (0.683 - 2.870) | - | - | 0.358 |
| KDIGO | 1 | 368 | 1.001 (0.552 - 1.815) | - | - | 0.997 |
| Other | 2 | 270 | 0.278 (0.083 - 0.934) | 37.0 | 0.208 | 0.038 |
| Preoperative hypertension | 10 | 5544 | 1.291 (0.814 - 2.045) | 64.7 | 0.003 | 0.278 |
| RIFLE | 6 | 1312 | 1.562 (0.686 - 3.558) | 67.8 | 0.008 | 0.288 |
| AKIN | 1 | 116 | 0.593 (0.042 - 8.407) | - | - | 0.699 |
| KDIGO | 1 | 1136 | 0.720 (0.490 - 1.059) | - | - | 0.095 |
| Other | 2 | 2980 | 1.360 (0.987 - 1.874) | 0.0 | 0.890 | 0.060 |
| Child-Turcotte-Pugh grade C | 4 | 2031 | 1.876 (1.205 - 2.922) | 76.7 | 0.005 | 0.005 |
| RIFLE | 3 | 1465 | 2.102 (1.252 - 3.531) | 70.7 | 0.033 | 0.005 |
| AKIN | 1 | 566 | 1.430 (1.047 - 1.954) | - | - | 0.025 |
| Child-Turcotte-Pugh score (per score) | 5 | 2371 | 1.272 (1.115 - 1.452) | 73.2 | 0.005 | 0.000 |
| AKIN | 2 | 1686 | 1.202 (1.042 - 1.386) | 76.7 | 0.039 | 0.012 |
| Other | 3 | 685 | 1.389 (1.243 - 1.552) | 0.0 | 0.423 | 0.000 |
| MELD (per score) | 23 | 10444 | 1.035 (1.024 - 1.045) | 38.7 | 0.031 | 0.000 |
| RIFLE | 8 | 3247 | 1.032 (1.009 - 1.055) | 22.1 | 0.261 | 0.005 |
| AKIN | 5 | 2207 | 1.026 (1.014 - 1.038) | 0.0 | 0.510 | 0.000 |
| KDIGO | 7 | 4456 | 1.035 (1.014 - 1.057) | 64.8 | 0.009 | 0.001 |
| Other | 4 | 534 | 1.060 (1.017 - 1.104) | 43.8 | 0.148 | 0.006 |
| Preoperative serum creatinine (μmol/L) | 3 | 582 | 0.998 (0.952 - 1.046) | 88.6 | 0.000 | 0.931 |
| RIFLE | 2 | 466 | 1.810 (0.520 - 6.304) | 92.3 | 0.000 | 0.351 |
| AKIN | 1 | 116 | 0.979 (0.958 - 1.000) | - | - | 0.052 |
| Preoperative serum creatinine (per mg/dL) | 6 | 2422 | 2.337 (1.215 - 4.497) | 81.0 | 0.000 | 0.011 |
| AKIN | 3 | 1745 | 1.772 (0.793 - 3.962) | 82.5 | 0.003 | 0.163 |
| Other | 3 | 677 | 3.675 (0.754 - 17.924) | 85.7 | 0.001 | 0.107 |
| High preoperative serum creatinine | 5 | 5498 | 2.155 (1.219 - 3.811) | 67.2 | 0.016 | 0.008 |
| RIFLE | 1 | 4482 | 1.352 (1.107 - 1.652) | - | - | 0.003 |
| Other | 4 | 1016 | 2.800 (1.289 - 6.080) | 59.4 | 0.060 | 0.009 |
| Preoperative hypoalbuminemia | 3 | 958 | 1.127 (0.259 - 4.905) | 96.1 | 0.000 | 0.874 |
| RIFLE | 2 | 774 | 2.134 (1.412 - 3.225) | 0.0 | 0.321 | 0.000 |
| Other | 1 | 184 | 0.300 (0.212 - 0.424) | - | - | 0.000 |
| Donor age (per year) | 5 | 2170 | 1.004 (0.991 - 1.017) | 49.5 | 0.095 | 0.578 |
| AKIN | 1 | 116 | 0.964 (0.920 - 1.010) | - | - | 0.124 |
| KDIGO | 3 | 1882 | 1.008 (0.994 - 1.021) | 53.3 | 0.117 | 0.258 |
| Other | 1 | 172 | 1.000 (0.974 - 1.026) | - | - | 1.000 |
| Low graft to recipient body weight ratio | 5 | 1565 | 1.902 (1.013 - 3.568) | 52.3 | 0.078 | 0.045 |
| RIFLE | 3 | 1042 | 2.461 (1.411 - 4.294) | 0.0 | 0.715 | 0.002 |
| AKIN | 1 | 303 | 2.650 (1.107 - 6.342) | - | - | 0.029 |
| KDIGO | 1 | 220 | 0.500 (0.177 - 1.414) | - | - | 0.191 |
| Cold ischaemic time (per hour) | 7 | 2887 | 1.064 (1.003 - 1.130) | 48.6 | 0.070 | 0.041 |
| RIFLE | 2 | 235 | 1.297 (1.070 - 1.573) | 0.0 | 0.888 | 0.008 |
| KDIGO | 5 | 2652 | 1.044 (0.991 - 1.099) | 39.7 | 0.156 | 0.105 |
| Warm ischaemic time (per min) | 7 | 3427 | 1.018 (1.007 - 1.029) | 53.6 | 0.044 | 0.001 |
| RIFLE | 1 | 155 | 1.064 (1.002 - 1.130) | - | - | 0.043 |
| AKIN | 3 | 1374 | 1.019 (0.984 - 1.055) | 48.7 | 0.142 | 0.299 |
| KDIGO | 3 | 1898 | 1.020 (1.007 - 1.033) | 44.8 | 0.163 | 0.003 |
| Large intraoperative blood loss | 6 | 5639 | 2.900 (1.495 - 5.627) | 83.1 | 0.000 | 0.002 |
| RIFLE | 3 | 5220 | 2.698 (0.949 - 7.671) | 89.9 | 0.000 | 0.063 |
| KDIGO | 1 | 220 | 2.700 (1.009 - 7.223) | - | - | 0.048 |
| Other | 2 | 199 | 3.636 (1.929 - 6.854) | 0.0 | 0.471 | 0.000 |
| Intraoperative use of vasopressor | 13 | 4625 | 2.079 (1.492 - 2.899) | 70.3 | 0.000 | 0.000 |
| RIFLE | 7 | 3474 | 2.130 (1.452 - 3.127) | 67.4 | 0.005 | 0.000 |
| AKIN | 2 | 545 | 1.515 (0.804 - 2.854) | 57.6 | 0.125 | 0.199 |
| KDIGO | 1 | 220 | 3.000 (0.959 - 9.381) | - | - | 0.059 |
| Other | 3 | 386 | 1.439 (0.224 - 9.218) | 86.8 | 0.001 | 0.701 |
| Intraoperative RBC transfusion (per unit) | 15 | 8006 | 1.042 (1.025 - 1.059) | 76.9 | 0.000 | 0.000 |
| RIFLE | 6 | 3377 | 1.039 (1.008 - 1.070) | 36.1 | 0.166 | 0.012 |
| AKIN | 4 | 1690 | 1.017 (0.998 - 1.037) | 32.2 | 0.219 | 0.080 |
| KDIGO | 4 | 2767 | 1.081 (1.027 - 1.138) | 88.2 | 0.000 | 0.003 |
| Other | 1 | 172 | 1.040 (0.943 - 1.147) | - | - | 0.434 |
| Large intraoperative RBC transfusion | 11 | 3401 | 3.124 (1.986 - 4.914) | 72.8 | 0.000 | 0.000 |
| RIFLE | 6 | 1623 | 2.987 (1.196 - 7.464) | 83.3 | 0.000 | 0.019 |
| AKIN | 1 | 303 | 2.970 (1.749 - 5.042) | - | - | 0.000 |
| KDIGO | 2 | 1296 | 2.680 (1.159 - 6.201) | 58.4 | 0.121 | 0.021 |
| Other | 2 | 179 | 5.017 (2.209 - 11.394) | 0.0 | 0.903 | 0.000 |
| Intraoperative platelet transfusion (per unit) | 3 | 1503 | 1.321 (0.863 - 2.024) | 69.3 | 0.039 | 0.200 |
| RIFLE | 1 | 389 | 0.920 (0.651 - 1.301) | - | - | 0.637 |
| AKIN | 2 | 1114 | 1.584 (1.235 - 2.033) | 0.0 | 0.597 | 0.000 |
| Postreperfusion syndrome | 9 | 4731 | 1.689 (1.275 - 2.236) | 52.8 | 0.031 | 0.000 |
| RIFLE | 4 | 2998 | 1.971 (1.224 - 3.171) | 61.6 | 0.050 | 0.005 |
| AKIN | 2 | 576 | 1.574 (0.868 - 2.852) | 36.4 | 0.210 | 0.135 |
| KDIGO | 1 | 885 | 1.090 (0.808 - 1.470) | - | - | 0.572 |
| Other | 2 | 272 | 2.689 (1.014 - 7.132) | 33.8 | 0.219 | 0.047 |
| Duration of operation (per hour) | 4 | 1563 | 1.158 (1.008 - 1.330) | 65.9 | 0.032 | 0.038 |
| AKIN | 1 | 566 | 1.240 (1.081 - 1.422) | - | - | 0.002 |
| KDIGO | 2 | 908 | 1.058 (0.938 - 1.193) | 33.1 | 0.222 | 0.361 |
| Other | 1 | 89 | 1.547 (1.140 - 2.099) | - | - | 0.005 |
| Postoperative use of vasopressor | 4 | 4903 | 2.234 (1.431 - 3.488) | 75.9 | 0.006 | 0.000 |
| RIFLE | 3 | 4719 | 3.911 (1.357 - 11.274) | 80.9 | 0.005 | 0.012 |
| Other | 1 | 184 | 1.600 (1.259 - 2.034) | - | - | 0.000 |

**Supplementary 10: Subgroup analysis for factors with evident heterogeneity by duration of evaluation**

| Factor | No. of study | Sample | OR (95% CI) | Heterogeneity | | P Value |
| --- | --- | --- | --- | --- | --- | --- |
|  |  |  |  | I^2^ | Chi^2^ |  |
| Age (per year) | 11 | 5476 | 1.006 (0.998 - 1.015) | 78.3 | 0.000 | 0.155 |
| ≤ 7 days | 8 | 4772 | 1.005 (0.995 - 1.014) | 83.4 | 0.000 | 0.333 |
| > 7 days | 2 | 177 | 1.020 (0.987 - 1.055) | 0.0 | 0.556 | 0.236 |
| NA | 1 | 527 | 1.030 (0.986 - 1.076) | - | - | 0.183 |
| Female gender | 15 | 5399 | 1.479 (1.186 - 1.845) | 42.5 | 0.042 | 0.001 |
| ≤ 7 days | 11 | 4286 | 1.474 (1.149 - 1.891) | 47.6 | 0.039 | 0.002 |
| > 7 days | 3 | 586 | 1.173 (0.727 - 1.891) | 0.0 | 0.410 | 0.514 |
| NA | 1 | 527 | 3.030 (1.252 - 7.332) | - | - | 0.014 |
| Overweight | 5 | 2420 | 2.437 (1.629 - 3.646) | 56.4 | 0.057 | 0.000 |
| ≤ 7 days | 3 | 1803 | 2.127 (1.408 - 3.212) | 53.6 | 0.116 | 0.000 |
| > 7 days | 2 | 617 | 3.919 (1.135 - 13.531) | 65.6 | 0.088 | 0.031 |
| Hepatocellular carcinoma | 4 | 1027 | 0.681 (0.316 - 1.469) | 66.6 | 0.030 | 0.328 |
| ≤ 7 days | 1 | 368 | 1.001 (0.552 - 1.815) | - | - | 0.997 |
| > 7 days | 2 | 502 | 0.795 (0.245 - 2.583) | 75.4 | 0.044 | 0.703 |
| NA | 1 | 157 | 0.110 (0.017 - 0.713) | - | - | 0.021 |
| Preoperative hypertension | 10 | 5544 | 1.291 (0.814 - 2.045) | 64.7 | 0.003 | 0.278 |
| ≤ 7 days | 4 | 1525 | 0.841 (0.587 - 1.206) | 1.8 | 0.383 | 0.347 |
| > 7 days | 5 | 1157 | 1.555 (0.552 - 4.381) | 74.3 | 0.004 | 0.403 |
| NA | 1 | 2862 | 1.352 (0.970 - 1.885) | - | - | 0.075 |
| Child-Turcotte-Pugh grade C | 4 | 2031 | 1.876 (1.205 - 2.922) | 76.7 | 0.005 | 0.005 |
| ≤ 7 days | 1 | 566 | 1.430 (1.047 - 1.954) | - | - | 0.025 |
| > 7 days | 3 | 1465 | 2.102 (1.252 - 3.531) | 70.7 | 0.033 | 0.005 |
| Child-Turcotte-Pugh score (per score) | 5 | 2371 | 1.272 (1.115 - 1.452) | 73.2 | 0.005 | 0.000 |
| ≤ 7 days | 4 | 2282 | 1.259 (1.106 - 1.433) | 77.0 | 0.005 | 0.000 |
| > 7 days | 1 | 89 | 2.464 (0.869 - 6.985) | - | - | 0.090 |
| MELD (per score) | 23 | 10444 | 1.035 (1.024 - 1.045) | 38.7 | 0.031 | 0.000 |
| ≤ 7 days | 15 | 8497 | 1.033 (1.022 - 1.044) | 38.0 | 0.062 | 0.000 |
| > 7 days | 6 | 1263 | 1.031 (0.998 - 1.065) | 36.1 | 0.181 | 0.068 |
| NA | 2 | 684 | 1.081 (1.038 - 1.126) | 0.0 | 0.657 | 0.000 |
| Preoperative serum creatinine (μmol/L) | 3 | 582 | 0.998 (0.952 - 1.046) | 88.6 | 0.000 | 0.931 |
| ≤ 7 days | 2 | 271 | 0.993 (0.969 - 1.018) | 77.6 | 0.035 | 0.590 |
| > 7 days | 1 | 311 | 3.600 (1.800 - 7.200) | - | - | 0.000 |
| Preoperative serum creatinine (per mg/dL) | 6 | 2422 | 2.337 (1.215 - 4.497) | 81.0 | 0.000 | 0.011 |
| ≤ 7 days | 3 | 735 | 1.150 (0.669 - 1.977) | 33.2 | 0.224 | 0.614 |
| > 7 days | 3 | 1687 | 5.279 (1.439 - 19.365) | 86.5 | 0.001 | 0.012 |
| High preoperative serum creatinine | 5 | 5498 | 2.155 (1.219 - 3.811) | 67.2 | 0.016 | 0.008 |
| ≤ 7 days | 2 | 200 | 1.670 (0.731 - 3.813) | 36.6 | 0.209 | 0.223 |
| > 7 days | 2 | 4574 | 2.956 (0.464 - 18.807) | 80.8 | 0.022 | 0.251 |
| NA | 1 | 724 | 3.570 (1.650 - 7.722) | - | - | 0.001 |
| Preoperative hypoalbuminemia | 3 | 958 | 1.127 (0.259 - 4.905) | 96.1 | 0.000 | 0.874 |
| ≤ 7 days | 2 | 420 | 0.897 (0.101 - 7.959) | 97.0 | 0.000 | 0.922 |
| > 7 days | 1 | 538 | 1.810 (1.070 - 3.061) | - | - | 0.027 |
| Low graft to recipient body weight ratio | 5 | 1565 | 1.902 (1.013 - 3.568) | 52.3 | 0.078 | 0.045 |
| ≤ 7 days | 3 | 827 | 1.615 (0.528 - 4.939) | 73.2 | 0.024 | 0.400 |
| > 7 days | 2 | 738 | 2.273 (1.193 - 4.330) | 0.0 | 0.507 | 0.013 |
| Cold ischaemic time (per hour) | 7 | 2887 | 1.064 (1.003 - 1.130) | 48.6 | 0.070 | 0.041 |
| ≤ 7 days | 6 | 2360 | 1.046 (0.990 - 1.105) | 38.7 | 0.148 | 0.108 |
| NA | 1 | 527 | 1.220 (1.033 - 1.441) | 48.6 | 0.070 | 0.019 |
| Piggyback surgical technique | 3 | 903 | 0.556 (0.195 - 1.585) | 81.8 | 0.004 | 0.272 |
| ≤ 7 days | 2 | 494 | 0.749 (0.192 - 2.916) | 66.4 | 0.084 | 0.676 |
| NA | 1 | 409 | 0.342 (0.189 - 0.619) | - | - | 0.000 |
| Large intraoperative blood loss | 6 | 5639 | 2.900 (1.495 - 5.627) | 83.1 | 0.000 | 0.002 |
| ≤ 7 days | 3 | 419 | 3.332 (1.956 - 5.677) | 0.0 | 0.681 | 0.000 |
| > 7 days | 3 | 5220 | 2.698 (0.949 - 7.671) | 89.9 | 0.000 | 0.063 |
| Intraoperative use of vasopressor | 13 | 4625 | 2.079 (1.492 - 2.899) | 70.3 | 0.000 | 0.000 |
| ≤ 7 days | 10 | 3478 | 1.996 (1.292 - 3.083) | 75.1 | 0.000 | 0.002 |
| > 7 days | 3 | 1147 | 2.183 (1.556 - 3.065) | 0.0 | 0.404 | 0.000 |
| Intraoperative RBC transfusion (per unit) | 15 | 8006 | 1.042 (1.025 - 1.059) | 76.9 | 0.000 | 0.000 |
| ≤ 7 days | 13 | 7529 | 1.044 (1.027 - 1.062) | 78.7 | 0.000 | 0.000 |
| > 7 days | 2 | 477 | 2.189 (0.281 - 17.052) | 71.7 | 0.060 | 0.455 |
| Large intraoperative RBC transfusion | 11 | 3401 | 3.124 (1.986 - 4.914) | 72.8 | 0.000 | 0.000 |
| ≤ 7 days | 7 | 2342 | 3.737 (2.363 - 5.909) | 61.6 | 0.016 | 0.000 |
| > 7 days | 4 | 1059 | 2.149 (0.681 - 6.789) | 83.6 | 0.000 | 0.192 |
| Intraoperative platelet transfusion (per unit) | 3 | 1503 | 1.321 (0.863 - 2.024) | 69.3 | 0.039 | 0.200 |
| ≤ 7 days | 2 | 1114 | 1.584 (1.235 - 2.033) | 0.0 | 0.597 | 0.000 |
| > 7 days | 1 | 389 | 0.920 (0.651 - 1.301) | - | - | 0.637 |
| Postreperfusion syndrome | 9 | 4731 | 1.689 (1.275 - 2.236) | 52.8 | 0.031 | 0.000 |
| ≤ 7 days | 8 | 4193 | 1.512 (1.174 - 1.948) | 38.6 | 0.122 | 0.001 |
| > 7 days | 1 | 538 | 3.120 (1.603 - 6.072) | - | - | 0.001 |
| Duration of operation (per hour) | 4 | 1563 | 1.158 (1.008 - 1.330) | 65.9 | 0.032 | 0.038 |
| ≤ 7 days | 3 | 1474 | 1.110 (0.985 - 1.251) | 56.7 | 0.099 | 0.088 |
| > 7 days | 1 | 89 | 1.547 (1.140 - 2.099) | 0.0 | - | 0.005 |
| Postoperative use of vasopressor | 4 | 4903 | 2.234 (1.431 - 3.488) | 75.9 | 0.006 | 0.000 |
| ≤ 7 days | 2 | 342 | 2.104 (1.048 - 4.224) | 68.8 | 0.073 | 0.037 |
| > 7 days | 2 | 4561 | 7.368 (0.357 - 152.013) | 88.5 | 0.003 | 0.196 |

**Supplementary 11: Subgroup analysis for factors with evident heterogeneity by statistical method**

| Factor | No. of study | Sample | OR (95% CI) | Heterogeneity | | P Value |
| --- | --- | --- | --- | --- | --- | --- |
|  |  |  |  | I^2^ | Chi^2^ |  |
| Age (per year) | 11 | 5476 | 1.006 (0.998 - 1.015) | 78.3 | 0.000 | 0.155 |
| Univariate analysis | 4 | 2675 | 1.008 (0.980 - 1.037) | 88.0 | 0.000 | 0.573 |
| Multivariate analysis | 7 | 2801 | 1.008 (0.996 - 1.020) | 61.5 | 0.016 | 0.210 |
| Female gender | 15 | 5399 | 1.479 (1.186 - 1.845) | 42.5 | 0.042 | 0.001 |
| Univariate analysis | 3 | 1409 | 1.360 (1.048 - 1.765) | 0.0 | 0.891 | 0.021 |
| Multivariate analysis | 12 | 3990 | 1.522 (1.124 - 2.061) | 54.3 | 0.012 | 0.007 |
| Hepatocellular carcinoma | 4 | 1027 | 0.681 (0.316 - 1.469) | 66.6 | 0.030 | 0.328 |
| Univariate analysis | 2 | 502 | 0.795 (0.245 - 2.583) | 75.4 | 0.044 | 0.703 |
| Multivariate analysis | 2 | 525 | 0.399 (0.047 - 3.371) | 79.5 | 0.027 | 0.399 |
| Preoperative hypertension | 10 | 5544 | 1.291 (0.814 - 2.045) | 64.7 | 0.003 | 0.278 |
| Univariate analysis | 4 | 1720 | 1.581 (0.627 - 3.983) | 82.3 | 0.001 | 0.331 |
| Multivariate analysis | 6 | 3824 | 1.104 (0.616 - 1.979) | 39.2 | 0.144 | 0.740 |
| Child-Turcotte-Pugh score (per score) | 5 | 2371 | 1.272 (1.115 - 1.452) | 73.2 | 0.005 | 0.000 |
| Univariate analysis | 2 | 1087 | 1.393 (0.708 - 2.740) | 53.4 | 0.143 | 0.338 |
| Multivariate analysis | 3 | 1284 | 1.350 (1.240 - 1.469) | 0.0 | 0.637 | 0.000 |
| MELD (per score) | 23 | 10444 | 1.035 (1.024 - 1.045) | 38.7 | 0.031 | 0.000 |
| Univariate analysis | 7 | 3760 | 1.026 (1.016 - 1.037) | 0.0 | 0.599 | 0.000 |
| Multivariate analysis | 16 | 6684 | 1.040 (1.025 - 1.056) | 47.4 | 0.016 | 0.000 |
| Preoperative serum creatinine (per mg/dL) | 6 | 2422 | 2.337 (1.215 - 4.497) | 81.0 | 0.000 | 0.011 |
| Univariate analysis | 1 | 193 | 4.260 (1.620 - 11.201) | - | - | 0.003 |
| Multivariate analysis | 5 | 2229 | 2.110 (1.017 - 4.380) | 83.1 | 0.000 | 0.045 |
| Donor age (per year) | 5 | 2170 | 1.004 (0.991 - 1.017) | 49.5 | 0.095 | 0.578 |
| Univariate analysis | 1 | 1136 | 1.020 (1.005 - 1.035) | - | - | 0.009 |
| Multivariate analysis | 4 | 1034 | 0.999 (0.989 - 1.009) | 0.0 | 0.416 | 0.870 |
| Long cold ischaemic time | 3 | 5220 | 1.408 (0.907 - 2.187) | 73.1 | 0.024 | 0.128 |
| Univariate analysis | 1 | 538 | 1.670 (1.099 - 2.538) | - | - | 0.016 |
| Multivariate analysis | 2 | 4682 | 1.374 (0.668 - 2.828) | 66.1 | 0.086 | 0.388 |
| Warm ischaemic time (per min) | 7 | 3427 | 1.018 (1.007 - 1.029) | 53.6 | 0.044 | 0.001 |
| Univariate analysis | 2 | 1240 | 1.010 (1.005 - 1.015) | 0.0 | 0.712 | 0.000 |
| Multivariate analysis | 5 | 2187 | 1.025 (1.009 - 1.041) | 52.5 | 0.077 | 0.002 |
| Piggyback surgical technique | 3 | 903 | 0.556 (0.195 - 1.585) | 81.8 | 0.004 | 0.272 |
| Univariate analysis | 1 | 409 | 0.342 (0.189 - 0.619) | - | - | 0.000 |
| Multivariate analysis | 2 | 494 | 0.749 (0.192 - 2.916) | 66.4 | 0.084 | 0.676 |
| Venovenous bypass | 3 | 740 | 0.577 (0.086 - 3.865) | 95.2 | 0.000 | 0.571 |
| Univariate analysis | 2 | 498 | 1.585 (0.976 - 2.575) | 0.0 | 0.350 | 0.063 |
| Multivariate analysis | 1 | 242 | 0.100 (0.050 - 0.200) | - | - | 0.000 |
| Large intraoperative blood loss | 6 | 5639 | 2.900 (1.495 - 5.627) | 83.1 | 0.000 | 0.002 |
| Univariate analysis | 2 | 199 | 3.636 (1.929 - 6.854) | 0.0 | 0.471 | 0.000 |
| Multivariate analysis | 4 | 5440 | 2.676 (1.155 - 6.198) | 86.0 | 0.000 | 0.022 |
| Intraoperative use of vasopressor | 13 | 4625 | 2.079 (1.492 - 2.899) | 70.3 | 0.000 | 0.000 |
| Univariate analysis | 4 | 2716 | 1.833 (1.102 - 3.050) | 77.2 | 0.004 | 0.020 |
| Multivariate analysis | 9 | 1909 | 2.207 (1.421 - 3.427) | 60.2 | 0.010 | 0.000 |
| Intraoperative RBC transfusion (per unit) | 15 | 8006 | 1.042 (1.025 - 1.059) | 76.9 | 0.000 | 0.000 |
| Univariate analysis | 4 | 2830 | 1.030 (1.025 - 1.059) | 0.0 | 0.431 | 0.000 |
| Multivariate analysis | 11 | 5176 | 1.061 (1.029 - 1.095) | 82.5 | 0.000 | 0.000 |
| Large intraoperative RBC transfusion | 11 | 3401 | 3.124 (1.986 - 4.914) | 72.8 | 0.000 | 0.000 |
| Univariate analysis | 3 | 258 | 4.048 (2.146 - 7.637) | 0.0 | 0.716 | 0.000 |
| Multivariate analysis | 8 | 3143 | 2.901 (1.673 - 5.030) | 79.2 | 0.000 | 0.000 |
| Intraoperative platelet transfusion (per unit) | 3 | 1503 | 1.321 (0.863 - 2.024) | 69.3 | 0.039 | 0.200 |
| Univariate analysis | 2 | 1387 | 1.210 (0.726 - 2.015) | 81.9 | 0.019 | 0.465 |
| Multivariate analysis | 1 | 116 | 1.948 (0.871 - 4.358) | - | - | 0.105 |
| Postreperfusion syndrome | 9 | 4731 | 1.689 (1.275 - 2.236) | 52.8 | 0.031 | 0.000 |
| Univariate analysis | 2 | 576 | 1.574 (0.868 - 2.852) | 36.4 | 0.210 | 0.135 |
| Multivariate analysis | 7 | 4155 | 1.760 (1.254 - 2.468) | 60.2 | 0.020 | 0.001 |
| Duration of operation (per hour) | 4 | 1563 | 1.158 (1.008 - 1.330) | 65.9 | 0.032 | 0.038 |
| Univariate analysis | 2 | 655 | 1.326 (1.086 - 1.618) | 40.6 | 0.195 | 0.006 |
| Multivariate analysis | 2 | 908 | 1.058 (0.938 - 1.193) | 33.1 | 0.222 | 0.361 |
| Postoperative use of vasopressor | 4 | 4903 | 2.234 (1.431 - 3.488) | 75.9 | 0.006 | 0.000 |
| Univariate analysis | 1 | 158 | 3.340 (1.549 - 7.204) | - | - | 0.002 |
| Multivariate analysis | 3 | 4745 | 2.021 (1.246 - 3.278) | 79.8 | 0.007 | 0.004 |

**Supplementary 12: Meta-regression of incidence and factors with evident heterogeneity**

Incidence (heterogeneity: I^2^ = 99.5%, P for Chi^2^ test = 0.000)

| Possible source of heterogeneity | P Value |
| --- | --- |
| Publication year | 0.489 |
| Diagnostic criteria |  |
| RIFLE | 0.112 |
| AKIN | 0.576 |
| KDIGO | Dropped |
| Other | 0.185 |

Age (per year) (heterogeneity: I^2^ = 78.3%, P for Chi^2^ test = 0.000)

| Possible source of heterogeneity | P Value |
| --- | --- |
| Diagnostic criteria |  |
| RIFLE | 0.000 |
| AKIN | Dropped |
| KDIGO | 0.000 |
| Other | 0.019 |
| Duration of evaluation |  |
| ≤ 7 days | 0.164 |
| > 7 days | 0.734 |
| NA | Dropped |
| Statistical method |  |
| Univariate analysis | Dropped |
| Multivariate analysis | 0.018 |

Female gender (heterogeneity: I^2^ = 42.5%, P for Chi^2^ test = 0.042)

| Possible source of heterogeneity | P Value |
| --- | --- |
| Diagnostic criteria |  |
| RIFLE | 0.893 |
| AKIN | 0.718 |
| KDIGO | 0.438 |
| Other | Dropped |
| Duration of evaluation |  |
| ≤ 7 days | 0.498 |
| > 7 days | Dropped |
| NA | 0.101 |
| Statistical method |  |
| Univariate analysis | Dropped |
| Multivariate analysis | 0.711 |

Preoperative hypertension (heterogeneity: I^2^ = 64.7%, P for Chi^2^ test = 0.003)

| Possible source of heterogeneity | P Value |
| --- | --- |
| Diagnostic criteria |  |
| RIFLE | 0.300 |
| AKIN | 0.905 |
| KDIGO | Dropped |
| Other | 0.432 |
| Duration of evaluation |  |
| ≤ 7 days | 0.737 |
| > 7 days | 0.858 |
| NA | Dropped |
| Statistical method |  |
| Univariate analysis | 0.508 |
| Multivariate analysis | Dropped |

MELD (per score) (heterogeneity: I^2^ = 38.7%, P for Chi^2^ test = 0.031)

| Possible source of heterogeneity | P Value |
| --- | --- |
| Diagnostic criteria |  |
| RIFLE | 0.000 |
| AKIN | Dropped |
| KDIGO | 0.456 |
| Other | 0.164 |
| Duration of evaluation |  |
| ≤ 7 days | 0.030 |
| > 7 days | 0.811 |
| NA | Dropped |
| Statistical method |  |
| Univariate analysis | 0.000 |
| Multivariate analysis | Dropped |

Intraoperative use of vasopressor (heterogeneity: I^2^ = 70.3%, P for Chi^2^ test = 0.000)

| Possible source of heterogeneity | P Value |
| --- | --- |
| Diagnostic criteria |  |
| RIFLE | 0.518 |
| AKIN | Dropped |
| KDIGO | 0.543 |
| Other | 0.803 |
| Duration of evaluation |  |
| ≤ 7 days | Dropped |
| > 7 days | 0.676 |
| Statistical method |  |
| Univariate analysis | Dropped |
| Multivariate analysis | 0.007 |

Intraoperative RBC transfusion (per unit) (heterogeneity: I^2^ = 76.9%, P for Chi^2^ test = 0.000)

| Possible source of heterogeneity | P Value |
| --- | --- |
| Diagnostic criteria |  |
| RIFLE | 0.853 |
| AKIN | 0.602 |
| KDIGO | 0.782 |
| Other | Dropped |
| Duration of evaluation |  |
| ≤ 7 days | Dropped |
| > 7 days | 0.334 |
| Statistical method |  |
| Univariate analysis | Dropped |
| Multivariate analysis | 0.916 |

Large intraoperative RBC transfusion (heterogeneity: I^2^ = 72.8%, P for Chi^2^ test = 0.000)

| Possible source of heterogeneity | P Value |
| --- | --- |
| Diagnostic criteria |  |
| RIFLE | 0.995 |
| AKIN | Dropped |
| KDIGO | 0.986 |
| Other | 0.642 |
| Duration of evaluation |  |
| ≤ 7 days | Dropped |
| > 7 days | 0.198 |
| Statistical method |  |
| Univariate analysis | 0.558 |
| Multivariate analysis | Dropped |

**Supplementary 13: Funnel plot and Egger test of factors described in ≥ 10 study**

Age (per year) (N = 11; P for Egger test = 0.200)

Female gender (N = 15; P for Egger test = 0.569)

Pre-existing diabetes mellitus (N = 13; P for Egger test = 0.075)

Preoperative hypertension (N = 10; P for Egger test = 0.627)

MELD (per score) (N = 23; P for Egger test = 0.139)

Intraoperative use of vasopressor (N = 13; P for Egger test = 0.179)

Intraoperative RBC transfusion (per unit) (N = 15; P for Egger test = 0.177)

Large intraoperative RBC transfusion (N = 11; P for Egger test = 0.153)

**Supplementary 14: Systematic review of factors described in only one study**

| Study | Factor | OR (95% CI) | Sample | NOS |
| --- | --- | --- | --- | --- |
| Gabriel 2001 [[1](#_ENREF_1)] | Preoperative urine output (per L/day) | 0.093 (0.021 - 0.409) | 28/232 | 6 |
| Gabriel 2001 [[1](#_ENREF_1)] | Preoperative MAP (per 10 mmHg) | 0.556 (0.349 - 0.887) | 28/232 | 6 |
| Gabriel 2001 [[1](#_ENREF_1)] | Preoperative AST (per unit) | 1.001 (1.000 - 1.001) | 28/232 | 6 |
| Sanchez 2004 [[2](#_ENREF_2)] | Postoperative ICU day (> 3) | 10.230 (5.600 - 18.660) | 87/637 | 6 |
| Guitard 2006 [[3](#_ENREF_3)] | Time to AST peak (> 20 h) | 3.930 (1.240 - 12.400) | 39/40 | 7 |
| Guitard 2006 [[3](#_ENREF_3)] | Time to ALT peak (> 24 h) | 2.770 (1.140 - 6.710) | 39/40 | 7 |
| Guitard 2006 [[3](#_ENREF_3)] | MBP at 6 hour post-OLT (> 70 mmHg) | 0.270 (0.080 - 0.850) | 39/40 | 7 |
| Wei 2006 [[4](#_ENREF_4)] | Operation modality | 0.468 (0.148 - 1.482) | 25/64 | 7 |
| Wei 2006 [[4](#_ENREF_4)] | Preoperative tuberculosis | 1.000 (0.998 - 1.003) | 25/64 | 7 |
| O'Riordan 2007 [[5](#_ENREF_5)] | Postoperative use of aminoglycoside | 2.500 (1.200 - 5.100) | 90/221 | 7 |
| O'Riordan 2007 [[5](#_ENREF_5)] | Rejection | 0.300 (0.200 - 0.500) | 90/221 | 7 |
| Shu 2007 [[6](#_ENREF_6)] | Postoperative intra-abdominal hypertension | 18.360 (1.720 - 195.800) | 14/48 | 7 |
| Shu 2007 [[6](#_ENREF_6)] | Postoperative relaparotomy | 0.190 (0.010 - 3.730) | 14/48 | 7 |
| Shu 2007 [[6](#_ENREF_6)] | Postoperative respiratory failure | 0.070 (0.002 - 2.190) | 14/48 | 7 |
| Iglesias 2010 [[7](#_ENREF_7)] | Rates of change of Scr (ΔScr/Δt) (mg/dL/day) | 0.990 (0.970 - 1.000) | 243/445 | 7 |
| Iglesias 2010 [[7](#_ENREF_7)] | Postoperative stroke volume (mL) | 1.006 (1.001 - 1.011) | 243/445 | 7 |
| Kundakci 2010 [[8](#_ENREF_8)] | Duration of anhepatic phase (min) | 1.020 (1.000 - 1.040) | 64/48 | 7 |
| Kundakci 2010 [[8](#_ENREF_8)] | Intraoperative acidosis | 0.277 (0.093 -0.825) | 64/48 | 7 |
| Zhu 2010 [[9](#_ENREF_9)] | Anesthesia time (hour) | 1.000 (1.000 - 1.010) | 116/77 | 7 |
| Liu 2013 [[10](#_ENREF_10)] | Use of steroid after OLT | 3.500 (0.810 - 14.800) | 43/346 | 7 |
| Romano 2013 [[11](#_ENREF_11)] | Preoperative infection | 3.400 (0.540 - 21.860) | 52/40 | 7 |
| Romano 2013 [[11](#_ENREF_11)] | In-hospital days prior surgery | 0.950 (0.810 - 1.130) | 52/40 | 7 |
| Klaus 2014 [[12](#_ENREF_12)] | Preoperative angiotensin-converting anzyme inhibitor (ACEI) use | 2.460 (0.820 - 7.390) | 64/49 | 6 |
| Klaus 2014 [[12](#_ENREF_12)] | Preoperative proteinuria | 3.750 (1.300 - 10.200) | 64/49 | 6 |
| Leithead 2014 [[13](#_ENREF_13)] | Non-alcoholic fatty liver disease (NAFLD) | 0.850 (0.490 - 1.480) | 381/771 | 7 |
| Leithead 2014 [[13](#_ENREF_13)] | Preoperative hyponatraemia | 1.610 (1.190 - 2.170) | 381/771 | 7 |
| Leithead 2014 [[13](#_ENREF_13)] | Donor time in ICU > 7 days | 1.360 (0.810 - 2.310) | 381/771 | 7 |
| Leithead 2014 [[13](#_ENREF_13)] | Donor cause of death trauma | 0.810 (0.570 - 1.170) | 381/771 | 7 |
| Nadeem 2014 [[14](#_ENREF_14)] | Infusion of greater than 3,200 ml of chloride-liberal fluids | 6.250 (2.690 - 14.500) | 57/101 | 7 |
| Nadeem 2014 [[14](#_ENREF_14)] | Postoperative serum chloride level (day 2, mmol/L) | 1.090 (1.010 - 1.180) | 57/101 | 7 |
| Sirivatanauksorn 2014 [[15](#_ENREF_15)] | Preoperative prothrombin time > 15 s | 2.810 (1.020 - 7.750) | 58/23 | 7 |
| Smoter 2014 [[16](#_ENREF_16)] | Cholestatic disorders | 0.249 (0.056 - 1.108) | 46/52 | 5 |
| Smoter 2014 [[16](#_ENREF_16)] | Intraoperative serum creatinine > 0.9 mg/dL | 1.650 (0.050 - 0.946) | 46/52 | 5 |
| Smoter 2014 [[16](#_ENREF_16)] | Postoperative serum creatinine 24 hours post-transplantation > 0.9 mg/dL | 6.386 (1.516 - 26.909) | 46/52 | 5 |
| Smoter 2014 [[16](#_ENREF_16)] | Urea 24 hours post-transplantation > 40 mg/dL | 45.674 (7.187 - 370.560) | 46/52 | 5 |
| Kim 2014 [[17](#_ENREF_17)] | Hepatic encephalopathy | 5.470 (1.930 - 15.520) | 42/115 | 5 |
| Barreto 2015 [[18](#_ENREF_18)] | Viral liver disease | 2.900 (1.200 - 7.000) | 64/70 | 7 |
| Barreto 2015 [[18](#_ENREF_18)] | Postoperative serum lactate within 24h after LT (mmol/L) | 1.300 (1.020 - 1.890) | 64/70 | 7 |
| Chen 2015 [[19](#_ENREF_19)] | Preoperative cerebrovascular diseases | 2.061 (1.117 - 3.804) | 214/2648 | 6 |
| Mukhtar 2015 [[20](#_ENREF_20)] | Intraoperative terlipressin therapy | 0.400 (0.200 - 0.800) | 115/188 | 7 |
| Park 2015 [[21](#_ENREF_21)] | Mean blood glucose during the day of surgery > 150 mg/dL | 1.540 (0.930 - 2.560) | 147/391 | 7 |
| Sang 2015 [[22](#_ENREF_22)] | Anesthetic time (min) | 1.002 (1.001 - 1.003) | 593/405 | 7 |
| Sang 2015 [[22](#_ENREF_22)] | Preoperative MBP (mmHg) | 0.980 (0.970 - 0.990) | 593/405 | 7 |
| Sang 2015 [[22](#_ENREF_22)] | Preoperative platelet count (×10^3^/µL) | 1.000 (1.000 - 1.000) | 593/405 | 7 |
| Inoue 2016 [[23](#_ENREF_23)] | Cytomegalovirus (CMV) infection | 0.846 (0.389 - 1.836) | 78/40 | 5 |
| Inoue 2016 [[23](#_ENREF_23)] | Hyperlipidemia | 0.247 (0.022 - 2.808) | 78/40 | 5 |
| Wadei 2016 [[24](#_ENREF_24)] | Waiting time (per day) | 0.990 (0.990 - 1.000) | 71/1178 | 7 |
| Wadei 2016 [[24](#_ENREF_24)] | Hepatic steatosis | 1.110 (0.490 - 2.510) | 71/1178 | 7 |
| Wiesen 2016 [[25](#_ENREF_25)] | Postoperative bilirubin level (maximal, mg/L) | 1.440 (1.010 - 2.050) | 109/78 | 7 |
| Wiesen 2016 [[25](#_ENREF_25)] | Postoperative hemoglobin level (mininmum, g/dL) | 0.060 (0.010 - 0.290) | 109/78 | 7 |
| Chae 2017 [[26](#_ENREF_26)] | C-reactive protein (mg/L) | 1.250 (1.068 - 1.463) | 76/258 | 7 |
| Chae 2017 [[26](#_ENREF_26)] | Intraoperative use of diuretic (mg) | 1.018 (1.008 - 1.027) | 76/258 | 7 |
| Chen 2017 [[27](#_ENREF_27)] | Preoperative prothrombin time (sec) | 1.060 (0.990 - 1.140) | 109/457 | 7 |
| Chen 2017 [[27](#_ENREF_27)] | Anhepatic time (hour) | 3.590 (1.370 - 9.390) | 109/457 | 7 |
| Kalisvaart 2017 [[28](#_ENREF_28)] | Preoperative serum sodium (mmol/L) | 1.008 (0.948 - 1.072) | 61/94 | 7 |
| Kalisvaart 2017 [[28](#_ENREF_28)] | Coronary artery disease | 1.249 (0.322 - 4.848) | 61/94 | 7 |
| Kalisvaart 2017 [[28](#_ENREF_28)] | Hepatorenal syndrome | 2.307 (0.697 - 7.633) | 61/94 | 7 |
| Mizota 2017 [[29](#_ENREF_29)] | Intraoperative nadir MAP (per 10 mmHg decrease) | 2.110 (1.320 - 3.470) | 71/160 | 7 |
| Mizota 2017 [[29](#_ENREF_29)] | Intraoperative relative decrease in MAP (per 10% decrease) | 1.510 (1.110 - 2.090) | 71/160 | 7 |
| Mizota 2017 [[29](#_ENREF_29)] | Intraoperative nadir CI (per 1 L/min/m^2^ decrease) | 0.940 (0.660 - 1.350) | 71/160 | 7 |
| Mizota 2017 [[29](#_ENREF_29)] | Intraoperative relative decrease in CI (per 10% decrease) | 1.210 (1.010 - 1.460) | 71/160 | 7 |
| Rahman 2017 [[30](#_ENREF_30)] | Noradrenaline infusion rate on ICU arrival (μg/kg per minute) | 47.468 (0.120 - 1836.270) | 58/58 | 7 |
| Yoo 2017 [[31](#_ENREF_31)] | Pulmonary hypertension | 3.630 (1.060 - 12.440) | 132/172 | 7 |
| Yoo 2017 [[31](#_ENREF_31)] | Increased perioperative glucose variability | 2.160 (1.050 - 4.420) | 132/172 | 7 |
| Codes 2018 [[32](#_ENREF_32)] | Cumulative fluid balance in the first 4 day (mL) | 2.300 (1.370 - 3.860) | 87/34 | 7 |
| Kalisvaart 2018 [[33](#_ENREF_33)] | Donor female gender | 0.943 (0.570 - 1.559) | 239/129 | 7 |
| Kwon 2018 [[34](#_ENREF_34)] | Early postoperative MAP (by decrease of 10 mmHg) | 1.360 (1.160 - 1.590) | 777/359 | 7 |
| Kwon 2018 [[34](#_ENREF_34)] | Cystatin C GFR (by decrease of 10 ml/min/1.73 m^2^) | 1.050 (1.010 - 1.100) | 777/359 | 7 |
| Wang 2018 [[35](#_ENREF_35)] | Transfusion of older blood | 2.470 (1.130 - 5.410) | 71/66 | 7 |
| Carrier 2019 [[36](#_ENREF_36)] | Fluid balance (L) | 1.140 (0.970 - 1.330) | 31/496 | 7 |
| Kim 2019 [[37](#_ENREF_37)] | Previous abdominal surgery | 1.120 (1.040 - 1.240) | 265/265 | 7 |
| Kim 2019 [[37](#_ENREF_37)] | Baseline central venous pressure (per 5 cmH_2_O increase) | 1.200 (1.090 - 1.320) | 265/265 | 7 |
| Kim 2019 [[37](#_ENREF_37)] | Mixed venous oxygen saturation at 5 min before reperfusion (per 10% decrease) | 1.450 (1.270 - 1.710) | 265/265 | 7 |
| Kim 2019 [[37](#_ENREF_37)] | Baseline right ventricular end-diastolic volume (per 10 ml increase) | 1.480 (1.240 - 1.780) | 265/265 | 7 |
| Yu 2019 [[38](#_ENREF_38)] | Preoperative beta-blocker administration | 2.000 (0.860 - 4.140) | 342/543 | 7 |

**NOTE:** OR, odds ratio; 95% CI, 95% confidence interval; NOS, Newcastle-Ottawa Scale; MAP, mean arterial pressure; AST, aspartate transaminase; ICU, intensive care unit; ALT, alamine aminotransferase; MBP, mean blood pressure; OLT, orthotopic liver transplantation; Scr, serum creatinine; LT, liver transplantation; CI, cardiac index; GFR, glomerular filtration rate.

**Reference:**

1. Gabriel C GG, Andrew A. Q,Cynthia C, Mark A. L., Gaspar A. B.,David R., Emilio G,: **An Epidemiologic Study of Early Renal Replacement Therapy after Orthotopic Liver Transplantation**. *J Am Soc Nephrol* 2001, **13**:228–233.

2. Sanchez EQ, Gonwa TA, Levy MF, Goldstein RM, Mai ML, Hays SR, Melton LB, Saracino G, Klintmalm GB: **Preoperative and perioperative predictors of the need for renal replacement therapy after orthotopic liver transplantation**. *Transplantation* 2004, **78**(7):1048-1054.

3. Guitard J, Cointault O, Kamar N, Muscari F, Lavayssière L, Suc B, Ribes D, Esposito L, Barange K, Durand D *et al*: **Acute renal failure following liver transplantation with induction therapy**. *Clin Nephrol* 2006, **65**(2):103-112.

4. Wei Y, Zhang L, Lin H, Li J, Li B, Yan L, Wen T, Zeng Y, Lu S: **Factors related to post-liver transplantation acute renal failure**. *Transplant Proc* 2006, **38**(9):2982-2984.

5. O'Riordan A, Wong V, McQuillan R, McCormick PA, Hegarty JE, Watson AJ: **Acute renal disease, as defined by the RIFLE criteria, post-liver transplantation**. *Am J Transplant* 2007, **7**(1):168-176.

6. Shu M, Peng C, Chen H, Shen B, Zhou G, Shen C, Li H: **Intra-abdominal hypertension is an independent cause of acute renal failure after orthotopic liver transplantation**. *Front Med China* 2007, **1**(2):167-172.

7. Iglesias JI, DePalma JA, Levine JS: **Risk factors for acute kidney injury following orthotopic liver transplantation: the impact of changes in renal function while patients await transplantation**. *BMC Nephrol* 2010, **11**:30-30.

8. Kundakci A, Pirat A, Komurcu O, Torgay A, Karakayalı H, Arslan G, Haberal M: **Rifle criteria for acute kidney dysfunction following liver transplantation: incidence and risk factors**. *Transplant Proc* 2010, **42**(10):4171-4174.

9. Zhu M, Li Y, Xia Q, Wang S, Qiu Y, Che M, Dai H, Qian J, Ni Z, Axelsson J *et al*: **Strong impact of acute kidney injury on survival after liver transplantation**. *Transplant Proc* 2010, **42**(9):3634-3638.

10. Liu S, Wang X, Lu Y, Li T, Gong Z, Sheng T, Hu B, Peng Z, Sun X: **The effects of intraoperative cryoprecipitate transfusion on acute renal failure following orthotropic liver transplantation**. *Hepatol Int* 2013, **7**(3):901-909.

11. Romano TG, Schmidtbauer I, Silva FMdQ, Pompilio CE, D'Albuquerque LAC, Macedo E: **Role of MELD score and serum creatinine as prognostic tools for the development of acute kidney injury after liver transplantation**. *PLoS One* 2013, **8**(5):e64089-e64089.

12. Klaus F, Keitel da Silva C, Meinerz G, Carvalho LM, Goldani JC, Cantisani G, Zanotelli ML, Duro Garcia V, Keitel E: **Acute kidney injury after liver transplantation: incidence and mortality**. *Transplant Proc* 2014, **46**(6):1819-1821.

13. Leithead JA, Rajoriya N, Gunson BK, Muiesan P, Ferguson JW: **The evolving use of higher risk grafts is associated with an increased incidence of acute kidney injury after liver transplantation**. *J Hepatol* 2014, **60**(6):1180-1186.

14. Nadeem A, Salahuddin N, El Hazmi A, Joseph M, Bohlega B, Sallam H, Sheikh Y, Broering D: **Chloride-liberal fluids are associated with acute kidney injury after liver transplantation**. *Crit Care* 2014, **18**(6):625-625.

15. Sirivatanauksorn Y, Parakonthun T, Premasathian N, Limsrichamrern S, Mahawithitwong P, Kositamongkol P, Tovikkai C, Asavakarn S: **Renal dysfunction after orthotopic liver transplantation**. *Transplant Proc* 2014, **46**(3):818-821.

16. Smoter P, Nyckowski P, Grat M, Patkowski W, Zieniewicz K, Wronka K, Hinderer B, Morawski M: **Risk factors of acute renal failure after orthotopic liver transplantation: single-center experience**. *Transplant Proc* 2014, **46**(8):2786-2789.

17. Kim JM, Jo YY, Na SW, Kim SI, Choi YS, Kim NO, Park JE, Koh SO: **The predictors for continuous renal replacement therapy in liver transplant recipients**. *Transplant Proc* 2014, **46**(1):184-191.

18. Barreto AGC, Daher EF, Silva Junior GB, Garcia JHP, Magalhães CBA, Lima JMC, Viana CFG, Pereira EDB: **Risk factors for acute kidney injury and 30-day mortality after liver transplantation**. *Ann Hepatol* 2015, **14**(5):688-694.

19. Chen H-P, Tsai Y-F, Lin J-R, Liu F-C, Yu H-P: **Incidence and Outcomes of Acute Renal Failure Following Liver Transplantation: A Population-Based Cohort Study**. *Medicine (Baltimore)* 2015, **94**(52):e2320-e2320.

20. Mukhtar A, Mahmoud I, Obayah G, Hasanin A, Aboul-Fetouh F, Dabous H, Bahaa M, Abdelaal A, Fathy M, El Meteini M: **Intraoperative terlipressin therapy reduces the incidence of postoperative acute kidney injury after living donor liver transplantation**. *J Cardiothorac Vasc Anesth* 2015, **29**(3):678-683.

21. Park MH, Shim HS, Kim WH, Kim H-J, Kim DJ, Lee S-H, Kim CS, Gwak MS, Kim GS: **Clinical Risk Scoring Models for Prediction of Acute Kidney Injury after Living Donor Liver Transplantation: A Retrospective Observational Study**. *PLoS One* 2015, **10**(8):e0136230-e0136230.

22. Sang B-H, Bang J-Y, Song J-G, Hwang G-S: **Hypoalbuminemia Within Two Postoperative Days Is an Independent Risk Factor for Acute Kidney Injury Following Living Donor Liver Transplantation: A Propensity Score Analysis of 998 Consecutive Patients**. *Crit Care Med* 2015, **43**(12):2552-2561.

23. Inoue Y, Soyama A, Takatsuki M, Hidaka M, Kinoshita A, Natsuda K, Baimakhanov Z, Kugiyama T, Adachi T, Kitasato A *et al*: **Does the development of chronic kidney disease and acute kidney injury affect the prognosis after living donor liver transplantation?** *Clin Transplant* 2016, **30**(5):518-527.

24. Wadei HM, Lee DD, Croome KP, Mai ML, Golan E, Brotman R, Keaveny AP, Taner CB: **Early Allograft Dysfunction After Liver Transplantation Is Associated With Short- and Long-Term Kidney Function Impairment**. *Am J Transplant* 2016, **16**(3):850-859.

25. Wiesen P, Massion PB, Joris J, Detry O, Damas P: **Incidence and risk factors for early renal dysfunction after liver transplantation**. *World J Transplant* 2016, **6**(1):220-232.

26. Chae MS, Lee N, Park DH, Lee J, Jung HS, Park CS, Lee J, Choi JH, Hong SH: **Influence of oxygen content immediately after graft reperfusion on occurrence of postoperative acute kidney injury in living donor liver transplantation**. *Medicine (Baltimore)* 2017, **96**(31):e7626-e7626.

27. Chen X, Ding X, Shen B, Teng J, Zou J, Wang T, Zhou J, Chen N, Zhang B: **Incidence and outcomes of acute kidney injury in patients with hepatocellular carcinoma after liver transplantation**. *J Cancer Res Clin Oncol* 2017, **143**(7):1337-1346.

28. Kalisvaart M, de Haan JE, Hesselink DA, Polak WG, Hansen BE, Ijzermans JNM, Gommers D, Metselaar HJ, de Jonge J: **The postreperfusion syndrome is associated with acute kidney injury following donation after brain death liver transplantation**. *Transpl Int* 2017, **30**(7):660-669.

29. Mizota T, Hamada M, Matsukawa S, Seo H, Tanaka T, Segawa H: **Relationship Between Intraoperative Hypotension and Acute Kidney Injury After Living Donor Liver Transplantation: A Retrospective Analysis**. *J Cardiothorac Vasc Anesth* 2017, **31**(2):582-589.

30. Rahman S, Davidson BR, Mallett SV: **Early acute kidney injury after liver transplantation: Predisposing factors and clinical implications**. *World J Hepatol* 2017, **9**(18):823-832.

31. Yoo S, Lee H-J, Lee H, Ryu H-G: **Association Between Perioperative Hyperglycemia or Glucose Variability and Postoperative Acute Kidney Injury After Liver Transplantation: A Retrospective Observational Study**. *Anesth Analg* 2017, **124**(1):35-41.

32. Codes L, de Souza YG, D'Oliveira RAC, Bastos JLA, Bittencourt PL: **Cumulative positive fluid balance is a risk factor for acute kidney injury and requirement for renal replacement therapy after liver transplantation**. *World J Transplant* 2018, **8**(2):44-51.

33. Kalisvaart M, Schlegel A, Umbro I, de Haan JE, Scalera I, Polak WG, Ijzermans JNM, Mirza DF, Perera MTPR, Isaac JI *et al*: **The Impact of Combined Warm Ischemia Time on Development of Acute Kidney Injury in Donation After Circulatory Death Liver Transplantation: Stay Within the Golden Hour**. *Transplantation* 2018, **102**(5):783-793.

34. Kwon H-M, Moon Y-J, Jung K-W, Jeong H-W, Park Y-S, Jun I-G, Song J-G, Hwang G-S: **Low Mean Arterial Blood Pressure is Independently Associated with Postoperative Acute Kidney Injury After Living Donor Liver Transplantation: A Propensity Score Weighing Analysis**. *Ann Transplant* 2018, **23**:236-245.

35. Wang Y, Li Q, Ma T, Liu X, Wang B, Wu Z, Dang S, Lv Y, Wu R: **Transfusion of Older Red Blood Cells Increases the Risk of Acute Kidney Injury After Orthotopic Liver Transplantation: A Propensity Score Analysis**. *Anesth Analg* 2018, **127**(1):202-209.

36. Carrier FM, Chassé M, Sylvestre M-P, Girard M, Legendre-Courville L, Massicotte L, Bilodeau M: **Effects of intraoperative fluid balance during liver transplantation on postoperative acute kidney injury: an observational cohort study**. *Transplantation* 2019:10.1097/TP.0000000000002998.

37. Kim WH, Oh H-W, Yang S-M, Yu JH, Lee H-C, Jung C-W, Suh K-S, Lee KH: **Intraoperative Hemodynamic Parameters and Acute Kidney Injury After Living Donor Liver Transplantation**. *Transplantation* 2019, **103**(9):1877-1886.

38. Yu JH, Kwon Y, Kim J, Yang S-M, Kim WH, Jung C-W, Suh K-S, Lee KH: **Influence of Transfusion on the Risk of Acute Kidney Injury: ABO-Compatible versus ABO-Incompatible Liver Transplantation**. *J Clin Med* 2019, **8**(11):1785.
